# Clinical Evidence of Tai Chi Exercise Prescriptions: A Systematic Review

**DOI:** 10.1155/2021/5558805

**Published:** 2021-03-10

**Authors:** Jiafu Huang, Dandan Wang, Jinghao Wang

**Affiliations:** School of Physical Education & Sports Science, South China Normal University, Guangzhou 510006, China

## Abstract

**Objectives:**

This systematic review aims to summarize the existing literature on Tai Chi randomized controlled trials (RCTs) and recommend Tai Chi exercise prescriptions for different diseases and populations.

**Methods:**

A systematic search for Tai Chi RCTs was conducted in five electronic databases (PubMed, Cochrane Library, EMBASE, EBSCO, and Web of Science) from their inception to December 2019. SPSS 20.0 software and Microsoft Excel 2019 were used to analyze the data, and the risk of bias tool in the RevMan 5.3.5 software was used to evaluate the methodological quality of RCTs.

**Results:**

A total of 139 articles were identified, including diseased populations (95, 68.3%) and healthy populations (44, 31.7%). The diseased populations included the following 10 disease types: musculoskeletal system or connective tissue diseases (34.7%), circulatory system diseases (23.2%), mental and behavioral disorders (12.6%), nervous system diseases (11.6%), respiratory system diseases (6.3%), endocrine, nutritional or metabolic diseases (5.3%), neoplasms (3.2%), injury, poisoning and certain other consequences of external causes (1.1%), genitourinary system diseases (1.1%), and diseases of the eye and adnexa (1.1%). Tai Chi exercise prescription was generally classified as moderate intensity. The most commonly applied Tai Chi style was Yang style (92, 66.2%), and the most frequently specified Tai Chi form was simplified 24-form Tai Chi (43, 30.9%). 12 weeks and 24 weeks, 2-3 times a week, and 60 min each time was the most commonly used cycle, frequency, and time of exercise in Tai Chi exercise prescriptions.

**Conclusions:**

We recommend the more commonly used Tai Chi exercise prescriptions for different diseases and populations based on clinical evidence of Tai Chi. Further clinical research on Tai Chi should be combined with principles of exercise prescription to conduct large-sample epidemiological studies and long-term prospective follow-up studies to provide more substantive clinical evidence for Tai Chi exercise prescriptions.

## 1. Introduction

Chronic diseases cause a large burden of disease in the world and affect the quality of life of individuals [1, 2]. Furthermore, almost half of the global population suffers from at least one chronic disease and may be at risk of functional decline and disability [3, 4]. Exercise therapy is a safe way to improve physical function and reduce disability in patients with chronic diseases [5]. In the past few decades, exercise has been widely used in the treatment of chronic diseases, and experts have begun to adopt the terms “exercise prescription” and “exercise therapy” [5]. Exercise prescription mainly includes the type, frequency, duration, and intensity of exercise. It is a scientific, systematic, and individualized exercise intervention program for disease prevention and health promotion.

Tai Chi is a complementary and alternative therapy that has become a widespread exercise worldwide [6]. Over the past two decades, researchers have conducted extensive studies of the health-promoting effects of Tai Chi by focusing on various systems of the human body and their corresponding diseases. Studies have shown that Tai Chi is beneficial for patients with a wide range of diseases [7] and is a safe and effective way to promote balance control, flexibility, and cardiovascular fitness in patients with chronic diseases [8]. Tai Chi has been widely used in the clinical practice of different diseases and people, and their positive effects have been confirmed, but there is no clear guidance on how to incorporate it into exercise prescriptions [9]. A study adopted an evaluation instrument to reveal the characteristics of Tai Chi exercise prescriptions for improving the balance ability of the elderly, but this study has not yet conducted studies on Tai Chi exercise prescriptions for different diseases and populations [10]. In addition, Tai Chi has been shown to be effective in treating diverse diseases, but such treatment effects are not consistent across studies, such as type 2 diabetes. [11–13]. This may be affected by the difference in Tai Chi style and exercise time in Tai Chi exercise prescriptions because a study has confirmed that different Tai Chi styles and exercise time can result in variable effectiveness [14].

A systematic review involves identifying a specific problem, systematically collecting relevant articles, determining which articles meet the predetermined inclusion criteria, evaluating previous reports, implementing rigorous scientific analysis, and forming a reliable comprehensive conclusion for reference in clinical application [15]. A systematic review is the basis of evidence-based medicine and is considered at the highest level of medical evidence [16]. Randomized controlled trials (RCTs) are generally considered to have the highest level of credibility and hence are considered the gold standard for evidence-based clinical practice [17]. Therefore, the purpose of this systematic review was to summarize and analyze the effects of Tai Chi exercise prescriptions in Tai Chi RCTs, in order to recommend common Tai Chi exercise prescriptions for different diseases and individuals and provide a reference for the clinical application and experimental research on Tai Chi exercise prescription.

## 2. Materials and Methods

### 2.1. Search Strategy

Five electronic databases (PubMed, Cochrane Library, EMBASE, EBSCO, and Web of Science) were searched for relevant studies published up until December 2019. The search terms used for this systematic review included “Tai Chi,” “Tai Chi Chuan,” “T'ai Chi,” “Tai Ji,” “Taiji,” and “Tai Ji Quan.” The language was restricted to English. Taking a specific strategy as an example, the search terms in the PubMed database were as follows: ((((((Tai Chi [Title/Abstract]) OR (Tai Chi Chuan [Title/Abstract])) OR (T'ai Chi [Title/Abstract])) OR (Tai Ji[Title/Abstract])) OR (Taiji [Title/Abstract])) OR (Tai Ji Quan [Title/Abstract]) AND (humans[Filter])) AND ((randomized controlled trial[Publication Type] OR randomized[Title/Abstract] OR placebo[Title/Abstract]) AND (humans[Filter])).

### 2.2. Inclusion and Exclusion Criteria

This study strictly followed the PRISMA statement and the principle of PICOS (participants, intervention, control, outcomes, and study design) to formulate the criteria for literature retrieval, inclusion, screening, and exclusion [18].

#### 2.2.1. Inclusion Criteria

Studies included in this review had to meet the following inclusion criteria: (1) the trial was a randomized control trial (RCT), either individual or cluster randomized; (2) any study participants were included, regardless of region, age, gender, and current health condition; (3) Tai Chi was the main intervention, without a limitation based on the type of Tai Chi prescribed; (4) the control group was nonexercise or the exercise was not Tai Chi; (5) outcome indicators were not restricted.

#### 2.2.2. Exclusion Criteria

Studies were excluded if they met any of the following criteria: (1) irrelevant discussion or application of Tai Chi or interventions that lacked Tai Chi; (2) duplicate studies; (3) review, case report, meeting abstract, or monograph; (4) unclear outcome indicators; (5) no data or incomplete data; (6) not RCT.

### 2.3. Study Selection and Data Extraction

For this systematic review, two researchers independently read the titles, abstracts, and full text of research studies and conducted literature screening and data extraction according to the above inclusion and exclusion criteria. Disagreements were resolved either by consensus between the two researchers or by asking a third researcher to make a final decision. The following data were extracted: first author, publication year, study design, participants, intervention characteristics (i.e., type, duration, time, frequency), and outcome measures.

### 2.4. Quality Assessment

Based on the bias risk assessment tool recommended by the Cochrane Systematic Review Handbook (2011) [19], two researchers independently assessed the methodological quality and the bias risk of each included literature. The assessed items included the presence of random sequence generation, allocation concealment, blinding, outcome assessors, incomplete outcome data, selective reporting, and the presence of other biases. Based on these criteria, the literature included was divided into the high risk of bias, low risk of bias, and unclear risk of bias categories. Disagreements between the two researchers were resolved by discussion with a third researcher.

### 2.5. Data Analysis

Due to the heterogeneity of the included studies, a meta-analysis was not conducted. We performed descriptive data analysis using SPSS 20.0 and Microsoft Excel 2019. Data were presented in terms of counts, percentage, and frequency.

## 3. Results

### 3.1. Selection of Studies

**Figure 1:**
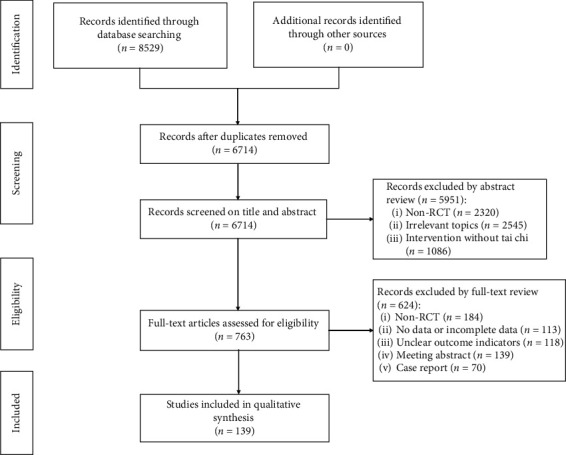
Flow diagram of literature selection.

A total of 8529 articles were obtained according to the search strategy. These articles were imported into EndNote X9, and duplicates were eliminated. The remaining 6714 articles were then subject to the screening on the basis of the title and abstract, resulting in the exclusion of 5951 studies. The remaining 763 articles were further screened by reading the full-text article and 624 articles were excluded. Finally, this review included 139 qualitative studies (see Figure 1).

### 3.2. Study Characteristics

A total of 139 articles were included in this review. Participants included patients with various diseases as well as healthy middle-aged and elderly individuals. The interventions for the experimental group included Yang style, Sun style, Chen style, Wu style, and unspecified styles of Tai Chi. The interventions for the control groups included exercise control, routine nursing care, daily activities, health education, and more. The cycle of Tai Chi exercise varied from 4 to 48 weeks, the frequency of Tai Chi exercise ranged from one to seven times per week, and the duration of Tai Chi exercise varied from 30 to 120 min. Outcome measures included different physical and psychological indicators and corresponding test scales, such as Berg Balance Scale, Pittsburgh Sleep Quality of Index, and QoL Short Form 36-Item Health Survey. More detailed information is shown in Table S1.

### 3.3. Quality Assessment of Eligible Studies

**Figure 2:**
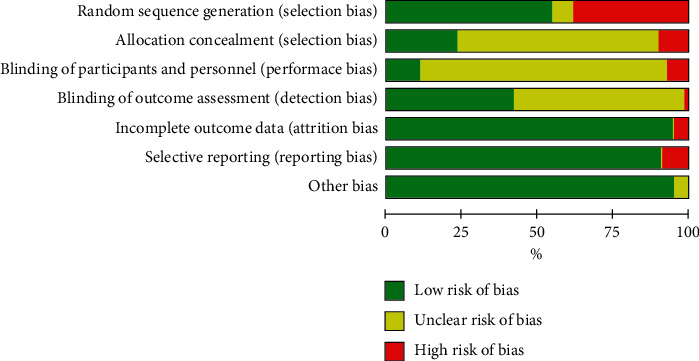
Risk of bias graph.

For the random allocation method, 66 studies in this paper used random number tables and computer-based random number generators to produce random sequences, with 35 studies described as “random grouping” with potential high-risk bias. In terms of allocation concealment, 73 studies did not describe their allocation concealment schemes while 33 studies employed methods such as opaque envelopes or containers and independent allocation for allocation concealment. Most studies did not describe whether researchers, participants, and assessors were blinded, with only 14 studies blinding researchers or participants and 61 studies blinding outcome assessors. Studies included for analysis that appeared to contain incomplete outcome data, selective reporting, and other types of bias generally had low risks of bias (see Figures 2 and S1).

### 3.4. Disease/Condition Categories

The 139 studies included in this review were categorized as involving diseased populations (95, 68.3%) and healthy populations (44, 31.7%), based on the types of participants. According to the 10^th^ Revision of the International Statistical Classification of Diseases and Related Health Problems (ICD-10) [20], the diseased populations were classified as belonging to one of 10 disease types (see Table 1): musculoskeletal system or connective tissue diseases (34.7%), circulatory system diseases (23.2%), mental and behavioral disorders (12.6%), nervous system diseases (11.6%), respiratory system diseases (6.3%), endocrine, nutritional or metabolic diseases (5.3%), neoplasms (3.2%), injury, poisoning and certain other consequences of external causes (1.1%), genitourinary system diseases (1.1%), and diseases of the eye and adnexa (1.1%). The top 10 most prevalent diseases were osteoarthritis, heart failure, stroke, Parkinson's disease, chronic obstructive pulmonary disease (COPD), depression, fibromyalgia, diabetes, coronary heart disease, and hypertension (see Table 2).

### 3.5. Classification of Tai Chi Styles

Table 3 shows that the Tai Chi styles in the included 139 studies include the Yang style, Sun style, Chen style, Wu style, and some unspecified styles of Tai Chi. In the 139 studies included, 92 studies (66.2%) chose Yang style, 13 studies (9.4%) chose Sun style, 6 studies (4.3%) chose Chen style, 2 studies (1.4%) chose Wu style, and 26 studies (18.7%) did not specify the exact style. Among the 92 studies (66.2%) which selected Yang style Tai Chi, 43 studies (30.9%) chose a simplified 24-form Tai Chi. In addition, some studies only described the style but not the form of Tai Chi used, while other studies included more than one style of Tai Chi intervention.

### 3.6. Tai Chi Exercise Prescriptions

Among the 139 Tai Chi RCTs included in this review, there were certain differences in the style, exercise cycle, frequency, time, and intensity of Tai Chi included in exercise prescriptions for different disease systems and healthy individuals. This systematic review will, therefore, summarize the exercise intervention programs for diseased and healthy populations and determine the most commonly used Tai Chi exercise prescriptions for different diseases and populations based on clinical evidence and the exercise prescription principles of the American College of Sports Medicine (ACSM). The supplementary tables in various sections of this review describe Tai Chi exercise prescriptions for specific groups of patients and healthy individuals.

#### 3.6.1. Musculoskeletal System or Connective Tissue Diseases

Musculoskeletal system or connective tissue diseases mainly include osteoarthritis, fibromyalgia, osteoporosis, chronic low back pain, and neck pain, among which osteoarthritis is one of the most common and is a leading cause of disability worldwide [21]. In the United States, 27 million people are affected by osteoarthritis [22], and its associated costs reach around $188.5 billion each year [23]. Osteoporosis is especially prevalent in postmenopausal women, mainly causing hip and spine fractures leading to high morbidity and mortality rates [24]. Chronic low back pain is a common musculoskeletal injury and can be caused by overload or incorrect movement patterns at work, during exercise, or as a result of various accidents [25]. Low back pain is one of the leading causes of disability and a major contributor to the global burden of disease [26]. Studies have shown that chronic diseases such as osteoarthritis, osteoporosis, and chronic low back pain have no effective cure, and their clinical management is extremely difficult. Tai Chi is an ancient form of Chinese mind-body exercise, and many studies have confirmed its effectiveness in treating musculoskeletal system or connective tissue diseases such as osteoarthritis, fibromyalgia, and chronic low back pain.

Table S2 shows that the styles of Tai Chi commonly used to treat the musculoskeletal system or connective tissue diseases include Yang style, Sun style, Chen style, and some unspecified styles of Tai Chi. The most prevalent of these adopted Tai Chi styles was Yang style (21, 63.6%), in which the simplified 24-form Tai Chi (7, 21.2%) was the most popular Tai Chi form, followed by the 10-form Yang style Tai Chi (5, 15.1%). The simplified 24-form Tai Chi can improve physical function, relieve pain symptoms, and improve the quality of life of patients with osteoarthritis [27–30]. The simplified 24-form Tai Chi is also an effective option for osteoporosis patients because it improves dynamic balance, reduces the risk of falls [31], and improves the quality of life [32]. A 12-week period of the 10-form Yang style Tai Chi training 2-3 times a week can reduce the pain of fibromyalgia patients and is thus an effective rehabilitation therapy of fibromyalgia [33, 34]. Practicing the 12-form Sun style Tai Chi can relieve pain symptoms and improve physical function and quality of life in patients with osteoarthritis [35–38]. Moreover, Chen style Tai Chi practice 3 times each week, 60 min per session, and for 12 weeks has a positive effect on neuromuscular function and reduces the chronic pain in patients with nonspecific chronic low back pain [39].

In short, the styles of Tai Chi described above have therapeutic effects on the musculoskeletal system or connective tissue diseases. Yang style Tai Chi is the most used Tai Chi style, and the simplified 24-form Tai Chi is the most used form. Tai Chi training in the cycle of 12 weeks, 60 min each time, 2-3 times a week is the most used exercise prescription.

#### 3.6.2. Circulatory System Diseases

Circulatory system diseases, also called cardiocerebral vascular system diseases, mainly include hypertension, chronic heart failure, coronary heart disease, stroke, and myocardial infarction. Hypertension is one of the common diseases threatening human health and is a major risk factor for cardiovascular events, stroke, and death [40]. In recent years, the incidence of circulatory system diseases has rapidly risen, with gradually increasing occurrence in young people [41].

Table S3 shows that the styles of Tai Chi effective in treating circulatory system diseases include Yang style, Sun style, Wu style, and some unspecified styles of Tai Chi. Among them, the most frequently used Tai Chi style was Yang style (17, 77.3%), and the most frequently used Tai Chi forms were the simplified 24-form Tai Chi (5, 22.7%) and 5-form Tai Chi (5, 22.7%), both of which belong to Yang style. The moderate-intensity simplified 24-form Tai Chi can reduce blood pressure levels, control weight, and improve both metabolism and quality of life in patients with hypertension [42]. The simplified 24-form Tai Chi can also improve the physical and mental health of people with hypertension and reduce the risk of cardiovascular disease [43, 44]. A 12-week, twice a week, 60-minute 5-form Yang style Tai Chi exercise can improve the quality of life and physical function of patients with chronic heart failure [45–47] and enhance sleep stability [46, 48]. For patients with coronary heart disease, the 8-form Yang style Tai Chi can enhance baroreflex sensitivity (BRS) [49], and the 108-form Yang style Tai Chi can improve the peak heart rate-pressure product (RPP) and RPP reserve [50]. In addition, Yang style Tai Chi can reduce the risk of falls [51], improve cognitive ability [52, 53], and improve sleep quality and depression symptoms in stroke patients [53]. Another study showed that Sun style Tai Chi can improve the standing balance ability of stroke patients [54].

In summary, Tai Chi has a positive effect on circulatory system diseases such as hypertension, stroke, coronary heart disease, and chronic heart failure. For these patients, a 12-week, 2-3 times a week, 60-minute Yang style Tai Chi exercise is a feasible exercise program.

#### 3.6.3. Mental and Behavioral Disorders

Mental and behavioral disorders mainly include depression, cognitive impairment, and intellectual disabilities. Depression is one of the most common mental disorders among the elderly [55] and is expected to cause a prominent disease burden in developed and developing countries by 2030 [56]. The World Health Organization (WHO) estimates that 121 million elderly people suffer from depression worldwide [57].

Table S4 shows that the Yang style, Sun style, and Wu style are commonly used styles of Tai Chi recommended for patients with mental and behavioral disorders. Among them, the Yang style (7, 58.3%) was the most commonly used Tai Chi style, with the most commonly used Tai Chi form being the simplified 24-form Tai Chi (4, 33.3%). For depression, the simplified 24-form Tai Chi can reduce depression levels [58, 59] and improve the quality of life [60]. Two studies have confirmed that Tai Chi can improve clinical outcomes [61], reduce symptoms of anxiety and stress, and enhance leg strength in patients with depression [62]. Yang style Tai Chi can improve cognitive function and memory in patients with cognitive impairment [63, 64]. Additionally, 20 weeks of 12-form Sun style Tai Chi training can relieve knee pain in patients with cognitive impairment [65]. Another study showed that Tai Chi training for 15 weeks, 3 times a week, 50 min each time can effectively reduce the risk of falls and improve cognitive function in the elderly with mild cognitive impairment [66]. A 12-week, 3 times a week, 60-minute moderate-intensity Wu style Tai Chi training can improve the motor coordination and memory of patients with chronic schizophrenia [67].

To sum up, Yang style Tai Chi is a commonly used Tai Chi style for patients with mental and behavioral disorders. For these patients, a 12-week or longer, 2-3 times a week, 60-minute Tai Chi training is a safe and feasible exercise program.

#### 3.6.4. Nervous System Diseases

Nervous system diseases mainly include Parkinson's disease, dementia, and sleep disorders, which are often associated with motor control and cognitive dysfunction [41]. Most of these diseases have high disability rates that seriously affect the quality of life of patients [41]. Studies have shown that Tai Chi is a safe and effective exercise for patients with nervous system diseases [68].

As shown in Table S5, the styles of Tai Chi used to treat nervous system diseases included Yang style, Chen Style, Sun style, and some unspecified styles of Tai Chi. Of them, the most commonly used style was Yang style Tai Chi (7, 63.6%), and the most commonly selected forms were simplified 24-form Tai Chi and 6-form Yang style Tai Chi. For Parkinson's disease, Yang style Tai Chi can improve balance and motor function [69, 70] and enhance the health-related cognitive ability of patients [71]. Practicing 12-form Sun style Tai Chi can alleviate disease symptoms and improve the quality of life and physical function of patients with Parkinson's disease [72]. Two other studies have demonstrated that a 12-week, 3 times a week, 60-minute moderate-intensity Tai Chi exercise can improve the functional status of Parkinson's patients [73, 74]. In addition, Chen style Tai Chi can improve the quality of life and reduce the risk of falls in patients with dementia [75]. Yang style Tai Chi also improved the sleep quality of patients with moderate sleep disorders [76].

In summary, Tai Chi is a safe and effective way to improve the lives of patients with nervous system diseases such as Parkinson's disease, dementia, and sleep disorders. For these patients, a 12-week or longer, 2-3 times per week, 60-minute Yang style Tai Chi practice is the most used exercise prescription.

#### 3.6.5. Respiratory System Diseases

Respiratory system diseases are common diseases that seriously endanger human health [77]. For this review, we focused on the chronic obstructive pulmonary disease (COPD). COPD is a progressive lung disease and a common cause of death among adults worldwide [78]. The main features of this disease are chronic cough, cough with sputum, dyspnea, wheezing, and chest tightness [41]. At present, COPD intervention methods mainly involve drug therapy, pulmonary rehabilitation, and exercise therapy [79].

As shown in Table S6, the styles of Tai Chi used to treat COPD in studies we examined were Yang style, Sun style, and some unspecified style of Tai Chi. A 12-week Yang style Tai Chi exercise can improve the exercise capacity, reduce the symptoms of dyspnea [80, 81], and improve the quality of life in patients with COPD [82]. Practicing 60 min of moderate-intensity Sun style Tai Chi twice a week for 12 weeks can improve the exercise capacity, balance ability, and quality of life of patients with COPD [83].

In summary, a 12-week, twice a week, 60-minute Yang style or Sun style Tai Chi practice is conducive to the improvement of symptoms in patients with COPD.

#### 3.6.6. Endocrine, Nutritional, or Metabolic Diseases

Metabolic system diseases affect human health and quality of life and have a prominent worldwide concern [41]. The endocrine, nutritional, or metabolic diseases focused on in this review included type 2 diabetes and metabolic syndrome. Epidemiological investigations showed that there were around 371 million diabetic patients as of 2012, with this number expected to increase to 552 million by 2030 [84, 85]. Type 2 diabetes accounts for more than 90% of the diabetes cases globally and places a huge burden on health systems [86]. Moreover, metabolic syndrome, a cluster of risk factors for type 2 diabetes and cardiovascular disease, has increasingly become a global health problem [87].

As Table S7 shows, the styles of Tai Chi used to treat endocrine, nutritional, or metabolic diseases included Yang style, KaiMai style, and multiple styles of Tai Chi. Practicing 60 min each time of moderate-intensity simplified 24-form Tai Chi exercise 5 times a week for 14 weeks was a beneficial intervention program that improved glycemic control, triglyceride levels, resting blood pressure, and heart rate in patients with type 2 diabetes [88]. In addition, KaiMai style Tai Chi can also improve the physical function and blood sugar control and reduce the body pain of patients with type 2 diabetes [89]. Notably, a combination of Yang style and Sun style Tai Chi did not produce a positive effect on patients with type 2 diabetes, which may have been due to insufficient intensity, frequency, or duration of practices [90, 91]. However, multiple styles of Tai Chi, including Yang style and Sun style, show benefits for middle-aged male office workers with metabolic syndrome [92].

To summarize, a 12-week or longer, 2-3 times a week, 60-minute Yang style Tai Chi training had a significant effect on patients with metabolic system diseases. However, the Tai Chi exercise volume needs to be adjusted based on individual patient reactions.

#### 3.6.7. Neoplasms

The studies involving patients with neoplasms reviewed for this study focused on lung cancer and breast cancer. Cancer is a major public health problem worldwide and the second leading cause of mortality in the United States [93]. According to an American Cancer Society report, 1,806,590 new cancer cases and 606,520 cancer deaths are projected to occur in the United States in 2020 [93]. There is growing scientific evidence that exercise is an effective way to prevent cancer development [94]. Several studies have demonstrated that Tai Chi can enhance the physical functions and improve the quality of life of cancer survivors [95–97].

As shown in Table S8, Yang style Tai Chi and some unspecified style of Tai Chi were the exercise methods used to treat cancer in reports we studied. A 16-week, 3 times a week, 60-minute low-intensity simplified 24-form Tai Chi training significantly attenuated CD55 expression but did not alter the CD4+ : CD8+ ratio in lung cancer survivors [98]. In addition, a 12-week, 4 times a week, 60-minute 8-form Yang style Tai Chi training effectively alleviated physical fatigue and enhanced vitality among patients with lung cancer [99]. Another study showed that Tai Chi is a useful exercise for improving the physical and mental state of female patients with breast cancer [100].

In summary, practicing 60 min of Yang style Tai Chi 3 times a week for 12 weeks or longer has a positive effect on cancer patients.

#### 3.6.8. Other Disease Conditions

Table S9 reveals that a 90-minute 8-form Yang style Tai Chi training 3 times a week for 16 weeks can improve the balance ability of elderly patients with visual impairment [101]. In addition, a 6-week, 3 times a week, 45-minute Chen style Tai Chi training can regulate the mood of individuals with traumatic brain injury [102]. The results of another study showed that 60 min of Tai Chi training 3 times a week for 12 weeks can improve lower urinary tract symptoms and quality of life in patients with benign prostate hypertrophy [103].

Although this clinical evidence has shown that Tai Chi has a positive effect on the abovementioned diseases, due to the limited number of studies conducted, more robust RCTs are required to further confirm these results.

#### 3.6.9. Healthy Populations

Among the 44 Tai Chi RCTs involving healthy populations included in this review, most of the subjects were middle-aged and elderly people. Various physical functions of middle-aged and elderly people gradually decline with age. The decreased balance increases the risk of falls and fractures in individuals over the age of 65 [104, 105]. Approximately one-third of community residents aged 65 or older fall at least once a year [106], and the risk of falling increases with age [107]. Studies have shown that regular long-term Tai Chi exercises increase balance control and flexibility and can reduce the risk of falls in older adults [108].

As Table S10 shows, the styles of Tai Chi used by healthy people include the Yang style, Chen style, and some unspecified styles of Tai Chi. Among them, Yang style Tai Chi was the most frequently selected style (34, 77.3%), and the most commonly used Tai Chi form was the simplified 24-form Tai Chi (22, 50%). For healthy people, the most common cycle of Tai Chi exercise was 24 weeks (15/44, 34.1%), followed by 12 weeks (11/44, 25%) and 16 weeks (7/44, 15.9%). The frequency and duration of Tai Chi exercise generally adopted by healthy people were 2-3 times a week for 60 min, respectively. The results of several studies showed that performing simplified 24-form Tai Chi can improve the balance and posture control and reduce the risk of falls of the elderly [109–115]. The simplified 24-form Tai Chi can also improve self-efficacy [116, 117] and quality of life [118] and relieve stress in the elderly [119]. In addition, 60 min of Chen style Tai Chi training 3 times a week for 20 weeks can improve balance ability [120], leg strength [121], and antibody response to influenza vaccine in older adults [122].

In summary, for the majority of healthy people, a 24-week, 2-3 times a week, 60-minute simplified 24-form Tai Chi training is a safe and effective exercise program. Long-term regular Tai Chi training can improve various physical functions and the quality of life in older adults.

## 4. Discussion

### 4.1. Summary of Evidence

Tai Chi exercise has been widely regarded as an effective method for health promotion, adjuvant treatment for chronic diseases, and a form of rehabilitation therapy during the recovery period from diseases. In this systematic review, the effects of Tai Chi on improving health-related parameters in both healthy populations and diseased populations were examined comprehensively. A total of 139 RCTs were included in this systematic review; 44 studies focused on the effects of Tai Chi on the physical function, cognitive function, quality of life, balance ability, and psychological indicators in healthy middle-aged and elderly people and the effects of Tai Chi in preventing falls. Besides, 95 studies focused on the effects of Tai Chi on different diseases. We have summarized several styles of Tai Chi interventions and their uses in the treatment of various diseases (Table 4). Tai Chi has potential clinical value for treating many diseases, especially noncommunicable chronic diseases. The most common diseases were in the musculoskeletal system or connective tissue and the circulatory system. This may be due to the physiological and biomechanical that are influenced by Tai Chi training [123].

At present, Tai Chi styles used as treatments mainly include the Yang style, Chen style, Sun style, and Wu style. Different styles of Tai Chi have their own characteristics. For instance, Yang style Tai Chi has the characteristics of smooth rhythms and soft movements and requires a moderate volume of exercise, which renders it more suitable for people of different ages and physical conditions. Therefore, Yang style Tai Chi was most commonly applied in the studies and clinical trials. The ACSM suggests that an exercise prescription should include aerobic, resistance, flexibility, and neuromotor exercise training to effectively maintain and improve the physical fitness and health of most people [124]. Tai Chi is a traditional Chinese mind-body exercise, and a large number of studies have confirmed that Tai Chi has a significant effect on improving aerobic capacity, muscle strength, flexibility, balance, and postural control [108, 125–127]. In addition, the training characteristics of Tai Chi also meet the ACSM exercise prescription guidelines regarding maintaining and developing cardiorespiratory endurance, muscle strength, flexibility, and balance [128]. This shows that Tai Chi is a multimodal exercise that combines aerobic exercise, resistance exercise, flexibility training, and neuromotor training to create a comprehensive effect on the human body [129].

The research in this review revealed that there are certain differences in the period, frequency, and time of Tai Chi exercises for different diseases or individuals. However, 60 min of Tai Chi training 2-3 times a week for 12 or 24 weeks is the most frequently selected exercise program. For patients, 12 weeks of moderate-intensity Tai Chi training can be adopted in the early and middle stages of a disease. With the alleviation of disease symptoms, the amount of Tai Chi exercise should be adjusted according to the patient's physical condition. For healthy populations, the ACSM recommends moderate-intensity aerobic exercises for a minimum of 30 min, five times each week, or vigorous-intensity aerobic exercise for a minimum of 20 min, three times each week [130]. It is thus currently recommended that healthy populations who perform 60 min of moderate-intensity Tai Chi exercises 2-3 times a week can meet ACSM recommended exercise volume. However, in order to further improve physical fitness and reduce the risk of chronic disease and disability, regular exercise routines should not be limited to the minimum exercise volumes recommended by ACSM [130]. Furthermore, avoiding injury is more important than engaging in exercise [41]. The formulation of Tai Chi exercise prescription should follow the principle of gradual progress, and exercise volume should be dynamically adjusted according to the health and exercise response of individuals.

With regard to exercise intensity evaluation, 14 studies [42, 43, 60, 67, 73, 74, 83, 88, 90, 98, 120, 131–133] described the intensity of Tai Chi used in Tai Chi exercise prescriptions. Exercise intensity is measured using indicators including the rating of perceived exertion (RPE) [73, 74, 83, 90, 131, 132], percent of maximum heart rate (HR_max_) [42, 88, 98, 133], and percent of maximum oxygen uptake (VO2_max_) [42, 67]. According to the ACSM's classification standard of exercise intensity [124], Tai Chi exercise prescription is generally classified as moderate intensity. However, the exercise intensity of Tai Chi depends on its training style, movement posture, and duration, with different training styles leading to substantial differences in exercise intensity [68]. Tai Chi exercise intensity thus still needs further study in order for medical professionals to more accurately recommend Tai Chi exercise intensity well suited for people of different health statuses.

### 4.2. Practical Implications

This review systematically evaluated the Tai Chi exercise programs performed by diseased and healthy populations. We summarized the effects of different Tai Chi exercise programs on different disease systems and populations and ultimately recommend the more commonly used Tai Chi exercise prescriptions. According to the latest report from the WHO in 2018, chronic diseases cause 41 million deaths each year, equivalent to 71% of the global deaths [134]. Notably, physical inactivity is associated with a range of chronic diseases and early death [135] and has enormous health and economic consequences [136]. Tai Chi has potential benefits as a low-cost exercise because equipment and facilities are not needed. In addition, Tai Chi is unlikely to result in serious adverse events [137]. Therefore, from the perspective of disease prevention, Tai Chi is very suitable for promotion and application in the community as an ideal form of exercise for the elderly. From the perspective of clinical applications, Tai Chi has potential therapeutic value for chronic diseases such as musculoskeletal system or connective tissue diseases, circulatory system diseases, and nervous system diseases. However, although Tai Chi plays an important role in disease prevention and health promotion, more high methodological quality research is needed to determine more accurate Tai Chi exercise prescriptions.

### 4.3. Future Research

First, most RCTs on Tai Chi we analyzed generally utilized Yang style Tai Chi, especially the simplified 24-form Tai Chi. Future research involving other Tai Chi styles should be conducted to provide more sufficient clinical evidence for the formulation of Tai Chi exercise prescriptions. Second, the majority of the Tai Chi studies have focused on the musculoskeletal system or connective tissue and circulatory system diseases, and the subjects were mainly middle-aged and elderly people. Further research should include other age groups and more disease types. Third, the descriptions of Tai Chi in clinical research need to be improved, and information such as the Tai Chi styles and forms, exercise cycle, duration, frequency, learning method, practice method, the occurrence of adverse events, follow-up, and other aspects of Tai Chi practice should be provided. Fourth, exercise intensity is a core element of exercise prescription, yet few studies provide any detailed information on the intensity of Tai Chi exercise. Further studies are needed to evaluate the dose-effect relationship between Tai Chi exercise intensity and exercise effect. Finally, Tai Chi has been promoted as a life-long practice. Most exercise cycles described in Tai Chi RCTs lasted for 12 weeks or 24 weeks. Further studies should consist of large-sample epidemiological studies and long-term prospective follow-up studies to better evaluate the relationship between the long-term practice of Tai Chi and the reduction in the risks associated with chronic disease.

### 4.4. Limitations

This systematic review has some limitations. First, we only included English documents published in the English language databases, which may affect the comprehensiveness of the data surveyed. Second, there were few blind RCTs in the included literature, which may bias the study results. Third, due to the limited amount of literature included, this review grouped evidence for the effectiveness of Tai Chi exercise prescriptions based on disease systems rather than specific diseases, which may affect the accuracy of our Tai Chi exercise prescriptions.

## 5. Conclusions

Tai Chi exercise prescription is a feasible and effective method for preventing diseases and promoting health. In this systematic review, we summarized Tai Chi exercise prescriptions for different disease systems and people and recommended the more commonly used Tai Chi style, cycle, frequency, and time of exercise. Further clinical research on Tai Chi should be combined with the principles of exercise prescription to conduct large-sample epidemiological studies and long-term prospective follow-up studies to provide more substantive clinical evidence for Tai Chi exercise prescriptions.

## Figures and Tables

**Table 1:** Diseased populations of disease categories based on ICD-10 classifications.

Disease/conditions (ICD-10codes)	No. of studies (%)
Diseases of the musculoskeletal system or connective tissue	33 (34.7)
Diseases of the circulatory system	22 (23.2)
Mental and behavioral disorders	12 (12.6)
Diseases of the nervous system	11 (11.6)
Diseases of the respiratory system	6 (6.3)
Endocrine, nutritional, or metabolic diseases	5 (5.3)
Neoplasms	3 (3.2)
Injury, poisoning, and certain other consequences of external causes	1 (1.1)
Diseases of the genitourinary system	1 (1.1)
Diseases of the eye and adnexa	1 (1.1)

**Table 2:** Top 10 diseases/conditions included in clinical studies on Tai Chi.

Disease/condition	No. of studies (%)
Osteoarthritis	15 (15.8)
Heart failure	7 (7.4)
Stroke	6 (6.3)
Parkinson's disease	6 (6.3)
Chronic obstructive pulmonary disease	6 (6.3)
Depression	6 (6.3)
Fibromyalgia	5 (5.3)
Diabetes	4 (4.2)
Coronary heart disease	4 (4.2)
Hypertension	4 (4.2)

**Table 3:** Tai Chi styles of the Included Studies (*n* = 139).

Tai Chi style	No. of studies	Frequency (%)
**Yang style**	**92**	**66.2**
Simplified 24-form	43	30.9
10-form Yang style	10	7.2
Unspecified forms of Yang style	9	6.5
8-form Yang style	7	5.0
5-form Yang style	7	5.0
108-form Yang style	5	3.6
12-form Yang style	3	2.2
13-form Yang style	3	2.2
18-form Yang style	2	1.4
6-form Yang style	2	1.4
9-form Yang style	1	0.7

**Sun style**	**13**	**9.4**
12-form Sun style	7	5.0
21-form Sun style	3	2.2
31-form Sun style	1	0.7
24-form Sun style	1	0.7
5-form Sun style	1	0.7

**Chen style**	**6**	**4.3**
Unspecified forms of Chen style	2	1.4
7-form Chen style	2	1.4
12-form Chen style	1	0.7
5-form Chen style	1	0.7

**Wu style**	**2**	**1.4**
22-form Wu style	1	0.7
Unspecified forms of Wu style	1	0.7

**Others**	**26**	**18.7**
Unspecified style	20	14.4
Multiple styles	5	3.6
KaiMai style	1	0.7

**Table 4:** Styles of Tai Chi for various diseases.

Tai Chi styles	Diseases
Yang style	OA; NLBP; OP; fibromyalgia; ACL; chronic nonspecific neck pain; stroke; hypertension; myocardial infarction; HF; CHD; depression; mild cognitive impairment; intellectual disability; tension-type headaches; dizziness; PD; sleep disorder; dementia; COPD; T2D; lung cancer; visual impairment
Sun style	OA; NLBP; ankylosing spondylitis; stroke; mild cognitive impairment; PD; COPD
Chen style	NLBP; dementia; traumatic brain injury
Wu style	HF; chronic schizophrenia

Note: ACL = anterior cruciate ligament; CHD = coronary heart disease; COPD = chronic obstructive pulmonary disease; HF = heart failure; NLBP = nonspecific chronic lower back pain; OA = osteoarthritis; OP = osteoporosis; PD = Parkinson's disease; T2D = type 2 diabetes.

## Data Availability

All data generated or analyzed during this study are included within this article.

## Supplementary Materials

Supplementary Materials Table S1: basic characteristics of the included studies. Table S2: musculoskeletal system or connective tissue diseases. Table S3: circulatory system diseases. Table S4: mental and behavioral disorders. Table S5: nervous system diseases. Table S6: respiratory system diseases. Table S7: endocrine, nutritional, or metabolic diseases. Table S8: neoplasms. Table S9: other disease conditions. Table S10: healthy populations. Figure S1: risk of bias summary.

## References

[1] Coates M. M., Kintu A., Gupta N. (2020). Burden of non-communicable diseases from infectious causes in 2017: a modelling study. *Lancet Global Health*.

[2] Parker L., Moran G. M., Roberts L. M., Calvert M., McCahon D. (2014). The burden of common chronic disease on health-related quality of life in an elderly community-dwelling population in the UK. *Family Practice*.

[3] Barnett K., Mercer S. W., Norbury M., Watt G., Wyke S., Guthrie B. (2012). Epidemiology of multimorbidity and implications for health care, research, and medical education: a cross-sectional study. *The Lancet*.

[4] Violan C., Foguet-Boreu Q., Flores-Mateo G. (2014). Prevalence, determinants and patterns of multimorbidity in primary care: a systematic review of observational studies. *PLoS One*.

[5] Pasanen T., Tolvanen S., Heinonen A., Kujala U. M. (2017). Exercise therapy for functional capacity in chronic diseases: an overview of meta-analyses of randomised controlled trials. *British Journal of Sports Medicine*.

[6] Pan Y., Yang K., Shi X., Liang H., Zhang F., Lv Q. (2015). Tai Chi Chuan exercise for patients with breast cancer: a systematic review and meta-analysis. *Evidence-Based Complementary and Alternative Medicine*.

[7] Lee M. S., Lee E.-N., Ernst E. (2009). Is Tai Chi beneficial for improving aerobic capacity? a systematic review. *British Journal of Sports Medicine*.

[8] Wang C., Collet J. P., Lau J. (2004). The effect of tai chi on health outcomes in patients with chronic conditions. *Archives of Internal Medicine*.

[9] Zhu W. M. (2020). The past, present and future of exercise prescription. *Sports Science Research*.

[10] Wu Y., MacDonald H. V., Pescatello L. S. (2016). Evaluating exercise prescription and instructional methods used in tai chi studies aimed at improving balance in older adults: a systematic review. *Journal of the American Geriatrics Society*.

[11] Lee M. S., Choi T.-Y., Lim H.-J., Ernst E. (2011). Tai Chi for management of type 2 diabetes mellitus: a systematic review. *Chinese Journal of Integrative Medicine*.

[12] Lee M. S., Jun J. H., Lim H.-J., Lim H.-S. (2015). A systematic review and meta-analysis of Tai Chi for treating type 2 diabetes. *Maturitas*.

[13] Chao M., Wang C., Dong X., Ding M. (2018). The effects of tai chi on type 2 diabetes mellitus: a meta-analysis. *Journal of Diabetes Research*.

[14] Xia T.-W., Yang Y., Li W.-H., Tang Z.-H., Li Z.-R., Qiao L.-J. (2019). Different training durations and styles of tai chi for glucose control in patients with type 2 diabetes: a systematic review and meta-analysis of controlled trials. *BMC Complementary and Alternative Medicine*.

[15] Jin Y. H., Wu S. W., Bai Z. G., Zeng X. T. (2019). Connotations and application of systematic review and meta-analysis: a briefly introduction. *Journal of Tongji University (Medical Science)*.

[16] Lauche R., Cramer H., Häuser W., Dobos G., Langhorst J. (2015). A systematic overview of reviews for complementary and alternative therapies in the treatment of the fibromyalgia syndrome. *Evidence-Based Complementary and Alternative Medicine*.

[17] Li J.-Y., Zhang Y.-F., Smith G. S. (2011). Quality of reporting of randomized clinical trials in tai chi interventions-a systematic review. *Evidence-Based Complementary and Alternative Medicine*.

[18] Liberati A., Altman D. G., Tetzlaff J. (2009). The PRISMA statement for reporting systematic reviews and meta-analyses of studies that evaluate health care interventions: explanation and elaboration. *PLoS Medicine*.

[19] Higgins JPT, Green S. (2020). *Cochrane Handbook for Systematic Reviews of Interventions Version 5.1.0*.

[20] World Health Organization (2016). *International Statistical Classification of Diseases and Related Health Problems 10^th^ Revision (ICD-10)-WHO Version for*.

[21] Clarson L. E., Nicholl B. I., Bishop A., Daniel R., Mallen C. D. (2016). Discussing prognosis with patients with osteoarthritis: a cross-sectional survey in general practice. *Clinical Rheumatology*.

[22] Lawrence R. C., Felson D. T., Helmick C. G. (2008). Estimates of the prevalence of arthritis and other rheumatic conditions in the United States: Part II. *Arthritis & Rheumatism*.

[23] Kotlarz H., Gunnarsson C. L., Fang H., Rizzo J. A. (2009). Insurer and out-of-pocket costs of osteoarthritis in the US: evidence from national survey data. *Arthritis & Rheumatism*.

[24] Christenson E. S., Jiang X., Kagan R., Schnatz P. (2012). Osteoporosis management in post-menopausal women. *Minerva Ginecologica*.

[25] Golob A. L., Wipf J. E. (2014). Low back pain. *Medical Clinics of North America*.

[26] Maher C., Underwood M., Buchbinder R. (2017). Non-specific low back pain. *The Lancet*.

[27] Lü J., Huang L., Wu X., Fu W., Liu Y. (2017). Effect of Tai Ji Quan training on self-reported sleep quality in elderly Chinese women with knee osteoarthritis: a randomized controlled trail. *Sleep Medicine*.

[28] Zhu Q., Huang L., Wu X. (2016). Effects of Tai Ji Quan training on gait kinematics in older Chinese women with knee osteoarthritis: a randomized controlled trial. *Journal of Sport and Health Science*.

[29] Brismée J.-M., Paige R. L., Chyu M.-C. (2007). Group and home-based tai chi in elderly subjects with knee osteoarthritis: a randomized controlled trial. *Clinical Rehabilitation*.

[30] Ni G.-X., Song L., Yu B., Huang C.-H., Lin J.-H. (2010). Tai chi improves physical function in older Chinese women with knee osteoarthritis. *JCR: Journal of Clinical Rheumatology*.

[31] Maciaszek J., Osiński W., Szeklicki R., Stemplewski R. (2007). Effect of Tai Chi on body balance: randomized controlled trial in men with osteopenia or osteoporosis. *The American Journal of Chinese Medicine*.

[32] Chyu M.-C., James C. R., Sawyer S. F. (2010). Effects of tai chi exercise on posturography, gait, physical function and quality of life in postmenopausal women with osteopaenia: a randomized clinical study. *Clinical Rehabilitation*.

[33] Wang C., Schmid C. H., Rones R. (2010). A randomized trial of tai chi for fibromyalgia. *New England Journal of Medicine*.

[34] Wong A., Figueroa A., Sanchez-Gonzalez M. A., Son W.-M., Chernykh O., Park S.-Y. (2018). Effectiveness of tai chi on cardiac autonomic function and symptomatology in women with fibromyalgia: a randomized controlled trial. *Journal of Aging and Physical Activity*.

[35] Tsai P.-F., Chang J. Y., Beck C., Kuo Y.-F., Keefe F. J. (2013). A pilot cluster-randomized trial of a 20-week tai chi program in elders with cognitive impairment and osteoarthritic knee: effects on pain and other health outcomes. *Journal of Pain and Symptom Management*.

[36] Song R., Lee E. O., Lam P., Bae S. C. (2007). Effects of a Sun-style Tai Chi exercise on arthritic symptoms, motivation and the performance of health behaviors in women with osteoarthritis. *Journal of Korean Academy of Nursing*.

[37] Song R., Lee E. O., Lam P., Bae S. C. (2003). Effects of Tai Chi exercise on pain, balance, muscle strength, and perceived difficulties in physical functioning in older women with osteoarthritis: a randomized clinical trial. *The Journal of Rheumatology*.

[38] Callahan L. F., Cleveland R. J., Altpeter M., Hackney B. (2016). Evaluation of tai chi program effectiveness for people with arthritis in the community: a randomized controlled trial. *Journal of Aging and Physical Activity*.

[39] Zou L., Zhang Y., Liu Y. (2019). The effects of tai chi chuan versus core stability training on lower-limb neuromuscular function in aging individuals with non-specific chronic lower back pain. *Medicina*.

[40] Weber M. A., Schiffrin E. L., White W. B. (2014). Clinical practice guidelines for the management of hypertension in the community. *The Journal of Clinical Hypertension*.

[41] Luan X., Tian X., Zhang H. (2019). Exercise as a prescription for patients with various diseases. *Journal of Sport and Health Science*.

[42] Shou X.-L., Wang L., Jin X.-Q., Zhu L.-Y., Ren A.-H., Wang Q.-N. (2019). Effect of T’ai Chi exercise on hypertension in young and middle-aged in-service staff. *The Journal of Alternative and Complementary Medicine*.

[43] Chan A. W. K., Chair S. Y., Lee D. T. F. (2018). Tai Chi exercise is more effective than brisk walking in reducing cardiovascular disease risk factors among adults with hypertension: a randomised controlled trial. *International Journal of Nursing Studies*.

[44] Ma C., Zhou W., Tang Q., Huang S. (2018). The impact of group-based Tai Chi on health-status outcomes among community-dwelling older adults with hypertension. *Heart & Lung*.

[45] Yeh G. Y., Wood M. J., Lorell B. H. (2004). Effects of tai chi mind-body movement therapy on functional status and exercise capacity in patients with chronic heart failure: a randomized controlled trial. *The American Journal of Medicine*.

[46] Yeh G. Y., Wayne P. M., Phillips R. S. (2008). T’ai Chi exercise in patients with chronic heart failure. *Medicine and Sport Science*.

[47] Yeh G. Y., McCarthy E. P., Wayne P. M. (2011). Tai Chi exercise in patients with chronic heart failure. *Archives of Internal Medicine*.

[48] Yeh G. Y., Mietus J. E., Peng C.-K. (2008). Enhancement of sleep stability with Tai Chi exercise in chronic heart failure: preliminary findings using an ECG-based spectrogram method. *Sleep Medicine*.

[49] Sato S., Makita S., Uchida R., Ishihara S., Masuda M. (2010). Effect of Tai Chi training on baroreflex sensitivity and heart rate variability in patients with coronary heart disease. *International Heart Journal*.

[50] Chang R.-Y., Koo M., Kan C.-B. (2010). Effects of Tai Chi rehabilitation on heart rate responses in patients with coronary artery disease. *The American Journal of Chinese Medicine*.

[51] Taylor-Piliae R. E., Hoke T. M., Hepworth J. T., Latt L. D., Najafi B., Coull B. M. (2014). Effect of Tai Chi on physical function, fall rates and quality of life among older stroke survivors. *Archives of Physical Medicine and Rehabilitation*.

[52] Chan W.-N., Tsang W. W.-N. (2017). Effect of tai chi training on dual-tasking performance that involves stepping down among stroke survivors: a pilot study. *Evidence-Based Complementary and Alternative Medicine*.

[53] Wang W., Sawada M., Noriyama Y. (2010). Tai Chi exercise versus rehabilitation for the elderly with cerebral vascular disorder: a single-blinded randomized controlled trial. *Psychogeriatrics*.

[54] Au-Yeung S. S. Y., Hui-Chan C. W. Y., Tang J. C. S. (2009). Short-form Tai Chi improves standing balance of people with chronic stroke. *Neurorehabilitation and Neural Repair*.

[55] Lebowitz B. D., Pearson J. L., Schneider L. S. (1997). Diagnosis and treatment of depression in late life. *JAMA*.

[56] Baiyewu O., Smith-Gamble V., Lane K. A. (2007). Prevalence estimates of depression in elderly community-dwelling African Americans in Indianapolis and Yoruba in Ibadan, Nigeria. *International Psychogeriatrics*.

[57] Alexopoulos G. S. (2005). Depression in the elderly. *The Lancet*.

[58] Zhang J., Qin S., Zhou Y., Meng L., Su H., Zhao S. (2018). A randomized controlled trial of mindfulness-based Tai Chi Chuan for subthreshold depression adolescents. *Neuropsychiatric Disease and Treatment*.

[59] Liu J., Xie H., Liu M. (2018). The effects of tai chi on heart rate variability in older Chinese individuals with depression. *International Journal of Environmental Research and Public Health*.

[60] Liao S., Chong M., Tan M., Chua Y. (2019). Tai Chi with music improves quality of life among community-dwelling older persons with mild to moderate depressive symptoms: a cluster randomized controlled trial. *Geriatric Nursing*.

[61] Lavretsky H., Alstein L. L., Olmstead R. E. (2011). Complementary use of Tai Chi Chih augments escitalopram treatment of geriatric depression: a randomized controlled trial. *The American Journal of Geriatric Psychiatry*.

[62] Liu X., Vitetta L., Kostner K. (2015). The effects of tai chi in centrally obese adults with depression symptoms. *Evidence-Based Complementary and Alternative Medicine*.

[63] Lam L. C. W., Chau R. C. M., Wong B. M. L. (2012). A 1-year randomized controlled trial comparing mind body exercise (Tai Chi) with stretching and toning exercise on cognitive function in older Chinese adults at risk of cognitive decline. *Journal of the American Medical Directors Association*.

[64] Kasai J. Y. T., Busse A. L., Magaldi R. M. (2010). Effects of Tai Chi Chuan on cognition of elderly women with mild cognitive impairment. *Einstein (São Paulo)*.

[65] Tsai P.-F., Chang J. Y., Beck C., Kuo Y.-F., Keefe F. J., Rosengren K. (2015). A supplemental report to a randomized cluster trial of a 20-week Sun-style Tai Chi for osteoarthritic knee pain in elders with cognitive impairment. *Complementary Therapies in Medicine*.

[66] Sungkarat S., Boripuntakul S., Chattipakorn N., Watcharasaksilp K., Lord S. R. (2017). Effects of tai chi on cognition and fall risk in older adults with mild cognitive impairment: a randomized controlled trial. *Journal of the American Geriatrics Society*.

[67] Ho R. T. H., Fong T. C. T., Wan A. H. Y. (2016). A randomized controlled trial on the psychophysiological effects of physical exercise and Tai-Chi in patients with chronic schizophrenia. *Schizophrenia Research*.

[68] Lan C., Chen S.-Y., Lai J.-S., Wong A. M.-K. (2013). Tai chi chuan in medicine and health promotion. *Evidence-Based Complementary and Alternative Medicine*.

[69] Li F., Harmer P., Fitzgerald K. (2012). Tai chi and postural stability in patients with Parkinson’s disease. *New England Journal of Medicine*.

[70] Hackney M. E., Earhart G. M. (2008). Tai Chi improves balance and mobility in people with Parkinson disease. *Gait & Posture*.

[71] Li F., Harmer P., Liu Y. (2014). A randomized controlled trial of patient-reported outcomes with Tai Chi exercise in Parkinson’s disease. *Movement Disorders*.

[72] Cheon S.-M., Chae B.-K., Sung H.-R., Lee G. C., Kim J. W. (2013). The efficacy of exercise programs for Parkinson’s disease: Tai Chi versus combined exercise. *Journal of Clinical Neurology*.

[73] Choi H.-J. (2016). Effects of therapeutic Tai Chi on functional fitness and activities of daily living in patients with Parkinson disease. *Journal of Exercise Rehabilitation*.

[74] Choi H.-J., Garber C. E., Jun T.-W., Jin Y.-S., Chung S.-J., Kang H.-J. (2013). Therapeutic effects of tai chi in patients with Parkinson’s disease. *ISRN Neurology*.

[75] Nyman S. R., Ingram W., Sanders J. (2019). Randomised controlled trial of the effect of tai chi on postural balance of people with dementia. *Clinical Interventions in Aging*.

[76] Li F., Fisher K. J., Harmer P., Irbe D., Tearse R. G., Weimer C. (2004). Tai Chi and self-rated quality of sleep and daytime sleepiness in older adults: a randomized controlled trial. *Journal of the American Geriatrics Society*.

[77] Zhu X.-D., Lei X.-P., Dong W.-B. (2017). Resveratrol as a potential therapeutic drug for respiratory system diseases. *Drug Design, Development and Therapy*.

[78] Lozano R., Naghavi M., Foreman K. (2012). Global and regional mortality from 235 causes of death for 20 age groups in 1990 and 2010: a systematic analysis for the global burden of disease study 2010. *Lancet (London, England)*.

[79] O’Donnell D. E., Gebke K. B. (2014). Examining the role of activity, exercise, and pharmacology in mild COPD. *Postgraduate Medicine*.

[80] Zhu S., Shi K., Yan J. (2018). A modified 6-form Tai Chi for patients with COPD. *Complementary Therapies in Medicine*.

[81] Polkey M. I., Qiu Z.-H., Zhou L. (2018). Tai chi and pulmonary rehabilitation compared for treatment-naive patients with COPD. *Chest*.

[82] Yeh G. Y., Roberts D. H., Wayne P. M., Davis R. B., Quilty M. T., Phillips R. S. (2010). Tai chi exercise for patients with chronic obstructive pulmonary disease: a pilot study. *Respiratory Care*.

[83] Leung R. W. M., McKeough Z. J., Peters M. J., Alison J. A. (2013). Short-form Sun-style T’ai Chi as an exercise training modality in people with COPD. *European Respiratory Journal*.

[84] Guariguata L. (2012). By the numbers: new estimates from the IDF diabetes atlas update for 2012. *Diabetes Research and Clinical Practice*.

[85] Whiting D. R., Guariguata L., Weil C., Shaw J. (2011). IDF diabetes atlas: global estimates of the prevalence of diabetes for 2011 and 2030. *Diabetes Research and Clinical Practice*.

[86] Chatterjee S., Khunti K., Davies M. J. (2017). Type 2 diabetes. *The Lancet*.

[87] Dumas M. E., Kinross J., Nicholson J. K. (2014). Metabolic phenotyping and systems biology approaches to understanding metabolic syndrome and fatty liver disease. *Gastroenterology*.

[88] Zhang Y., Fu F. H. (2008). Effects of 14-week Tai Ji Quan exercise on metabolic control in women with type 2 diabetes. *The American Journal of Chinese Medicine*.

[89] Liu X., Miller Y. D., Burton N. W., Chang J.-H., Brown W. J. (2013). The effect of Tai Chi on health-related quality of life in people with elevated blood glucose or diabetes: a randomized controlled trial. *Quality of Life Research*.

[90] Tsang T., Orr R., Lam P., Comino E., Singh M. F. (2008). Effects of Tai Chi on glucose homeostasis and insulin sensitivity in older adults with type 2 diabetes: a randomised double-blind sham-exercise-controlled trial. *Age and Ageing*.

[91] Tsang T., Orr R., Lam P., Comino E. J., Singh M. F. (2007). Health benefits of Tai Chi for older patients with type 2 diabetes: the “move it for diabetes study”--a randomized controlled trial. *Clinical Interventions in Aging*.

[92] Choi Y.-S., Song R., Ku B. J. (2017). Effects of a T’ai Chi-based health promotion program on metabolic syndrome markers, health behaviors, and quality of life in middle-aged male office workers: a randomized trial. *The Journal of Alternative and Complementary Medicine*.

[93] Siegel R. L., Miller K. D., Jemal A. (2020). Cancer statistics, 2020. *CA: A Cancer Journal for Clinicians*.

[94] Friedenreich C. M., Orenstein M. R. (2002). Physical activity and cancer prevention: etiologic evidence and biological mechanisms. *The Journal of Nutrition*.

[95] Mustian K. M., Palesh O. G., Flecksteiner S. A. (2008). Tai Chi Chuan for breast cancer survivors. *Medicine and Sport Science*.

[96] Ni X., Chan R. J., Yates P., Hu W., Huang X., Lou Y. (2019). The effects of Tai Chi on quality of life of cancer survivors: a systematic review and meta-analysis. *Supportive Care in Cancer*.

[97] Zeng Y., Luo T., Xie H., Huang M., Cheng A. S. K. (2014). Health benefits of qigong or tai chi for cancer patients: a systematic review and meta-analyses. *Complementary Therapies in Medicine*.

[98] Zhang Y.-J., Wang R., Chen P.-J., Yu D.-H. (2013). Effects of Tai Chi Chuan training on cellular immunity in post-surgical non-small cell lung cancer survivors: a randomized pilot trial. *Journal of Sport and Health Science*.

[99] Zhang L.-L., Wang S.-Z., Chen H.-L., Yuan A.-Z. (2016). Tai chi exercise for cancer-related fatigue in patients with lung cancer undergoing chemotherapy: a randomized controlled trial. *Journal of Pain and Symptom Management*.

[100] Campo R. A., O’Connor K., Light K. C. (2013). Feasibility and acceptability of a Tai Chi Chih randomized controlled trial in senior female cancer survivors. *Integrative Cancer Therapies*.

[101] Chen E. W., Fu A. S. N., Chan K. M., Tsang W. W. N. (2012). The effects of Tai Chi on the balance control of elderly persons with visual impairment: a randomised clinical trial. *Age and Ageing*.

[102] Gemmell C., Leathem J. M. (2006). A study investigating the effects of Tai Chi Chuan: individuals with traumatic brain injury compared to controls. *Brain Injury*.

[103] Jung S., Lee E.-N., Lee S.-R., Kim M.-S., Lee M. S. (2012). Tai chi for lower urinary tract symptoms and quality of life in elderly patients with benign prostate hypertrophy: a randomized controlled trial. *Evidence-Based Complementary and Alternative Medicine*.

[104] Nickens H. (1985). Intrinsic factors in falling among the elderly. *Archives of Internal Medicine*.

[105] Perry B. C. (1982). Falls among the elderly. *Journal of the American Geriatrics Society*.

[106] Lord S. R., Ward J. A., Williams P., Anstey K. J. (1993). An epidemiological study of falls in older community-dwelling women: the Randwick falls and fractures study. *Australian Journal of Public Health*.

[107] Rubenstein L. Z. (2006). Falls in older people: epidemiology, risk factors and strategies for prevention. *Age and Ageing*.

[108] Hong Y., Li J. X., Robinson P. D. (2000). Balance control, flexibility, and cardiorespiratory fitness among older Tai Chi practitioners. *British Journal of Sports Medicine*.

[109] Zhou J., Chang S., Cong Y. (2015). Effects of 24 weeks of tai chi exercise on postural control among elderly women. *Research in Sports Medicine*.

[110] Li Y., Devault C. N., Van Oteghen S. (2007). Effects of extended Tai Chi intervention on balance and selected motor functions of the elderly. *The American Journal of Chinese Medicine*.

[111] Sun W., Ma X., Wang L. (2019). Effects of tai chi chuan and brisk walking exercise on balance ability in elderly women: a randomized controlled trial. *Motor Control*.

[112] Li F., Harmer P., Fisher K. J. (2005). Tai Chi and fall reductions in older adults: a randomized controlled trial. *The Journals of Gerontology Series A: Biological Sciences and Medical Sciences*.

[113] Hosseini L., Kargozar E., Sharifi F., Negarandeh R., Memari A.-H., Navab E. (2018). Tai Chi Chuan can improve balance and reduce fear of falling in community dwelling older adults: a randomized control trial. *Journal of Exercise Rehabilitation*.

[114] Li F., Harmer P., Fisher K. J., Mcauley E. (2004). Tai Chi: improving functional balance and predicting subsequent falls in older persons. *Medicine & Science in Sports & Exercise*.

[115] Zhang J.-G., Ishikawa-Takata K., Yamazaki H., Morita T., Ohta T. (2006). The effects of Tai Chi Chuan on physiological function and fear of falling in the less robust elderly: an intervention study for preventing falls. *Archives of Gerontology and Geriatrics*.

[116] Li F., Fisher K. J., Harmer P., McAuley E. (2005). Falls self-efficacy as a mediator of fear of falling in an exercise intervention for older adults. *The Journals of Gerontology: Series B*.

[117] Li F., Harmer P., McAuley E., Fisher K. J., Duncan T. E., Duncan S. C. (2001). Tai Chi, self-efficacy, and physical function in the elderly. *Prevention Science*.

[118] Tajik A., Rejeh N., Heravi-Karimooi M. (2018). The effect of Tai Chi on quality of life in male older people: a randomized controlled clinical trial. *Complementary Therapies in Clinical Practice*.

[119] Zheng S., Kim C., Lal S., Meier P., Sibbritt D., Zaslawski C. (2018). The effects of twelve weeks of tai chi practice on anxiety in stressed but healthy people compared to exercise and wait-list groups-A randomized controlled trial. *Journal of Clinical Psychology*.

[120] Yang Y., Verkuilen J. V., Rosengren K. S., Grubisich S. A., Reed M. R., Hsiao-Wecksler E. T. (2007). Effect of combined Taiji and Qigong training on balance mechanisms: a randomized controlled trial of older adults. *Medical Science Monitor*.

[121] Christou E. A., Yang Y., Rosengren K. S. (2003). Rapid communication. Taiji training improves knee extensor strength and force control in older adults. *The Journals of Gerontology Series A: Biological Sciences and Medical Sciences*.

[122] Yang Y., Verkuilen J., Rosengren K. S. (2008). Effects of a traditional Taiji/Qigong curriculum on older adults’ immune response to influenza vaccine. *Medicine and Sport Science*.

[123] Yang G.-Y., Wang L.-Q., Ren J. (2015). Evidence base of clinical studies on Tai Chi: a bibliometric analysis. *PLoS One*.

[124] Garber C. E., Blissmer B., Deschenes M. R. (2011). Quantity and quality of exercise for developing and maintaining cardiorespiratory, musculoskeletal, and neuromotor fitness in apparently healthy adults. *Medicine & Science in Sports & Exercise*.

[125] Taylor-Piliae R. E. (2008). The effectiveness of Tai Chi exercise in improving aerobic capacity: an updated meta-analysis. *Medicine and Sport Science*.

[126] Li J. X., Xu D. Q., Hong Y. (2009). Changes in muscle strength, endurance, and reaction of the lower extremities with Tai Chi intervention. *Journal of Biomechanics*.

[127] Wong A. M., Lin Y.-C., Chou S.-W., Tang F.-T., Wong P.-Y. (2001). Coordination exercise and postural stability in elderly people: effect of Tai Chi Chuan. *Archives of Physical Medicine and Rehabilitation*.

[128] Yildirim P., Ofluoglu D., Aydogan S., Akyuz G. (2016). Tai Chi vs. combined exercise prescription: a comparison of their effects on factors related to falls. *Journal of Back and Musculoskeletal Rehabilitation*.

[129] Bu B., Hou L. R., Zhou X. L. (2010). Research progress on exercise prescription: a systematic review. *Chinese Journal of Evidence-Based Medicine*.

[130] Haskell W. L., Lee I. M., Pate R. R. (2007). Physical activity and public health: updated recommendation for adults from the American college of sports medicine and the American heart association. *Circulation*.

[131] Young D. R., Appel L. J., Jee S., Miller E. R. (1999). The effects of aerobic exercise and T’ai Chi on blood pressure in older people: results of a randomized trial. *Journal of the American Geriatrics Society*.

[132] Li F., Harmer P., Fitzgerald K. (2018). Effectiveness of a therapeutic Tai Ji Quan intervention vs a multimodal exercise intervention to prevent falls among older adults at high risk of falling. *JAMA Internal Medicine*.

[133] Tsai J.-C., Wang W.-H., Chan P. (2003). The beneficial effects of Tai Chi Chuan on blood pressure and lipid profile and anxiety status in a randomized controlled trial. *The Journal of Alternative and Complementary Medicine*.

[134] World Health Organization (2020). *Fact Sheet on Noncommunicable Diseases*.

[135] Ding D., Lawson K. D., Kolbe-Alexander T. L. (2016). The economic burden of physical inactivity: a global analysis of major non-communicable diseases. *The Lancet*.

[136] Pratt M., Ramirez Varela A., Salvo D., Kohl III H. W., Ding D. (2020). Attacking the pandemic of physical inactivity: what is holding us back?. *British Journal of Sports Medicine*.

[137] Wayne P. M., Berkowitz D. L., Litrownik D. E., Buring J. E., Yeh G. Y. (2014). What do we really know about the safety of Tai Chi?: a systematic review of adverse event reports in randomized trials. *Archives of Physical Medicine and Rehabilitation*.

