# Supplementary material for: Clinical Evidence of Tai Chi Exercise Prescriptions: A Systematic Review
Source: Evid Based Complement Alternat Med. 2021 Mar 10;2021:5558805. doi: 10.1155/2021/5558805 (PMC7972853; doi:10.1155/2021/5558805)
Supplement: Supplementary Materials — Table S1: basic characteristics of the included studies. Table S2: musculoskeletal system or connective tissue diseases. Table S3: circulatory system diseases. Table S4: mental and behavioral disorders. Table S5: nervous system diseases. Table S6: respiratory system diseases. Table S7: endocrine, nutritional, or metabolic diseases. Table S8: neoplasms. Table S9: other disease conditions. Table S10: healthy populations. Figure S1: risk of bias summary. [file 5558805.f1.zip › 5558805.f1/Table S1 Basic Characteristics of the Included Studies.pdf]

Table S1. Basic Characteristics of the Included Studies (n=139).

First author	Study design	Participants	Intervention	Control	Duration/Frequency/Time	Outcomes
Tsai (2013)[1]	RCT	Elders with knee OA	12-form Sun-style TC	Health education	20 weeks; three times a week; 40min per session	WOMAC; GUG; STS; MMSE
Song (2010)[2]	RCT	Older women with OA	31-form Sun-style TC；Breathing exercise	Self-help education	24 weeks; once a week; 60min per session	Knee muscle strength and endurance; BMD; fear of falling
Wang (2016)[3]	RCT	Knee OA patients	Classical Yang-style TC	Physical therapy	12 weeks; twice a week; 60min per session	WOMAC; physical function; depression; medication use; quality of life
Lu (2017)[4]	RCT	Older women with OA	Simplified 24-form TC (8 movements)	Health education	24 weeks; three times a week; 60min per session	PSQI; SF-36; TUG; BBS
Song (2007)[5]	RCT	Older women with OA	12-form Sun-style TC	Control group	12 weeks; three times a week (1-2 weeks), once a week (3-12weeks); 60min per session	Motivation Scale; Health Behavior Scale; WOMAC
Wortley (2013)[6]	RCT	Knee OA patients	12-form Yang-style TC	C1: Resistance training; C2: Control group	10 weeks; twice a week; 60min per session	6MWT; TUG; SCD; WOMAC
Song (2003)[7]	RCT	Older women with OA	12-form Sun-style TC	Control group	12 weeks; three times a week (1-2 weeks), once a week (3-12weeks); 60min per session	WOMAC; Physical symptoms; Balance function; Cardiovascular function
Hartman (2000)[8]	RCT	Older adults with OA	9-form Yang-style TC	Usual physical activities	12 weeks; twice a week; 60min per session	ASES; AIMS; Functional Mobility
Zhu (2016)[9]	RCT	Older women with OA	Simplified 24-form TC (8 movements)	Health education	24 weeks; three times a week; 60min per session	Gait velocity; WOMAC; SPPB; Step length

Callahan (2016)[10]	RCT	Knee OA patients	12-form Sun-style TC	Usual care and activities	8 weeks; twice a week; 60min per session	VAS; Physical function
Brismee (2007)[11]	RCT	Knee OA patients	Simplified 24-form TC	Health education	12 weeks; three times a week; 40min per session;	VAS; Physical function; WOMAC
Schmid (2013)[12]	RCT	Knee OA patients	10-form Yang-style TC+10min Breathing exercise	C1: Health education; C2: Stretching exercise	12 weeks; twice a week; 60min per session	Knee joint proprioception
Fransen (2007)[13]	RCT	Elders with knee OA	24-form Sun-style TC	C1: Hydrotherapy class C2: Control group	12 weeks; twice a week; 60min per session	SF-12; WOMAC; Psychological well-being; Physical performance
Ni (2010)[14]	RCT	Older women with OA	Simplified 24-form TC	C1: Health education; C2: Stretching exercise	24 weeks; two to four times a week; 40min per session	WOMAC; Physical function; Physical performance; Knee pain
Wang (2009)[15]	RCT	Knee OA patients	10-form Yang-style TC+10min Breathing exercise	C1: Health education; C2: Stretching exercise	12 weeks; twice a week; 60min per session	WOMAC; VAS; SF-36
Wu (2013)[16]	RCT	Retired athlete with NLBP	Simplified 24-form TC	C1: Swimming; C2: Jogging; C3: Backward walking; C4: No exercise	24 weeks; five times a week; 45min per session	VAS; BMI; Heart rate; Blood pressure
Cho (2014)[17]	RCT	Young males with acute LBP	7-form TC	Stretching exercise	4 weeks; three times a week; 60min per session	sEMG; VAS
Hall (2011)[18]	RCT	patients with NLBP	21-form Sun-style TC	Usual health care	10 weeks; 40min per session; twice a week (1-8 weeks); once a week (9-10 weeks)	NRS; quality of life; emotional and cognitive level
Zou (2019)[19]	RCT	Elders with NLBP	Chen-style TC	C1: Core stabilization training; C2: Control group	12 weeks; three times a week; 60min per session	VAS; Neuromuscular function
Jones (2012)[20]	RCT	Patients with FM	8-form Yang-style TC	Education intervention	12 weeks; twice a week; 90min per session	FIQ; FIQ-PF; PSQI

Wang (2010)[21]	RCT	Patients with FM	10-form Yang-style TC	C1: Health education; C2: Stretching exercise	12 weeks; twice a week; 60min per session	FIQ; PSQI; VAS; SF-36
Wang (2018)[22]	RCT	Patients with FM	Classical Yang-style TC	Aerobic exercise	12/24 weeks; once-twice a week; 60min per session	FIQ; SF-36; HADS
Wong (2018)[23]	RCT	Patients with FM	10-form Yang-style TC	Regular lifestyle habits	12 weeks; three times a week; 55min per session	VAS; HRV; SRS
Maddali (2016)[24]	RCT	Patients with FM	14-form TC	Health education	16 weeks; twice a week; 60min per session	PSQI; SF-36; HADS; HAQ
Maciaszek (2007)[25]	RCT	Men with osteoporosis	Simplified 24-form TC (5 movements)	Control group	18 weeks; twice a week; 45min per session	Physical balance
Chyu (2010)[26]	RCT	Women with osteoporosis	Simplified 24-form TC	Control group	24 weeks; three times a week; 60min per session	Gait; Physical function; SF-36
Wayne (2012)[27]	RCT	Women with osteoporosis	TC+Usual care	Usual care	36 weeks; 60min per session; twice a week (1-4 weeks); once a week (5-36 weeks)	BMD; SF-36
Lauche (2017)[28]	RCT	Patients chronic non-specific neck pain	13-form Yang-style TC	C1: Control group; C2: Neck exercise	12 weeks; once a week; 60-90min per session	HADS; PSS; VAS
Lauche (2016)[29]	RCT	Patients chronic non-specific neck pain	13-form Yang-style TC	C1: Control group; C2: Neck exercise	12 weeks; once a week; 75-90min per session	VAS; SF-36; NDI
You (2018)[30]	RCT	Older adults with chronic multisite pain	8-formYang-style TC	Light physical exercise	12 weeks; twice a week; 60min per session	Pain characteristics; cognition; physical function; gait mobility
Wang (2008)[31]	RCT	Adults with rheumatoid arthritis	Yang-style TC+10min Breathing exercise	C1: Stretching exercise; C2: Health education	12 weeks; twice a week; 60min per session	HAQ; VAS; SF-36
Buyukturan (2019)[32]	RCT	Patients with partial ACL injuries	10 form Yang-style TC	Control group	24 weeks; three times a week; 60min per session	VAS; LKS
Lee (2008)[33]	RCT	Patients with ankylosing spondylitis	21 form Sun-style TC; Usual care	Usual care	8 weeks; twice a week; 45min per session	Disease Activity; FFD; Depression

Taylor-P (2014)[34]	RCT	Older stroke survivors	Simplified 24-form TC	C1: SilverSneakers; C2: Usual care	12 weeks; three times a week; 60min per session	Physical function; Fall rate; SF-36
Chan (2017)[35]	RCT	Stroke survivors	12 form Yang-style TC	C1: Conventional exercise; C2: Control group	12 weeks; twice a week; 60min per session	Single Cognitive Task; Single Physical Task; Dual-Tasking
Xie (2018)[36]	RCT	Stroke patients	TC (Yunshou movement)	Balance rehabilitation training	12 weeks; five times a week; 60min per session	BBS; MFES; SF-36; BDI
Kim (2015)[37]	RCT	Stroke patients	10 form Yang-style TC+General physical therapy	General physical therapy	6 weeks; twice a week; 60min per session	Static balance; Gait ability; SF-36
Au-Yeung (2009)[38]	RCT	Stroke patients	12 form Sun-style TC	Stretching exercise	12 weeks; once a week; 60min per session	Dynamic standing balance in the Limit of Stability test; Standing equilibrium
Wang(A) (2010)[39]	RCT	Stroke patients	Yang-style TC	Rehabilitation program	12 weeks; once a week; 50min per session	GHQ; PSQI
Redwine (2012)[40]	RCT	Patients with HF	Yang-style TC	Usual care	12 weeks; twice a week; 60min per session	BDI-t; BDI-t; BDI-c
Barrow (2007)[41]	RCT	Patients with HF	Wu-style TC+Qigong	C1: Medical supervision; C2: Drug treatment	16 weeks; twice a week; 55min per session	Shuttle walk test; symptom scores; quality of life indices
Yeh (2004)[42]	RCT	Patients with HF	5-form Yang-style TC+Usual care	Usual care	12 weeks; twice a week; 60min per session	Quality of life; exercise capacity; serumB-type natriuretic peptide
Yeh (2008)[43]	RCT	Patients with HF	5-form Yang-style TC+Usual care	Usual care	12 weeks; twice a week; 60min per session	Quality of life; exercise capacity
Yeh(A) (2008)[44]	RCT	Patients with HF	5-form Yang-style TC+Usual care	Usual care	12 weeks; twice a week; 60min per session	Quality of life; exercise capacity; B-type natriuretic peptide
Yeh (2011)[45]	RCT	Patients with HF	5-form Yang-style TC	Health education	12 weeks; twice a week; 60min per session	Quality of life; exercise capacity; Psychosocial Functioning

Yeh (2013)[46]	RCT	Patients with HF	5-form Yang-style TC	Aerobic exercise	12 weeks; twice a week; 60min per session	Quality of life; exercise capacity; Psychosocial Functioning
Shou (2019)[47]	RCT	Hypertension in young and middle-aged in-service staff	Simplified 24-form TC	Usual lifestyle	12 weeks; seven times a week; 40-90min per session	BMI; blood pressure; physical index; quality of life
Chan (2018)[48]	RCT	Adults with hypertension	Simplified 24-form TC	C1: Brisk Walking; C2: Control group	12 weeks; twice a week; 60min per session	Blood pressure; fasting blood sugar; glycated haemoglobin; BMI
Tsai (2003)[49]	RCT	Patients with hypertension	108-form Yang-style TC	Usual lifestyle	12 weeks; three times a week; 50min per session	Blood pressure; lipid profile; anxiety status
Ma (2018)[50]	RCT	Older adults with hypertension	Simplified 24-form TC+ Usual care	Usual care	24 weeks; three-five times a week; 60min per session	SSRS; SF-36
Sato (2010)[51]	RCT	Patients With CHD	8-form Yang-style TC+Rehabilitation training	Rehabilitation training	48 weeks; once a week; 60min per session	BRS; HRV
Chang (2010)[52]	RCT	Patients With CHD	108-form Yang-style TC+Usual care	Usual care	24 weeks; three times a week; 115min per session	Exercise Test Responses; Blood pressure; Heart rate
Liu (2010)[53]	RCT	Patients With CHD	12-form TC+Usual care	Usual care	12 weeks; twice a week; 60min per session	leg strength; leg flexibility; stationary balance; agility; aerobic endurance
Salmoirago (2017)[54]	RCT	Patients With CHD	TC	Usual care	12 weeks; twice a week; 60min per session	PA Readiness questionnaire; quality of life
Nery (2015)[55]	RCT	Patients with myocardial infarction	Simplified 24-form TC (5 movements)	Stretching exercise	12 weeks; three times a week; 60min per session	VO_2peak; CPET
Li (2014)[56]	RCT	Patients with PD	6-form Yang-style TC	C1: Resistance training; C2: Stretching exercise	24 weeks; twice a week; 60min per session	PDQ; VPS; UPDRS; 50-foot speed walk
Choi (2016)[57]	RCT	Patients with PD	10-form TC+10min Meditation	Control group	12 weeks; three times a week; 60min per session	6MWT; UPDRS

Li (2012)[58]	RCT	Patients with PD	6-form Yang-style TC	C1: Resistance training; C2: Stretching exercise	24 weeks; twice a week; 60min per session	limits-of-stability test; gait and strength; scores on functional-reach; UPDRS
Hackney (2008)[59]	RCT	Patients with PD	Yang-style TC	Control group	13 weeks; twice a week; 60min per session	UPDRS; BBS; TUG
Cheon (2013)[60]	RCT	Patients with PD	12-form Sun-style TC	C1: Combined exercise; C2: Control group	8 weeks; three times a week; 60min per session	UPDRS; PD QoL scale
Choi (2013)[61]	RCT	Patients with PD	10-form TC+10min Meditation	Control group	12 weeks; three times a week; 60min per session	UPDRS; TUG; 6MWT
Li (2004)[62]	RCT	Older adults with sleep disorder	8-form Yang-style TC	Low-impact exercise	24 weeks; three times a week; 60min per session	PSQI; ESS; physical performance; SF-12
Liu (2018)[63]	RCT	Patients with dementia	10-form Yang-style TC	Usual lifestyle	16 weeks; twice a week; 60min per session	TUG; TCS
Nyman (2019)[64]	RCT	Patients with dementia	5-form Chen-style TC; Usual care	Usual care	20 weeks; twice a week; 45min per session	TUG; BBS
Abbott (2007)[65]	RCT	Patients tension-type headaches	Simplified 24-form TC	Control group	15 weeks; twice a week; 60min per session	SF-36; HIT
Zhu (2018)[67]	RCT	People with COPD	Simplified 24-form TC (6 movements)	Control group	12 weeks; three times a week; 40-50min per session	Lung function; exercise capacity; dyspnea symptom; health status
Ng, L (2014)[67]	RCT	People with COPD	5-form Sun-style TC+ Rehabilitation training	Rehabilitation training	6 weeks; twice a week; 80min per session	COPD-CSES; SEMSOB; SGRQ-HKC
Leung (2013)[68]	RCT	People with COPD	21-form Sun-style TC	Usual care	12 weeks; twice a week; 60min per session	Exercise capacity; Physical performance; Balance
Polkey (2018)[69]	RCT	People with COPD	Simplified 24-form TC	Pulmonary Rehabilitation	12 weeks; five times a week; 60min per session	SGRQ; mMRC
Yeh (2010)[70]	RCT	People with COPD	5-form Yang-style TC; Usual care	Usual care	12 weeks; twice a week; 60min per session	Exercise Capacity; Functional Status; CRQ; Pulmonary Function Tests

Niu (2014)[71]	RCT	People with COPD	TC; Usual care	Usual care	24 weeks; four times a week; 50min per session	Lung function parameters; blood gas parameters; 6MWD;
Zhang (2018)[72]	RCT	Subthreshold depression adolescents	Simplified 24-form TC+Mindfulness	Normal PE classes	8 weeks; twice a week; 90min per session	PHQ-9; CPSS; MAAS
Lavretsky (2011)[74]	RCT	Older adults with major depression	TC+Medication treatment	C1: Health education; C2: Medication treatment	10 weeks; once a week; 120min per session	Depression; cognition; physical function; inflammation
Yeung (2012)[74]	RCT	Chinese Americans with major depression	108-form Yang-style TC	Control group	12 weeks; twice a week; 60min per session	Quality of life; MSPSS
Liao (2019)[75]	RCT	Older persons with mild to moderate depression	Simplified 24-form TC	Health education	12 weeks; three times a week; 50min per session	Quality of life
Liu (2015)[76]	RCT	Obese adults with depression	TC; Usual care	Usual care	12 weeks; three times a week; 60-90min per session	DASS21
Liu(A) (2018)[77]	RCT	Older individuals with depression	Simplified 24-form TC; 42-form TC	Control group	24 weeks; three times a week; 60min per session	GDS; Heart Rate
Ho (2016)[78]	RCT	Patients with chronic schizophrenia	22-form Wu-style TC	C1: Exercise group; C2: Control group	12 weeks; three times a week; 60min per session	PANSS; ADL; PSS
Zhang (2008)[79]	RCT	Patients with type 2 diabetes	Simplified 24-form TC	Control group	14 weeks; five times a week; 60min per session	Blood pressures; FPG; FPI; Heart rate
Tsang (2008)[80]	RCT	Patients with type 2 diabetes	TC (12-form Sun-style; Yang-style)	Stretching exercise	16 weeks; twice a week; 60min per session	HOMA2-IR; Habitual physical activity
Tsang (2007)[81]	RCT	Patients with type 2 diabetes	TC (12-form Sun-style; Yang-style)	Stretching exercise	16 weeks; twice a week; 60min per session	Body composition; nutritional status; Physical function; Muscle performance
Liu (2013)[82]	RCT	Patients with type 2 diabetes	KaiMai-style TC	Usual medical care	12 weeks; three times a week; 90min per session	SF-36
Choi (2017)[83]	RCT	Middle-aged male office workers with metabolic syndrome	TC (Sun-style; Yang-style); Health education	Health education	12 weeks; twice a week; 50min per session	Blood; health behaviors; quality of life

Zhang (2013)[84]	RCT	Lung cancer survivors	Simplified 24-form TC	Control group	16 weeks; three times a week; 60min per session	hemoglobin; total lymphocyte counts
Campo (2013)[85]	RCT	Senior female cancer survivors	19-form TC; Health education	Health education	12 weeks; three times a week; 60min per session	SF-36
Zhang (2016)[86]	RCT	Patients with lung cancer	8-form Yang-style TC	Low-impact exercise	12 weeks; four times a week; 60min per session	MFSI-SF
Jung (2012)[87]	RCT	Patients with benign prostate hypertrophy	20-form TC	Control group	12 weeks; three times a week; 60min per session	IPSS; LUTSs; Quality of life
Lam (2012)[88]	RCT	Elders with mild cognitive impairment	Simplified 24-form TC	Stretching exercise	48 weeks; three times a week; 30min per session	BBS; Cognitive test scores; CSDD
Tsai (2015)[89]	RCT	Elders with mild cognitive impairment	12-form Sun-style TC	Health education	20 weeks; three times a week; 40min per session	VDS; Pain behavior
Kasai (2010)[90]	RCT	Elders with mild cognitive impairment	Yang-style TC	Control group	24 weeks; twice a week; 60min per session	SMC; RBMT; WAIS
Sungkarat (2017)[91]	RCT	Elders with mild cognitive impairment	10-form TC	Health education	15 weeks; three times a week; 50min per session	PPA; Cognitive performance
Maciaszek (2012)[92]	RCT	Elderly men with dizziness	Simplified 24-form TC (5 movements)	Control group	18 weeks; twice a week; 45min per session	Body balance
Kong (2019)[93]	RCT	Children and adolescents with intellectual disability	8-form Yang-style TC	C1: Aerobic exercise； C2: Control group	12 weeks; twice a week; 60min per session	Components of Physical Fitness
Chen (2012)[94]	RCT	Elderly persons with visual impairment	8-form Yang-style TC	Control group	16 weeks; three times a week; 90min per session	Sensory organization test; Muscular strength;
Gemmell, (2006)[95]	RCT	Individuals with traumatic brain injury	Chen-style TC	Control group	6 weeks; three times a week; 45min per session	SF-36; RSES; VAMS
Voukelatos (2007)[96]	RCT	Older adults	TC (Sun-style; Yang-style)	Control group	16 weeks; once a week; 60min per session	Fall rate; Balance test
Yang (2007)[97]	RCT	Older adults	7-form Chen-style TC+Qigong	Control group	24 weeks; three times a week; 60min per session	Sensory organization test; postural stability
Li (2018)[98]	RCT	Older adults	8-form TC	C1: Multimodal exercise C2: Stretching exercise	24 weeks; twice a week; 60min per session	Fall rate; Physical performance

Zhou (2015)[99]	RCT	Elderly women	Simplified 24-form TC	Control group	24 weeks; four times a week; 60min per session	Balance
Lelard (2010)[100]	RCT	Older adults	10-form TC	Balance training	12 weeks; twice a week; 30min per session	Static postural control; Walking speed
Li, Y (2007)[101]	RCT	Older adults	Simplified 24-form TC	Control group	12 weeks; once a week; 60min per session	Ankle Flexibility; Balance; Muscular strength
Hwang (2016)[102]	RCT	Older adults	18-form Yang-style TC	Lower extremity training	24 weeks; once a week; 60min per session	handgrip strength; balance; depression; cognitive function
Sun (2019)[103]	RCT	Elderly women	Simplified 24-form TC	Brisk Walking; Health education	16 weeks; five times a week; 60min per session	Balance tests
Kim (2009)[104]	RCT	Older adults	12-form TC	Health education	12 weeks; three times a week; 60min per session	Balance ability
Li(A) (2005)[105]	RCT	Older adults	Simplified 24-form TC	Stretching exercise	24 weeks; three times a week; 60min per session	Fall counts; Functional balance; Physical performance
Hosseini (2018)[106]	RCT	Older adults	Simplified 24-form TC	Control group	8 weeks; twice a week; 55min per session	Balance; Fear of fall
Li(A) (2004)[107]	RCT	Older adults	Simplified 24-form TC	Stretching exercise	24 weeks; three times a week; 60min per session	Functional balance; Fall counts
Mortazavi (2018)[108]	RCT	Older adults	Yang-style TC	Usual activities	10 weeks; three times a week; 60min per session	BBS; FES-I
Zhang (2006)[109]	RCT	Older adults	Simplified 24-form TC	Control group	8 weeks; seven times a week; 60min per session	Physical performance
Nguyen (2012)[110]	RCT	Older adults	Simplified 24-form TC	Usual activities	24 weeks; twice a week; 60min per session	FES; PSQI
Chan (2004)[111]	RCT	Postmenopausal women	Yang-style TC	Usual activities	48 weeks; five times a week; 45min per session	BMD
Wayne (2013)[112]	RCT	Older adults	TC	Usual care	24 weeks; twice a week; 30min per session	Heart rate; Postural control; Gait measures

Li(A) (2001)[113]	RCT	Older adults	Simplified 24-form TC	Usual activities	24 weeks; twice a week; 60min per session	Short-Form General Health Survey
Schitter (2016)[114]	RCT	Healthy people	18-form Yang-style TC	Control group	12 weeks; twice a week; 60min per session	Depression; Physical well-being
Shen (2007)[115]	RCT	Older adults	Simplified 24-form TC	Resistance training	24 weeks; three times a week; 40min per session	bone-specific alkaline phosphatase; parathyroid hormone
Li (2002)[116]	RCT	Older adults	TC	Usual activities	24 weeks; twice a week; 60min per session	Physical function; Quality of life
Sun (2018)[117]	RCT	Elderly women	Simplified 24-form TC	C1: Brisk Walking; C2: Health education	16 weeks; five times a week; 60min per session	Posture control ability; Balance
Sun (2016)[118]	RCT	Elderly women	Simplified 24-form TC	Control group	48 weeks; three times a week; 60min per session	Neuromuscular reaction time
Zou (2017)[119]	RCT	Elderly women	Simplified 24-form TC	Usual activities	8 weeks; three times a week; 90min per session	Gait parameters; Low-limb flexibility
Li (2008)[120]	RCT	Older adults	Simplified 24-form TC	Control group	16 weeks; four times a week; 60min per session	Proprioceptive function; Postural stability
Chang (2016)[121]	RCT	Elderly women	Simplified 24-form TC	Control group	24 weeks; four times a week; 60min per session	Knee and ankle kinaesthesia
Yang (2008)[122]	RCT	Older adults	7-form Chen-style TC+Qigong	Usual activities	20 weeks; three times a week; 60min per session	Blood
Pereira (2008)[123]	RCT	Elderly women	Simplified 24-form TC	Control group	12 weeks; three times a week; 50min per session	muscular strength of the knee extensors; Balance
Lu (2013)[124]	RCT	Elderly women	12-form Yang-style TC	Health education	16 weeks; twice a week; 90min per session	Arterial compliance; Knee joint muscle strength
Li, (2005)[125]	RCT	Older adults	Simplified 24-form TC	Stretching exercise	24 weeks; three times a week; 60min per session	Fear of falling
Thornton (2004)[126]	RCT	Middle-aged women	108-form Yang-style TC	Control group	12 weeks; three times a week; 60min per session	Balance; Blood pressure
Irwin (2012)[127]	RCT	Older adults	TC	Health education	16 weeks; three times a week; 40min per session	levels of interleukin 6; BDI; PSQI

Wolf (1996)[128]	RCT	Older adults	108-form Yang-style TC (10 movements)	C1: Balance training; C2: Education exercise	15 weeks; seven times a week; 30min per session	Cardiovascular endurance; Physical function; Fall rate; psychosocial indicators
Frye (2007)[129]	RCT	Older adults	10-form Yang-style TC	C1: Low impact exercise C2: Control group	12 weeks; three times a week; 60min per session	Physical Function; Psychological Well-Being; PSQI
Li(A) (2002)[130]	RCT	Older adults	Simplified 24-form TC	Usual activities	24 weeks; twice a week; 60min per session	Physical function; Self-esteem scale
Chen (1996)[131]	RCT	Older adults	Simplified 24-form TC	Usual activities	16 weeks; twice a week; 60min per session	TMAS
Holmes (2016)[132]	RCT	Older adults	5-form Yang-style TC	Health education	12 weeks; once a week; 60min per session	Posture control; Balance
Audette (2006)[133]	RCT	Elderly women	10-form Yang-style TC	C1: Brisk Walking; C2: Control group	12 weeks; three times a week; 60min per session	Heart rate; VO_{2max}; SF-36
Li (2001)[134]	RCT	Older adults	Simplified 24-form TC	Usual activities	24 weeks; twice a week; 60min per session	Self-efficacy; Physical function
Christou (2003)[135]	RCT	Older adults	12-form Chen-style TC	Control group	20 weeks; three times a week; 60min per session	Knee extensors
Tajik (2018)[136]	RCT	Older adults	Simplified 24-form TC (10 movements)	Usual activities	8 weeks; three times a week; 30-40min per session	AMTS; Quality of life
Wolf (1997)[137]	RCT	Older adults	108-form Yang-style TC (10 movements)	C1: Balance training; C2: Education exercise	15 weeks; twice a week; 60min per session	Posture control; Balance; Fear of fall
Young (1999)[138]	RCT	Older adults	13-form Yang-style TC	Aerobic exercise	12 weeks; twice a week; 60min per session	Blood pressure
Zheng (2018)[139]	RCT	Healthy but stressed people	Simplified 24-form TC	C1: Exercise group; C2: Control group	12 weeks; twice a week; 60min per session	PSS14; VAS; SF-36;

Note: TC = Tai Chi; C = control group; OA=osteoarthritis; NLBP = non-specific chronic lower back pain; FM = fibromyalgia; ACL = anterior cruciate ligament; HF = heart failure; CHD = coronary heart disease; PD = Parkinson disease; COPD = chronic obstructive pulmonary disease; WOMAC = Western Ontario and McMaster Universities Osteoarthritis Index; GUG = Get Up and Go test; STS = Sit-to-Stand test; PSQI = Pittsburgh Sleep Quality of Index ; SF-36 = 36-item Short Form Health Survey; MMSE = Mini-Mental State Examination; VDS = Verbal Descriptive Scale; TUG = Timed Up and Go; BBS = Berg Balance Scale; 6MWT = 6-min walk test; SCD = stair climb and descent ; VAS = Visual analog scale; ASES = Arthritis Self-Efficacy Scale; AIMS = Arthritis Impact Measurement Scale; SPPB = Short Physical Performance Battery; SF-12 = Short Form 12 Health Survey ;

sEMG = Wireless surface electromyography; NRS = numerical rating scale; FIQ = Fibromyalgia Impact Questionnaire; HADS = hospital anxiety and depression scale; HRV = heart rate variability; SRS = sit and reach score; HAQ = Health Assessment Questionnaire; BMD = bone mineral density; PSS = Perceived Stress Scale; NDI = Neck Disability Index; LKS = Lysholm Knee Scale; FFD = finger to floor distance; MFES = Modified Falls Efficacy Scale; BDI = Beck Depression Inventory; GHQ = General Health Questionnaire; SSRS = Social support rating scale; BRS = baroreflex sensitivity; LVEF = left ventricular ejection fraction; LVDD = left ventricular diastolic diameter; LVSD = left ventricular systolic diameter; CPET = Cardiopulmonary exercise testing; PDQ = Parkinson's Disease Questionnaire; VPS = Vitality Plus Scale; UPDRS = Unified Parkinson's Disease Rating Scale; ESS = Epworth Sleepiness Scale; TCS = Timed-Chair-Stand Test; HIT = Headache Impact Test; CRQ = Chronic Respiratory Disease questionnaire; SEMSOB = self-efficacy for managing shortness of breath; SGRQ-HKC = St George Respiratory questionnaire-Hong Kong Chinese version; COPD-CSES = COPD self-efficacy scale; mMRC = Medical Research Council dyspnea score; PHQ-9 = Nine-item patient health questionnaire depression scale; CPSS = The Chinese version of the Perceived stress scale; MAAS = Mindful attention and awareness scale; MSPSS = the Multidimensional Scale of Perceived Social Support DASS21 = the Depression Anxiety Stress Scale 21; GDS = Geriatric Depression Scale; PNSS = Positive and Negative Syndrome Scale; ADL = The Barthel's Activities of Daily Living index; FPI = fasting plasma insulin; FPG = fasting plasma glucose; HOMA2-IR = Homeostasis Model Assessment Index 2; MFSI-SF = Multidimensional Fatigue Symptom Inventory–Short Form; LUTSs = lower urinary tract symptoms; IPSS = International prostate symptom score; CSDD = The Cornell Scale for Depression in dementia; VDS = The Verbal Descriptive Scale; SMC = The Subjective Memory Complaints Scale; RBMT = The Rivermead Behavioral Memory Test; WAIS = Weschler Adult Intelligence Scale; PPA = Physiological Profile Assessment; RSES = Rosenberg Self-Esteem Scale; VAMS = Visual Analogue Mood Scales; FES-I = Fall Efficacy Scale-International; TMAS = Taylor Manifest Anxiety Scale; AMTS = Abbreviated mental test score.

References:

1. Tsai, P.; Chang, J.Y.; Beck, C.; Kuo, Y.; Keefe, F.J. A Pilot Cluster-Randomized Trial of a 20-Week Tai Chi Program in Elders with Cognitive Impairment and Osteoarthritic Knee: Effects on Pain and Other Health Outcomes. *J Pain Symptom Manag* **2013**, 45, 660-669, doi:10.1016/j.jpainsymman.2012.04.009.
2. Song, R.; Roberts, B.L.; Lee, E.O.; Lam, P.; Bae, S.C. A randomized study of the effects of t'ai chi on muscle strength, bone mineral density, and fear of falling in women with osteoarthritis. *J Altern Complement Med* **2010**, 16, 227-233, doi:10.1089/acm.2009.0165.
3. Wang, C.; Schmid, C.H.; Iversen, M.D.; Harvey, W.F.; Fielding, R.A.; Driban, J.B.; Price, L.L.; Wong, J.B.; Reid, K.F.; Rones, R., et al. Comparative Effectiveness of Tai Chi Versus Physical Therapy for Knee Osteoarthritis: A Randomized Trial. *Ann Intern Med* **2016**, 165, 77-86, doi:10.7326/M15-2143.
4. Lu, J.; Huang, L.; Wu, X.; Fu, W.; Liu, Y. Effect of Tai Ji Quan training on self-reported sleep quality in elderly Chinese women with knee osteoarthritis: a randomized controlled trail. *Sleep Med* **2017**, 33, 70-75, doi:10.1016/j.sleep.2016.12.024.
5. Song, R.; Lee, E.O.; Lam, P.; Bae, S.C. Effects of a Sun-style Tai Chi exercise on arthritic symptoms, motivation and the performance of health behaviors in women with osteoarthritis. *Taehan Kanho Hakhoe Chi* **2007**, 37, 249-256, doi:10.4040/jkan.2007.37.2.249.
6. Wortley, M.; Zhang, S.; Paquette, M.; Byrd, E.; Baumgartner, L.; Klipple, G.; Krusenklaus, J.; Brown, L. Effects of resistance and Tai Ji training on mobility and symptoms in knee osteoarthritis patients. *J Sport Health Sci* **2013**, 2, 209-214, doi:10.1016/j.jshs.2013.01.001.
7. Song, R.; Lee, E.O.; Lam, P.; Bae, S.C. Effects of tai chi exercise on pain, balance, muscle strength, and perceived difficulties in physical functioning in older women with osteoarthritis: a randomized clinical trial. *J Rheumatol* **2003**, 30, 2039-2044.
8. Hartman, C.A.; Manos, T.M.; Winter, C.; Hartman, D.M.; Li, B.; Smith, J.C. Effects of T'ai Chi training on function and quality of life indicators in older adults with osteoarthritis. *J Am Geriatr Soc* **2000**, 48, 1553-1559, doi:10.1111/j.1532-5415.2000.tb03863.x.
9. Zhu, Q.; Huang, L.; Wu, X.; Wang, L.; Zhang, Y.; Fang, M.; Liu, Y.; Li, J.X. Effects of Tai Ji Quan training on gait kinematics in older Chinese women with knee osteoarthritis: A randomized controlled trial. *J Sport Health Sci* **2016**, 5, 297-303, doi:10.1016/j.jshs.2016.02.003.
10. Callahan, L.F.; Cleveland, R.J.; Altpeter, M.; Hackney, B. Evaluation of Tai Chi Program Effectiveness for People with Arthritis in the Community: A Randomized Controlled Trial. *J Aging Phys Activ* **2016**, 24, 101-110, doi:10.1123/japa.2014-0211.
11. Brismee, J.M.; Paige, R.L.; Chyu, M.C.; Boatright, J.D.; Hagar, J.M.; McCaleb, J.A.; Quintela, M.M.; Feng, D.; Xu, K.T.; Shen, C.L. Group and home-based tai chi in elderly subjects with knee osteoarthritis: a randomized controlled trial. *Clin Rehabil* **2007**, 21, 99-111, doi:10.1177/0269215506070505.
12. Schmid, A.; McAlindon, T.; Schmid, C.H.; Wang, C. The Influence of Tai Chi Exercise on Proprioception in Patients with Knee Osteoarthritis: Results from a Pilot Randomized Controlled Trial. *International journal of integrative medicine* **2013**, 1, doi:10.5772/57137.
13. Fransen, M.; Nairn, L.; Winstanley, J.; Lam, P.; Edmonds, J. Physical activity for osteoarthritis management: a randomized controlled clinical trial evaluating hydrotherapy or Tai Chi classes. *Arthritis Rheum* **2007**, 57, 407-414, doi:10.1002/art.22621.
14. Ni, G.X.; Song, L.; Yu, B.; Huang, C.H.; Lin, J.H. Tai chi improves physical function in older Chinese women with knee osteoarthritis. *J Clin Rheumatol* **2010**, 16, 64-67, doi:10.1097/RHU.0b013e3181cf344f.
15. Wang, C.; Schmid, C.H.; Hibberd, P.L.; Kalish, R.; Roubenoff, R.; Rones, R.; McAlindon, T. Tai Chi is effective in treating knee osteoarthritis: a randomized controlled trial. *Arthritis Rheum* **2009**, 61, 1545-1553, doi:10.1002/art.24832.

16. Wu, W.; Muheremu, A.; Chen, C.; Liu, W.; Sun, L. Effectiveness of Tai Chi Practice for Non-Specific Chronic Low Back Pain on Retired Athletes: A Randomized Controlled Study. *J Musculoskelet Pain* **2013**, 21, 37-45, doi:10.3109/10582452.2013.763394.
17. Cho, Y. Effects of tai chi on pain and muscle activity in young males with acute low back pain. *J Phys Ther Sci* **2014**, 26, 679-681, doi:10.1589/jpts.26.679.
18. Hall, A.M.; Maher, C.G.; Lam, P.; Ferreira, M.; Latimer, J. Tai chi exercise for treatment of pain and disability in people with persistent low back pain: a randomized controlled trial. *Arthritis Care Res (Hoboken)* **2011**, 63, 1576-1583, doi:10.1002/acr.20594.
19. Zou, L.; Zhang, Y.; Liu, Y.; Tian, X.; Xiao, T.; Liu, X.; Yeung, A.S.; Liu, J.; Wang, X.; Yang, Q. The Effects of Tai Chi Chuan Versus Core Stability Training on Lower-Limb Neuromuscular Function in Aging Individuals with Non-Specific Chronic Lower Back Pain. *Medicina (Kaunas)* **2019**, 55, doi:10.3390/medicina55030060.
20. Jones, K.D.; Sherman, C.A.; Mist, S.D.; Carson, J.W.; Bennett, R.M.; Li, F. A randomized controlled trial of 8-form Tai chi improves symptoms and functional mobility in fibromyalgia patients. *Clin Rheumatol* **2012**, 31, 1205-1214, doi:10.1007/s10067-012-1996-2.
21. Wang, C.; Schmid, C.H.; Rones, R.; Kalish, R.; Yinh, J.; Goldenberg, D.L.; Lee, Y.; McAlindon, T. A randomized trial of tai chi for fibromyalgia. *N Engl J Med* **2010**, 363, 743-754, doi:10.1056/NEJMoa0912611.
22. Wang, C.; Schmid, C.H.; Fielding, R.A.; Harvey, W.F.; Reid, K.F.; Price, L.L.; Driban, J.B.; Kalish, R.; Rones, R.; McAlindon, T. Effect of tai chi versus aerobic exercise for fibromyalgia: comparative effectiveness randomized controlled trial. *BMJ* **2018**, 360, k851, doi:10.1136/bmj.k851.
23. Wong, A.; Figueroa, A.; Sanchez-Gonzalez, M.A.; Son, W.M.; Chernykh, O.; Park, S.Y. Effectiveness of Tai Chi on Cardiac Autonomic Function and Symptomatology in Women with Fibromyalgia: A Randomized Controlled Trial. *J Aging Phys Act* **2018**, 26, 214-221, doi:10.1123/japa.2017-0038.
24. Maddali, B.S.; Paoletti, G.; Cala, M.; Del, R.A.; El, A.K.; Mikhaylova, S. Efficacy of rehabilitation with Tai Ji Quan in an Italian cohort of patients with Fibromyalgia Syndrome. *Complement Ther Clin Pract* **2016**, 24, 109-115, doi:10.1016/j.ctcp.2016.05.010.
25. Maciaszek, J.; Osinski, W.; Szeklicki, R.; Stemplewski, R. Effect of Tai Chi on body balance: randomized controlled trial in men with osteopenia or osteoporosis. *Am J Chin Med* **2007**, 35, 1-9, doi:10.1142/S0192415X07004564.
26. Chyu, M.C.; James, C.R.; Sawyer, S.F.; Brismee, J.M.; Xu, K.T.; Poklikuha, G.; Dunn, D.M.; Shen, C.L. Effects of tai chi exercise on posturography, gait, physical function and quality of life in postmenopausal women with osteopaenia: a randomized clinical study. *Clin Rehabil* **2010**, 24, 1080-1090, doi:10.1177/0269215510375902.
27. Wayne, P.M.; Kiel, D.P.; Buring, J.E.; Connors, E.M.; Bonato, P.; Yeh, G.Y.; Cohen, C.J.; Mancinelli, C.; Davis, R.B. Impact of Tai Chi exercise on multiple fracture-related risk factors in post-menopausal osteopenic women: a pilot pragmatic, randomized trial. *BMC Complement Altern Med* **2012**, 12, 7, doi:10.1186/1472-6882-12-7.
28. Lauche, R.; Wayne, P.M.; Fehr, J.; Stumpe, C.; Dobos, G.; Cramer, H. Does Postural Awareness Contribute to Exercise-Induced Improvements in Neck Pain Intensity? A Secondary Analysis of a Randomized Controlled Trial Evaluating Tai Chi and Neck Exercises. *Spine (Phila Pa 1976)* **2017**, 42, 1195-1200, doi:10.1097/BRS.0000000000002078.
29. Lauche, R.; Stumpe, C.; Fehr, J.; Cramer, H.; Cheng, Y.W.; Wayne, P.M.; Rampp, T.; Langhorst, J.; Dobos, G. The Effects of Tai Chi and Neck Exercises in the Treatment of Chronic Nonspecific Neck Pain: A Randomized Controlled Trial. *J Pain* **2016**, 17, 1013-1027, doi:10.1016/j.jpain.2016.06.004.
30. You, T.; Ogawa, E.F.; Thapa, S.; Cai, Y.; Zhang, H.; Nagae, S.; Yeh, G.Y.; Wayne, P.M.; Shi, L.; Leveille, S.G. Tai Chi for older adults with chronic multisite pain: a randomized controlled pilot study. *Aging Clin Exp Res* **2018**, 30, 1335-1343, doi:10.1007/s40520-018-0922-0.
31. Wang, C. Tai Chi improves pain and functional status in adults with rheumatoid arthritis: results of a pilot single-blinded randomized controlled trial. *Med Sport Sci* **2008**, 52, 218-229, doi:10.1159/000134302.
32. Buyukturan, O.; Buyukturan, B.; Kurt, E.E.; Yetis, M. Effects of Tai Chi on partial anterior cruciate ligament injury: A single-blind, randomized-controlled trial. *Turk J Phys Med*

Rehabil **2019**, 65, 160-168, doi:10.5606/tftrd.2019.2798.

33. Lee, E.N.; Kim, Y.H.; Chung, W.T.; Lee, M.S. Tai chi for disease activity and flexibility in patients with ankylosing spondylitis--a controlled clinical trial. *Evid Based Complement Alternat Med* **2008**, 5, 457-462, doi:10.1093/ecam/nem048.
34. Taylor-Piliae, R.E.; Hoke, T.M.; Hepworth, J.T.; Latt, L.D.; Najafi, B.; Coull, B.M. Effect of Tai Chi on physical function, fall rates and quality of life among older stroke survivors. *Arch Phys Med Rehabil* **2014**, 95, 816-824, doi:10.1016/j.apmr.2014.01.001.
35. Chan, W.N.; Tsang, W.W. Effect of Tai Chi Training on Dual-Tasking Performance That Involves Stepping Down among Stroke Survivors: A Pilot Study. *Evid Based Complement Alternat Med* **2017**, 2017, 9134173, doi:10.1155/2017/9134173.
36. Xie, G.; Rao, T.; Lin, L.; Lin, Z.; Xiao, T.; Yang, M.; Xu, Y.; Fan, J.; Lin, S.; Wu, J., et al. Effects of Tai Chi Yunshou exercise on community-based stroke patients: a cluster randomized controlled trial. *Eur Rev Aging Phys a* **2018**, 15, doi:10.1186/s11556-018-0206-x.
37. Kim, H.; Kim, Y.L.; Lee, S.M. Effects of therapeutic Tai Chi on balance, gait, and quality of life in chronic stroke patients. *Int J Rehabil Res* **2015**, 38, 156-161, doi:10.1097/MRR.0000000000000103.
38. Au-Yeung, S.S.; Hui-Chan, C.W.; Tang, J.C. Short-form Tai Chi improves standing balance of people with chronic stroke. *Neurorehabil Neural Repair* **2009**, 23, 515-522, doi:10.1177/1545968308326425.
39. Wang, W.; Sawada, M.; Noriyama, Y.; Arita, K.; Ota, T.; Sadamatsu, M.; Kiyotou, R.; Hirai, M.; Kishimoto, T. Tai Chi exercise versus rehabilitation for the elderly with cerebral vascular disorder: a single-blinded randomized controlled trial. *Psychogeriatrics* **2010**, 10, 160-166, doi:10.1111/j.1479-8301.2010.00334.x.
40. Redwine, L.S.; Tsuang, M.; Rusiewicz, A.; Pandzic, I.; Cammarata, S.; Rutledge, T.; Hong, S.; Linke, S.; Mills, P.J. A pilot study exploring the effects of a 12-week t'ai chi intervention on somatic symptoms of depression in patients with heart failure. *J Altern Complement Med* **2012**, 18, 744-748, doi:10.1089/acm.2011.0314.
41. Barrow, D.E.; Bedford, A.; Ives, G.; O'Toole, L.; Channer, K.S. An evaluation of the effects of Tai Chi Chuan and Chi Kung training in patients with symptomatic heart failure: a randomised controlled pilot study. *Postgrad Med J* **2007**, 83, 717-721, doi:10.1136/pgmj.2007.061267.
42. Yeh, G.Y.; Wood, M.J.; Lorell, B.H.; Stevenson, L.W.; Eisenberg, D.M.; Wayne, P.M.; Goldberger, A.L.; Davis, R.B.; Phillips, R.S. Effects of tai chi mind-body movement therapy on functional status and exercise capacity in patients with chronic heart failure: a randomized controlled trial. *Am J Med* **2004**, 117, 541-548, doi:10.1016/j.amjmed.2004.04.016.
43. Yeh, G.Y.; Mietus, J.E.; Peng, C.K.; Phillips, R.S.; Davis, R.B.; Wayne, P.M.; Goldberger, A.L.; Thomas, R.J. Enhancement of sleep stability with Tai Chi exercise in chronic heart failure: preliminary findings using an ECG-based spectrogram method. *Sleep Med* **2008**, 9, 527-536, doi:10.1016/j.sleep.2007.06.003.
44. Yeh, G.Y.; Wayne, P.M.; Phillips, R.S. T'ai Chi exercise in patients with chronic heart failure. *Med Sport Sci* **2008**, 52, 195-208, doi:10.1159/000134300.
45. Yeh, G.Y.; McCarthy, E.P.; Wayne, P.M.; Stevenson, L.W.; Wood, M.J.; Forman, D.; Davis, R.B.; Phillips, R.S. Tai chi exercise in patients with chronic heart failure: a randomized clinical trial. *Arch Intern Med* **2011**, 171, 750-757, doi:10.1001/archinternmed.2011.150.
46. Yeh, G.Y.; Wood, M.J.; Wayne, P.M.; Quilty, M.T.; Stevenson, L.W.; Davis, R.B.; Phillips, R.S.; Forman, D.E. Tai chi in patients with heart failure with preserved ejection fraction. *Congest Heart Fail* **2013**, 19, 77-84, doi:10.1111/chf.12005.
47. Shou, X.L.; Wang, L.; Jin, X.Q.; Zhu, L.Y.; Ren, A.H.; Wang, Q.N. Effect of T'ai Chi Exercise on Hypertension in Young and Middle-Aged In-Service Staff. *J Altern Complement Med* **2019**, 25, 73-78, doi:10.1089/acm.2018.0011.
48. Chan, A.; Chair, S.Y.; Lee, D.; Leung, D.; Sit, J.; Cheng, H.Y.; Taylor-Piliae, R.E. Tai Chi exercise is more effective than brisk walking in reducing cardiovascular disease risk factors among adults with hypertension: A randomised controlled trial. *Int J Nurs Stud* **2018**, 88, 44-52, doi:10.1016/j.ijnurstu.2018.08.009.

49. Tsai, J.C.; Wang, W.H.; Chan, P.; Lin, L.J.; Wang, C.H.; Tomlinson, B.; Hsieh, M.H.; Yang, H.Y.; Liu, J.C. The beneficial effects of Tai Chi Chuan on blood pressure and lipid profile and anxiety status in a randomized controlled trial. *J Altern Complement Med* **2003**, 9, 747-754, doi:10.1089/107555303322524599.
50. Ma, C.; Zhou, W.; Tang, Q.; Huang, S. The impact of group-based Tai chi on health-status outcomes among community-dwelling older adults with hypertension. *Heart Lung* **2018**, 47, 337-344, doi:10.1016/j.hrtlng.2018.04.007.
51. Sato, S.; Makita, S.; Uchida, R.; Ishihara, S.; Masuda, M. Effect of Tai Chi Training on Baroreflex Sensitivity and Heart Rate Variability in Patients with Coronary Heart Disease. *Int Heart J* **2010**, 51, 238-241, doi:10.1536/ihj.51.238.
52. Chang, R.Y.; Koo, M.; Kan, C.B.; Yu, Z.R.; Chu, I.T.; Hsu, C.T.; Chen, C.Y. Effects of tai chi rehabilitation on heart rate responses in patients with coronary artery disease. *Am J Chinese Med* **2010**, 38, 461-472, doi:10.1142/S0192415X10007981.
53. Liu, J.; Li, B.; Shnider, R. EFFECTS OF TAI CHI TRAINING ON IMPROVING PHYSICAL FUNCTION IN PATIENTS WITH CORONARY HEART DISEASES. *J Exerc Sci Fit* **2010**, 8, 78-84, doi:10.1016/S1728-869X(10)60012-3.
54. Salmoirago-Blotcher, E.; Wayne, P.M.; Dunsiger, S.; Krol, J.; Breault, C.; Bock, B.C.; Wu, W.; Yeh, G.Y. Tai Chi Is a Promising Exercise Option for Patients with Coronary Heart Disease Declining Cardiac Rehabilitation. *J Am Heart Assoc* **2017**, 6, doi:10.1161/JAHA.117.006603.
55. Nery, R.M.; Zanini, M.; de Lima, J.B.; Buhler, R.P.; Da, S.A.; Stein, R. Tai Chi Chuan improves functional capacity after myocardial infarction: A randomized clinical trial. *Am Heart J* **2015**, 169, 854-860, doi:10.1016/j.ahj.2015.01.017.
56. Li, F.; Harmer, P.; Liu, Y.; Eckstrom, E.; Fitzgerald, K.; Stock, R.; Chou, L. A randomized controlled trial of patient-reported outcomes with tai chi exercise in Parkinson's disease. *Movement Disord* **2014**, 29, 539-545, doi:10.1002/mds.25787.
57. Choi, H.J. Effects of therapeutic Tai chi on functional fitness and activities of daily living in patients with Parkinson disease. *J Exerc Rehabil* **2016**, 12, 499-503, doi:10.12965/jer.1632654.327.
58. Li, F.; Harmer, P.; Fitzgerald, K.; Eckstrom, E.; Stock, R.; Galver, J.; Maddalozzo, G.; Batya, S.S. Tai chi and postural stability in patients with Parkinson's disease. *N Engl J Med* **2012**, 366, 511-519, doi:10.1056/NEJMoa1107911.
59. Hackney, M.E.; Earhart, G.M. Tai Chi improves balance and mobility in people with Parkinson disease. *Gait Posture* **2008**, 28, 456-460, doi:10.1016/j.gaitpost.2008.02.005.
60. Cheon, S.; Chae, B.; Sung, H.; Lee, G.C.; Kim, J.W. The Efficacy of Exercise Programs for Parkinson's Disease: Tai Chi versus Combined Exercise. *J Clin Neurol* **2013**, 9, 237-243, doi:10.3988/jcn.2013.9.4.237.
61. Choi, H.J.; Garber, C.E.; Jun, T.W.; Jin, Y.S.; Chung, S.J.; Kang, H.J. Therapeutic effects of tai chi in patients with Parkinson's disease. *ISRN Neurol* **2013**, 2013, 548240, doi:10.1155/2013/548240.
62. Li, F.; Fisher, K.J.; Harmer, P.; Irbe, D.; Tearse, R.G.; Weimer, C. Tai chi and self-rated quality of sleep and daytime sleepiness in older adults: a randomized controlled trial. *J Am Geriatr Soc* **2004**, 52, 892-900, doi:10.1111/j.1532-5415.2004.52255.x.
63. Liu, J.; Kwan, R.; Lai, C.K.; Hill, K.D. A simplified 10-step Tai-chi programme to enable people with dementia to improve their motor performance: a feasibility study. *Clin Rehabil* **2018**, 32, 1609-1623, doi:10.1177/0269215518786530.
64. Nyman, S.R.; Ingram, W.; Sanders, J.; Thomas, P.W.; Thomas, S.; Vassallo, M.; Raftery, J.; Bibi, I.; Barrado-Martin, Y. Randomised Controlled Trial of The Effect of Tai Chi on Postural Balance of People with Dementia. *Clin Interv Aging* **2019**, 14, 2017-2029, doi:10.2147/CIA.S228931.
65. Abbott, R.B.; Hui, K.K.; Hays, R.D.; Li, M.D.; Pan, T. A randomized controlled trial of tai chi for tension headaches. *Evid Based Complement Alternat Med* **2007**, 4, 107-113,

doi:10.1093/ecam/nel050.
66. Zhu, S.; Shi, K.; Yan, J.; He, Z.; Wang, Y.; Yi, Q.; Huang, H. A modified 6-form Tai Chi for patients with COPD. *Complement Ther Med* **2018**, 39, 36-42, doi:10.1016/j.ctim.2018.05.007.
67. Ng, L.; Chiang, L.K.; Tang, R.; Siu, C.; Fung, L.; Lee, A.; Tam, W. Effectiveness of incorporating Tai Chi in a pulmonary rehabilitation program for Chronic Obstructive Pulmonary Disease (COPD) in primary care-A pilot randomized controlled trial. *Eur J Integr Med* **2014**, 6, 248-258, doi:10.1016/j.eujim.2014.01.007.
68. Leung, R.W.; McKeough, Z.J.; Peters, M.J.; Alison, J.A. Short-form Sun-style t'ai chi as an exercise training modality in people with COPD. *Eur Respir J* **2013**, 41, 1051-1057, doi:10.1183/09031936.00036912.
69. Polkey, M.I.; Qiu, Z.H.; Zhou, L.; Zhu, M.D.; Wu, Y.X.; Chen, Y.Y.; Ye, S.P.; He, Y.S.; Jiang, M.; He, B.T., et al. Tai Chi and Pulmonary Rehabilitation Compared for Treatment-Naive Patients With COPD: A Randomized Controlled Trial. *Chest* **2018**, 153, 1116-1124, doi:10.1016/j.chest.2018.01.053.
70. Yeh, G.Y.; Roberts, D.H.; Wayne, P.M.; Davis, R.B.; Quilty, M.T.; Phillips, R.S. Tai chi exercise for patients with chronic obstructive pulmonary disease: a pilot study. *Respir Care* **2010**, 55, 1475-1482.
71. Niu, R.; He, R.; Luo, B.L.; Hu, C. The effect of tai chi on chronic obstructive pulmonary disease: a pilot randomised study of lung function, exercise capacity and diaphragm strength. *Heart Lung Circ* **2014**, 23, 347-352, doi:10.1016/j.hlc.2013.10.057.
72. Zhang, J.; Qin, S.; Zhou, Y.; Meng, L.; Su, H.; Zhao, S. A randomized controlled trial of mindfulness-based Tai Chi Chuan for subthreshold depression adolescents. *Neuropsychiatr Dis Treat* **2018**, 14, 2313-2321, doi:10.2147/NDT.S173255.
73. Lavretsky, H.; Alstein, L.L.; Olmstead, R.E.; Ercoli, L.M.; Riparetti-Brown, M.; Cyr, N.S.; Irwin, M.R. Complementary use of tai chi chih augments escitalopram treatment of geriatric depression: a randomized controlled trial. *Am J Geriatr Psychiatry* **2011**, 19, 839-850, doi:10.1097/JGP.0b013e31820ee9ef.
74. Yeung, A.; Lepoutre, V.; Wayne, P.; Yeh, G.; Slipp, L.E.; Fava, M.; Denninger, J.W.; Benson, H.; Fricchione, G.L. Tai chi treatment for depression in Chinese Americans: a pilot study. *Am J Phys Med Rehabil* **2012**, 91, 863-870, doi:10.1097/PHM.0b013e31825f1a67.
75. Liao, S.J.; Chong, M.C.; Tan, M.P.; Chua, Y.P. Tai Chi with music improves quality of life among community-dwelling older persons with mild to moderate depressive symptoms: A cluster randomized controlled trial. *Geriatr Nurs* **2019**, 40, 154-159, doi:10.1016/j.gerinurse.2018.08.001.
76. Liu, X.; Vitetta, L.; Kostner, K.; Crompton, D.; Williams, G.; Brown, W.J.; Lopez, A.; Xue, C.C.; Oei, T.P.; Byrne, G., et al. The effects of tai chi in centrally obese adults with depression symptoms. *Evid Based Complement Alternat Med* **2015**, 2015, 879712, doi:10.1155/2015/879712.
77. Liu, J.; Xie, H.; Liu, M.; Wang, Z.; Zou, L.; Yeung, A.S.; Hui, S.S.; Yang, Q. The Effects of Tai Chi on Heart Rate Variability in Older Chinese Individuals with Depression. *Int J Environ Res Public Health* **2018**, 15, doi:10.3390/ijerph15122771.
78. Ho, R.T.; Fong, T.C.; Wan, A.H.; Au-Yeung, F.S.; Wong, C.P.; Ng, W.Y.; Cheung, I.K.; Lo, P.H.; Ng, S.M.; Chan, C.L., et al. A randomized controlled trial on the psychophysiological effects of physical exercise and Tai-chi in patients with chronic schizophrenia. *Schizophr Res* **2016**, 171, 42-49, doi:10.1016/j.schres.2016.01.038.
79. Zhang, Y.; Fu, F.H. Effects of 14-week Tai Ji Quan exercise on metabolic control in women with type 2 diabetes. *Am J Chin Med* **2008**, 36, 647-654, doi:10.1142/S0192415X08006119.
80. Tsang, T.; Orr, R.; Lam, P.; Comino, E.; Singh, M.F. Effects of Tai Chi on glucose homeostasis and insulin sensitivity in older adults with type 2 diabetes: a randomised double-blind sham-exercise-controlled trial. *Age Ageing* **2008**, 37, 64-71, doi:10.1093/ageing/afm127.
81. Tsang, T.; Orr, R.; Lam, P.; Comino, E.J.; Singh, M.F. Health benefits of Tai Chi for older patients with type 2 diabetes: the "Move It For Diabetes study"--a randomized controlled

trial. *Clin Interv Aging* **2007**, 2, 429-439.
82. Liu, X.; Miller, Y.D.; Burton, N.W.; Chang, J.H.; Brown, W.J. The effect of Tai Chi on health-related quality of life in people with elevated blood glucose or diabetes: a randomized controlled trial. *Qual Life Res* **2013**, 22, 1783-1786, doi:10.1007/s11136-012-0311-7.
83. Choi, Y.S.; Song, R.; Ku, B.J. Effects of a T'ai Chi-Based Health Promotion Program on Metabolic Syndrome Markers, Health Behaviors, and Quality of Life in Middle-Aged Male Office Workers: A Randomized Trial. *J Altern Complement Med* **2017**, 23, 949-956, doi:10.1089/acm.2017.0057.
84. Zhang, Y.; Wang, R.; Chen, P.; Yu, D. Effects of Tai Chi Chuan training on cellular immunity in post-surgical non-small cell lung cancer survivors: A randomized pilot trial. *J Sport Health Sci* **2013**, 2, 104-108, doi:10.1016/j.jshs.2013.02.001.
85. Campo, R.A.; O'Connor, K.; Light, K.C.; Nakamura, Y.; Lipschitz, D.L.; LaStayo, P.C.; Pappas, L.; Boucher, K.; Irwin, M.R.; Agarwal, N., et al. Feasibility and acceptability of a Tai Chi Chih randomized controlled trial in senior female cancer survivors. *Integr Cancer Ther* **2013**, 12, 464-474, doi:10.1177/1534735413485418.
86. Zhang, L.L.; Wang, S.Z.; Chen, H.L.; Yuan, A.Z. Tai Chi Exercise for Cancer-Related Fatigue in Patients with Lung Cancer Undergoing Chemotherapy: A Randomized Controlled Trial. *J Pain Symptom Manage* **2016**, 51, 504-511, doi:10.1016/j.jpainsymman.2015.11.020.
87. Jung, S.; Lee, E.N.; Lee, S.R.; Kim, M.S.; Lee, M.S. Tai chi for lower urinary tract symptoms and quality of life in elderly patients with benign prostate hypertrophy: a randomized controlled trial. *Evid Based Complement Alternat Med* **2012**, 2012, 624692, doi:10.1155/2012/624692.
88. Lam, L.C.; Chau, R.C.; Wong, B.M.; Fung, A.W.; Tam, C.W.; Leung, G.T.; Kwok, T.C.; Leung, T.Y.; Ng, S.P.; Chan, W.M. A 1-year randomized controlled trial comparing mind body exercise (Tai Chi) with stretching and toning exercise on cognitive function in older Chinese adults at risk of cognitive decline. *J Am Med Dir Assoc* **2012**, 13, 515-568, doi:10.1016/j.jamda.2012.03.008.
89. Tsai, P.F.; Chang, J.Y.; Beck, C.; Kuo, Y.F.; Keefe, F.J.; Rosengren, K. A supplemental report to a randomized cluster trial of a 20-week Sun-style Tai Chi for osteoarthritic knee pain in elders with cognitive impairment. *Complement Ther Med* **2015**, 23, 570-576, doi:10.1016/j.ctim.2015.06.001.
90. Kasai, J.Y.T.; Busse, A.L.; Magaldi, R.M.; Soci, M.A.; Rosa, P.D.M.; Curiati, J.A.E.; Jacob Filho, W. Effects of Tai Chi Chuan on cognition of elderly women with mild cognitive impairment. *Einstein (Sao Paulo, Brazil)* **2010**, 8, 40-45, doi:10.1590/S1679-45082010AO1470.
91. Sungkarat, S.; Boripuntakul, S.; Chattipakorn, N.; Watcharasaksilp, K.; Lord, S.R. Effects of Tai Chi on Cognition and Fall Risk in Older Adults with Mild Cognitive Impairment: A Randomized Controlled Trial. *J Am Geriatr Soc* **2017**, 65, 721-727, doi:10.1111/jgs.14594.
92. Maciaszek, J.; Osinski, W. Effect of Tai Chi on body balance: randomized controlled trial in elderly men with dizziness. *Am J Chin Med* **2012**, 40, 245-253, doi:10.1142/S0192415X1250019X.
93. Kong, Z.; Sze, T.M.; Yu, J.J.; Loprinzi, P.D.; Xiao, T.; Yeung, A.S.; Li, C.; Zhang, H.; Zou, L. Tai Chi as an Alternative Exercise to Improve Physical Fitness for Children and Adolescents with Intellectual Disability. *Int J Environ Res Public Health* **2019**, 16, doi:10.3390/ijerph16071152.
94. Chen, E.W.; Fu, A.S.; Chan, K.M.; Tsang, W.W. The effects of Tai Chi on the balance control of elderly persons with visual impairment: a randomised clinical trial. *Age Ageing* **2012**, 41, 254-259, doi:10.1093/ageing/afr146.
95. Gemmell, C.; Leathem, J.M. A study investigating the effects of Tai Chi Chuan: individuals with traumatic brain injury compared to controls. *Brain Inj* **2006**, 20, 151-156, doi:10.1080/02699050500442998.
96. Voukelatos, A.; Cumming, R.G.; Lord, S.R.; Rissel, C. A randomized, controlled trial of tai chi for the prevention of falls: the Central Sydney tai chi trial. *J Am Geriatr Soc* **2007**, 55, 1185-1191, doi:10.1111/j.1532-5415.2007.01244.x.

97. Yang, Y.; Verkuilen, J.V.; Rosengren, K.S.; Grubisich, S.A.; Reed, M.R.; Hsiao-Wecksler, E.T. Effect of combined Taiji and Qigong training on balance mechanisms: a randomized controlled trial of older adults. *Med Sci Monit* **2007**, 13, R339-R348.
98. Li, F.; Harmer, P.; Fitzgerald, K.; Eckstrom, E.; Akers, L.; Chou, L.S.; Pidgeon, D.; Voit, J.; Winters-Stone, K. Effectiveness of a Therapeutic Tai Ji Quan Intervention vs a Multimodal Exercise Intervention to Prevent Falls Among Older Adults at High Risk of Falling: A Randomized Clinical Trial. *Jama Intern Med* **2018**, 178, 1301-1310, doi:10.1001/jamainternmed.2018.3915.
99. Zhou, J.; Chang, S.; Cong, Y.; Qin, M.; Sun, W.; Lian, J.; Yao, J.; Li, W.; Hong, Y. Effects of 24 weeks of Tai Chi Exercise on Postural Control among Elderly Women. *Res Sports Med* **2015**, 23, 302-314, doi:10.1080/15438627.2015.1040918.
100. Lelard, T.; Doutrellot, P.L.; David, P.; Ahmaidi, S. Effects of a 12-week Tai Chi Chuan program versus a balance training program on postural control and walking ability in older people. *Arch Phys Med Rehabil* **2010**, 91, 9-14, doi:10.1016/j.apmr.2009.09.014.
101. Li, Y.; Devault, C.N.; Van Oteghen, S. Effects of extended Tai Chi intervention on balance and selected motor functions of the elderly. *Am J Chin Med* **2007**, 35, 383-391, doi:10.1142/S0192415X07004904.
102. Hwang, H.F.; Chen, S.J.; Lee-Hsieh, J.; Chien, D.K.; Chen, C.Y.; Lin, M.R. Effects of Home-Based Tai Chi and Lower Extremity Training and Self-Practice on Falls and Functional Outcomes in Older Fallers from the Emergency Department-A Randomized Controlled Trial. *J Am Geriatr Soc* **2016**, 64, 518-525, doi:10.1111/jgs.13952.
103. Sun, W.; Ma, X.; Wang, L.; Zhang, C.; Song, Q.; Gu, H.; Mao, D. Effects of Tai Chi Chuan and Brisk Walking Exercise on Balance Ability in Elderly Women: A Randomized Controlled Trial. *Motor Control* **2019**, 23, 100-114, doi:10.1123/mc.2017-0055.
104. Kim, H. Effects of Tai Chi Exercise on the Center of Pressure Trace during Obstacle Crossing in Older Adults who are at a Risk of Falling. *JOURNAL OF PHYSICAL THERAPY SCIENCE* **2009**, 21, 49-54, doi:10.1589/jpts.21.49.
105. Li, F.; Harmer, P.; Fisher, K.J.; McAuley, E.; Chaumeton, N.; Eckstrom, E.; Wilson, N.L. Tai Chi and fall reductions in older adults: a randomized controlled trial. *J Gerontol A Biol Sci Med Sci* **2005**, 60, 187-194, doi:10.1093/gerona/60.2.187.
106. Hosseini, L.; Kargozar, E.; Sharifi, F.; Negarandeh, R.; Memari, A.H.; Navab, E. Tai Chi Chuan can improve balance and reduce fear of falling in community dwelling older adults: a randomized control trial. *J Exerc Rehabil* **2018**, 14, 1024-1031, doi:10.12965/jer.1836488.244.
107. Li, F.; Harmer, P.; Fisher, K.J.; McAuley, E. Tai Chi: improving functional balance and predicting subsequent falls in older persons. *Med Sci Sports Exerc* **2004**, 36, 2046-2052, doi:10.1249/01.mss.0000147590.54632.e7.
108. Mortazavi, H.; Tabatabaeichehr, M.; Golestani, A.; Armat, M.R.; Yousefi, M.R. The Effect of Tai Chi Exercise on the Risk and Fear of Falling in Older Adults: a Randomized Clinical Trial. *Materia socio-medica* **2018**, 30, 38-42, doi:10.5455/msm.2018.30.38-42.
109. Zhang, J.G.; Ishikawa-Takata, K.; Yamazaki, H.; Morita, T.; Ohta, T. The effects of Tai Chi Chuan on physiological function and fear of falling in the less robust elderly: an intervention study for preventing falls. *Arch Gerontol Geriatr* **2006**, 42, 107-116, doi:10.1016/j.archger.2005.06.007.
110. Nguyen, M.H.; Kruse, A. A randomized controlled trial of Tai chi for balance, sleep quality and cognitive performance in elderly Vietnamese. *Clin Interv Aging* **2012**, 7, 185-190, doi:10.2147/CIA.S32600.
111. Chan, K.; Qin, L.; Lau, M.; Woo, J.; Au, S.; Choy, W.; Lee, K.; Lee, S. A randomized, prospective study of the effects of Tai Chi Chun exercise on bone mineral density in postmenopausal women. *Arch Phys Med Rehabil* **2004**, 85, 717-722, doi:10.1016/j.apmr.2003.08.091.
112. Wayne, P.M.; Manor, B.; Novak, V.; Costa, M.D.; Hausdorff, J.M.; Goldberger, A.L.; Ahn, A.C.; Yeh, G.Y.; Peng, C.K.; Lough, M., et al. A systems biology approach to studying

Tai Chi, physiological complexity and healthy aging: Design and rationale of a pragmatic randomized controlled trial. *Contemp Clin Trials* **2013**, 34, 21-34, doi:10.1016/j.cct.2012.09.006.

113. Li, F.Z.; Harmer, P.; McAuley, E.; Duncan, T.E.; Duncan, S.C.; Chaumeton, N.; Fisher, K.J. An evaluation of the effects of Tai Chi exercise on physical function among older persons: A randomized controlled trial. *Ann Behav Med* **2001**, 23, 139-146, doi:10.1207/S15324796ABM2302_9.

114. Schitter, A.M.; Nedeljkovic, M.; Ausfeld-Hafter, B.; Fleckenstein, J. Changes in self-reported symptoms of depression and physical well-being in healthy individuals following a Taiji beginner course - Results of a randomized controlled trial. *Brain Behav* **2016**, 6, doi:10.1002/brb3.429.

115. Shen, C.L.; Williams, J.S.; Chyu, M.C.; Paige, R.L.; Stephens, A.L.; Chauncey, K.B.; Prabhu, F.R.; Ferris, L.T.; Yeh, J.K. Comparison of the effects of Tai Chi and resistance training on bone metabolism in the elderly: a feasibility study. *Am J Chin Med* **2007**, 35, 369-381, doi:10.1142/S0192415X07004898.

116. Li, F.; Fisher, K.J.; Harmer, P.; McAuley, E. Delineating the impact of Tai Chi training on physical function among the elderly. *Am J Prev Med* **2002**, 23, 92-97, doi:10.1016/s0749-3797(02)00479-8.

117. Sun, W.; Wang, L.; Zhang, C.; Song, Q.; Gu, H.; Mao, D. Detraining effects of regular Tai Chi exercise on postural control ability in older women: A randomized controlled trial. *J Exerc Sci Fit* **2018**, 16, 55-61, doi:10.1016/j.jesf.2018.06.003.

118. Sun, W.; Zhang, C.; Song, Q.; Li, W.; Cong, Y.; Chang, S.; Mao, D.; Hong, Y. Effect of 1-year regular Tai Chi on neuromuscular reaction in elderly women: a randomized controlled study. *Res Sports Med* **2016**, 24, 145-156, doi:10.1080/15438627.2015.1126280.

119. Zou, L.; Wang, C.; Tian, Z.; Wang, H.; Shu, Y. Effect of Yang-Style Tai Chi on Gait Parameters and Musculoskeletal Flexibility in Healthy Chinese Older Women. *Sports (Basel, Switzerland)* **2017**, 5, doi:10.3390/sports5030052.

120. Li, J.X.; Xu, D.Q.; Hong, Y. Effects of 16-week Tai Chi intervention on postural stability and proprioception of knee and ankle in older people. *Age Ageing* **2008**, 37, 575-578, doi:10.1093/ageing/afn109.

121. Chang, S.; Zhou, J.; Hong, Y.; Sun, W.; Cong, Y.; Qin, M.; Lian, J.; Yao, J.; Li, W. Effects of 24-week Tai Chi exercise on the knee and ankle proprioception of older women. *Res Sports Med* **2016**, 24, 84-93, doi:10.1080/15438627.2015.1126281.

122. Yang, Y.; Verkuilen, J.; Rosengren, K.S.; Mariani, R.A.; Reed, M.; Grubisich, S.A.; Woods, J.A.; Schlagal, B. Effects of a traditional Taiji/Qigong curriculum on older adults' immune response to influenza vaccine. *Med Sport Sci* **2008**, 52, 64-76, doi:10.1159/000134285.

123. Pereira, M.M.; Oliveira, R.J.; Silva, M.A.F.; Souza, L.H.R.; Vianna, L.G. Effects of Tai Chi Chuon on knee extensor muscle strength and balance in elderly women. *Braz J Phys Ther* **2008**, 12, 121-126, doi:10.1590/S1413-35552008000200008.

124. Lu, X.; Hui-Chan, C.W.; Tsang, W.W. Effects of Tai Chi training on arterial compliance and muscle strength in female seniors: a randomized clinical trial. *Eur J Prev Cardiol* **2013**, 20, 238-245, doi:10.1177/2047487311434233.

125. Li, F.; Fisher, K.J.; Harmer, P.; McAuley, E. Falls self-efficacy as a mediator of fear of falling in an exercise intervention for older adults. *J Gerontol B Psychol Sci Soc Sci* **2005**, 60, P34-P40, doi:10.1093/geronb/60.1.p34.

126. Thornton, E.W.; Sykes, K.S.; Tang, W.K. Health benefits of Tai Chi exercise: improved balance and blood pressure in middle-aged women. *Health Promot Int* **2004**, 19, 33-38, doi:10.1093/heapro/dah105.

127. Irwin, M.R.; Olmstead, R. Mitigating cellular inflammation in older adults: a randomized controlled trial of Tai Chi Chih. *Am J Geriatr Psychiatry* **2012**, 20, 764-772, doi:10.1097/JGP.0b013e3182330fd3.

128. Wolf, S.L.; Barnhart, H.X.; Kutner, N.G.; McNeely, E.; Coogler, C.; Xu, T.S.; Clements, S.D.; Connell, B.R.; Fletcher, R.J.; Green, R., et al. Reducing frailty and falls in older persons: An investigation of Tai Chi and computerized balance training. *J Am Geriatr Soc* **1996**, 44, 489-497, doi:10.1111/j.1532-5415.1996.tb01432.x.
129. Frye, B.; Scheinthal, S.; Kemarskaya, T.; Pruchno, R. Tai chi and low impact exercise: Effects on the physical functioning and psychological well-being of older people. *J Appl Gerontol* **2007**, 26, 433-453, doi:10.1177/0733464807306915.
130. Li, F.Z.; Harmer, P.; Chaumeton, N.R.; Duncan, T.E.; Duncan, S.C. Tai Chi as a means to enhance self-esteem: A randomized controlled trial. *J Appl Gerontol* **2002**, 21, 70-89, doi:10.1177/073346480202100105.
131. Chen, W.W.; Sun, W.Y. Tai chi chuan, an alternative form of exercise for health promotion and disease prevention for older adults in the community. *International quarterly of community health education* **1996**, 16, 333-339, doi:10.2190/FDPE-VVG2-VNTR-N2DK.
132. Holmes, M.L.; Manor, B.; Hsieh, W.; Hu, K.; Lipsitz, L.A.; Li, L. Tai Chi training reduced coupling between respiration and postural control. *Neurosci Lett* **2016**, 610, 60-65, doi:10.1016/j.neulet.2015.10.053.
133. Audette, J.F.; Jin, Y.S.; Newcomer, R.; Stein, L.; Duncan, G.; Frontera, W.R. Tai Chi versus brisk walking in elderly women. *Age Ageing* **2006**, 35, 388-393, doi:10.1093/ageing/afl006.
134. Li, F.; Harmer, P.; McAuley, E.; Fisher, K.J.; Duncan, T.E.; Duncan, S.C. Tai Chi, self-efficacy, and physical function in the elderly. *Prev Sci* **2001**, 2, 229-239, doi:10.1023/a:1013614200329.
135. Christou, E.A.; Yang, Y.; Rosengren, K.S. Taiji training improves knee extensor strength and force control in older adults. *J Gerontol A Biol Sci Med Sci* **2003**, 58, 763-766, doi:10.1093/gerona/58.8.m763.
136. Tajik, A.; Rejeh, N.; Heravi-Karimooi, M.; Samady, K.P.; Tadrisi, S.D.; Watts, T.E.; Griffiths, P.; Vaismoradi, M. The effect of Tai Chi on quality of life in male older people: A randomized controlled clinical trial. *Complement Ther Clin Pract* **2018**, 33, 191-196, doi:10.1016/j.ctcp.2018.10.009.
137. Wolf, S.L.; Barnhart, H.X.; Ellison, G.L.; Coogler, C.E. The effect of Tai Chi Quan and computerized balance training on postural stability in older subjects. *Phys Ther* **1997**, 77, 371-381, doi:10.1093/ptj/77.4.371.
138. Young, D.R.; Appel, L.J.; Jee, S.; Miller, E.R. The effects of aerobic exercise and T'ai Chi on blood pressure in older people: results of a randomized trial. *J Am Geriatr Soc* **1999**, 47, 277-284, doi:10.1111/j.1532-5415.1999.tb02989.x.
139. Zheng, S.; Kim, C.; Lal, S.; Meier, P.; Sibbritt, D.; Zaslawski, C. The Effects of Twelve Weeks of Tai Chi Practice on Anxiety in Stressed But Healthy People Compared to Exercise and Wait-List Groups-A Randomized Controlled Trial. *J Clin Psychol* **2018**, 74, 83-92, doi:10.1002/jclp.22482.
