# Supplementary material for: Clinical Evidence of Tai Chi Exercise Prescriptions: A Systematic Review
Source: Evid Based Complement Alternat Med. 2021 Mar 10;2021:5558805. doi: 10.1155/2021/5558805 (PMC7972853; doi:10.1155/2021/5558805)
Supplement: Supplementary Materials — Table S1: basic characteristics of the included studies. Table S2: musculoskeletal system or connective tissue diseases. Table S3: circulatory system diseases. Table S4: mental and behavioral disorders. Table S5: nervous system diseases. Table S6: respiratory system diseases. Table S7: endocrine, nutritional, or metabolic diseases. Table S8: neoplasms. Table S9: other disease conditions. Table S10: healthy populations. Figure S1: risk of bias summary. [file 5558805.f1.zip › 5558805.f1/Table S10 Healthy populations(revised version).pdf]

Table S10. Healthy populations (n=44).

Tai Chi styles	Tai Chi forms	Participants	Frequency (weekly)	Time (min)	Duration (week)	Intensity	Conclusion	References
Yang-style Tai Chi (34, 77.3%)	Simplified 24-form Tai Chi (22, 50%)	Elderly women	4	60	24	NR	Positive result	[1]
		Older adults	1	60	12	NR	Positive result	[2]
		Elderly women	5	60	16	NR	Positive result	[3]
		Older adults	3	60	24	NR	Positive result	[4]
		Older adults	2	55	8	NR	Positive result	[5]
		Older adults	3	60	24	NR	Positive result	[6]
		Older adults	7	60	8	NR	Positive result	[7]
		Older adults	2	60	24	NR	Positive result	[8]
		Older adults	2	60	24	NR	Positive result	[9]
		Older adults	3	40	24	NR	Positive result	[10]
		Elderly women	5	60	16	NR	Positive result	[11]
		Elderly women	3	60	48	NR	Positive result	[12]
		Elderly women	3	90	8	NR	Positive result	[13]
		Older adults	4	60	16	NR	Positive result	[14]
		Elderly women	4	60	24	NR	Positive result	[15]
		Elderly women	3	50	12	NR	Positive result	[16]
		Older adults	3	60	24	NR	Positive result	[17]
		Older adults	2	60	24	NR	Positive result	[18]
		Older adults	2	60	16	NR	Positive result	[19]
		Older adults	2	60	24	NR	Positive result	[20]
		Older adults	3	30-40	8	NR	Positive result	[21]
		Healthy but stressed people	2	60	12	NR	Positive result	[22]

	108-form Tai Chi (3, 6.8%)	Middle-aged women	3	60	12	NR	Positive result	[23]
		Older adults	7	30	15	NR	Positive result	[24]
		Older adults	2	60	15	NR	Positive result	[25]
	18-form Tai Chi (2, 4.5%)	Older adults	1	60	24	NR	Positive result	[26]
		Healthy people	2	60	12	NR	Positive result	[27]
	10-form Tai Chi (2, 4.5%)	Older adults	3	60	12	NR	Positive result	[28]
		Elderly women	3	60	12	NR	Positive result	[29]
	Unspecified forms (2, 4.5%)	Older adults	3	60	10	NR	Positive result	[30]
		Postmenopausal women	5	45	48	NR	Positive result	[31]
	5-form Tai Chi (1, 2.3%)	Older adults	1	60	12	NR	Positive result	[32]
	12-form Tai Chi (1, 2.3%)	Elderly women	2	90	16	NR	Positive result	[33]
	13-form Tai Chi (1, 2.3%)	Older adults	2	60	12	Low intensity	Positive result	[34]
Chen-style Tai Chi (3, 6.8%)	7-form Tai Chi (2, 4.5%)	Older adults	3	60	24	Moderate intensity	Positive result	[35]
		Older adults	3	60	20	NR	Positive result	[36]
	12-form Tai Chi (1, 2.3%)	Older adults	3	60	20	NR	Positive result	[37]
Unspecified style (6, 13.6%)	8-form Tai Chi (1, 2.3%)	Older adults	2	60	24	RPE (2-4): 1-10 weeks<br>RPE (5-6): 11-24 weeks	Positive result	[38]
	10-form Tai Chi (1, 2.3%)	Older adults	2	30	12	NR	Positive result	[39]

	12-form Tai Chi (1, 2.3%)	Older adults	3	60	12	NR	Positive result	[40]
	Unspecified forms (3, 6.8%)	Older adults	2	30	24	NR	Positive result	[41]
		Older adults	2	60	24	NR	Positive result	[42]
		Older adults	3	40	16	NR	Positive result	[43]
Multiple styles (1, 2.3%)	Unspecified forms (1, 2.3%)	Older adults	1	60	16	NR	Positive result	[44]

Note: RPE = rating of perceived exertion; NR = not reported.

References:

1. Zhou, J.; Chang, S.; Cong, Y.; Qin, M.; Sun, W.; Lian, J.; Yao, J.; Li, W.; Hong, Y. Effects of 24 weeks of Tai Chi Exercise on Postural Control among Elderly Women. *Res Sports Med* **2015**, 23, 302-314, doi:10.1080/15438627.2015.1040918.
2. Li, Y.; Devault, C.N.; Van Oteghen, S. Effects of extended Tai Chi intervention on balance and selected motor functions of the elderly. *Am J Chin Med* **2007**, 35, 383-391, doi:10.1142/S0192415X07004904.
3. Sun, W.; Ma, X.; Wang, L.; Zhang, C.; Song, Q.; Gu, H.; Mao, D. Effects of Tai Chi Chuan and Brisk Walking Exercise on Balance Ability in Elderly Women: A Randomized Controlled Trial. *Motor Control* **2019**, 23, 100-114, doi:10.1123/mc.2017-0055.
4. Li, F.; Harmer, P.; Fisher, K.J.; McAuley, E.; Chaumeton, N.; Eckstrom, E.; Wilson, N.L. Tai Chi and fall reductions in older adults: a randomized controlled trial. *J Gerontol A Biol Sci Med Sci* **2005**, 60, 187-194, doi:10.1093/gerona/60.2.187.
5. Hosseini, L.; Kargozar, E.; Sharifi, F.; Negarandeh, R.; Memari, A.H.; Navab, E. Tai Chi Chuan can improve balance and reduce fear of falling in community dwelling older adults: a randomized control trial. *J Exerc Rehabil* **2018**, 14, 1024-1031, doi:10.12965/jer.1836488.244.
6. Li, F.; Harmer, P.; Fisher, K.J.; McAuley, E. Tai Chi: improving functional balance and predicting subsequent falls in older persons. *Med Sci Sports Exerc* **2004**, 36, 2046-2052, doi:10.1249/01.mss.0000147590.54632.e7.
7. Zhang, J.G.; Ishikawa-Takata, K.; Yamazaki, H.; Morita, T.; Ohta, T. The effects of Tai Chi Chuan on physiological function and fear of falling in the less robust elderly: an intervention study for preventing falls. *Arch Gerontol Geriatr* **2006**, 42, 107-116, doi:10.1016/j.archger.2005.06.007.
8. Nguyen, M.H.; Kruse, A. A randomized controlled trial of Tai chi for balance, sleep quality and cognitive performance in elderly Vietnamese. *Clin Interv Aging* **2012**, 7, 185-190, doi:10.2147/CIA.S32600.
9. Li, F.Z.; Harmer, P.; McAuley, E.; Duncan, T.E.; Duncan, S.C.; Chaumeton, N.; Fisher, K.J. An evaluation of the effects of Tai Chi exercise on physical function among older persons: A randomized controlled trial. *Ann Behav Med* **2001**, 23, 139-146, doi:10.1207/S15324796ABM2302_9.
10. Shen, C.L.; Williams, J.S.; Chyu, M.C.; Paige, R.L.; Stephens, A.L.; Chauncey, K.B.; Prabhu, F.R.; Ferris, L.T.; Yeh, J.K. Comparison of the effects of Tai Chi and resistance training on bone metabolism in the elderly: a feasibility study. *Am J Chin Med* **2007**, 35, 369-381, doi:10.1142/S0192415X07004898.
11. Sun, W.; Wang, L.; Zhang, C.; Song, Q.; Gu, H.; Mao, D. Detraining effects of regular Tai Chi exercise on postural control ability in older women: A randomized controlled trial. *J Exerc Sci Fit* **2018**, 16, 55-61, doi:10.1016/j.jesf.2018.06.003.
12. Sun, W.; Zhang, C.; Song, Q.; Li, W.; Cong, Y.; Chang, S.; Mao, D.; Hong, Y. Effect of 1-year regular Tai Chi on neuromuscular reaction in elderly women: a randomized controlled study. *Res Sports Med* **2016**, 24, 145-156, doi:10.1080/15438627.2015.1126280.
13. Zou, L.; Wang, C.; Tian, Z.; Wang, H.; Shu, Y. Effect of Yang-Style Tai Chi on Gait Parameters and Musculoskeletal Flexibility in Healthy Chinese Older Women. *Sports (Basel, Switzerland)* **2017**, 5, doi:10.3390/sports5030052.
14. Li, J.X.; Xu, D.Q.; Hong, Y. Effects of 16-week Tai Chi intervention on postural stability and proprioception of knee and ankle in older people. *Age Ageing* **2008**, 37, 575-578, doi:10.1093/ageing/afn109.

15. Chang, S.; Zhou, J.; Hong, Y.; Sun, W.; Cong, Y.; Qin, M.; Lian, J.; Yao, J.; Li, W. Effects of 24-week Tai Chi exercise on the knee and ankle proprioception of older women. *Res Sports Med* **2016**, 24, 84-93, doi:10.1080/15438627.2015.1126281.
16. Pereira, M.M.; Oliveira, R.J.; Silva, M.A.F.; Souza, L.H.R.; Vianna, L.G. Effects of Tai Chi Chuon on knee extensor muscle strength and balance in elderly women. *Braz J Phys Ther* **2008**, 12, 121-126, doi:10.1590/S1413-35552008000200008.
17. Li, F.; Fisher, K.J.; Harmer, P.; McAuley, E. Falls self-efficacy as a mediator of fear of falling in an exercise intervention for older adults. *J Gerontol B Psychol Sci Soc Sci* **2005**, 60, P34-P40, doi:10.1093/geronb/60.1.p34.
18. Li, F.Z.; Harmer, P.; Chaumeton, N.R.; Duncan, T.E.; Duncan, S.C. Tai Chi as a means to enhance self-esteem: A randomized controlled trial. *J Appl Gerontol* **2002**, 21, 70-89, doi:10.1177/073346480202100105.
19. Chen, W.W.; Sun, W.Y. Tai chi chuan, an alternative form of exercise for health promotion and disease prevention for older adults in the community. *International quarterly of community health education* **1996**, 16, 333-339, doi:10.2190/FDPE-VVG2-VNTR-N2DK.
20. Li, F.; Harmer, P.; McAuley, E.; Fisher, K.J.; Duncan, T.E.; Duncan, S.C. Tai Chi, self-efficacy, and physical function in the elderly. *Prev Sci* **2001**, 2, 229-239, doi:10.1023/a:1013614200329.
21. Tajik, A.; Rejeh, N.; Heravi-Karimooi, M.; Samady, K.P.; Tadrisi, S.D.; Watts, T.E.; Griffiths, P.; Vaismoradi, M. The effect of Tai Chi on quality of life in male older people: A randomized controlled clinical trial. *Complement Ther Clin Pract* **2018**, 33, 191-196, doi:10.1016/j.ctcp.2018.10.009.
22. Zheng, S.; Kim, C.; Lal, S.; Meier, P.; Sibbritt, D.; Zaslawski, C. The Effects of Twelve Weeks of Tai Chi Practice on Anxiety in Stressed But Healthy People Compared to Exercise and Wait-List Groups-A Randomized Controlled Trial. *J Clin Psychol* **2018**, 74, 83-92, doi:10.1002/jclp.22482.
23. Thornton, E.W.; Sykes, K.S.; Tang, W.K. Health benefits of Tai Chi exercise: improved balance and blood pressure in middle-aged women. *Health Promot Int* **2004**, 19, 33-38, doi:10.1093/heapro/dah105.
24. Wolf, S.L.; Barnhart, H.X.; Kutner, N.G.; McNeely, E.; Coogler, C.; Xu, T.S.; Clements, S.D.; Connell, B.R.; Fletcher, R.J.; Green, R., et al. Reducing frailty and falls in older persons: An investigation of Tai Chi and computerized balance training. *J Am Geriatr Soc* **1996**, 44, 489-497, doi:10.1111/j.1532-5415.1996.tb01432.x.
25. Wolf, S.L.; Barnhart, H.X.; Ellison, G.L.; Coogler, C.E. The effect of Tai Chi Quan and computerized balance training on postural stability in older subjects. *Phys Ther* **1997**, 77, 371-381, doi:10.1093/ptj/77.4.371.
26. Hwang, H.F.; Chen, S.J.; Lee-Hsieh, J.; Chien, D.K.; Chen, C.Y.; Lin, M.R. Effects of Home-Based Tai Chi and Lower Extremity Training and Self-Practice on Falls and Functional Outcomes in Older Fallers from the Emergency Department-A Randomized Controlled Trial. *J Am Geriatr Soc* **2016**, 64, 518-525, doi:10.1111/jgs.13952.
27. Schitter, A.M.; Nedeljkovic, M.; Ausfeld-Hafter, B.; Fleckenstein, J. Changes in self-reported symptoms of depression and physical well-being in healthy individuals following a Taiji beginner course - Results of a randomized controlled trial. *Brain Behav* **2016**, 6, doi:10.1002/brb3.429.
28. Frye, B.; Scheinthal, S.; Kemarskaya, T.; Pruchno, R. Tai chi and low impact exercise: Effects on the physical functioning and psychological well-being of older people. *J Appl Gerontol* **2007**, 26, 433-453, doi:10.1177/0733464807306915.
29. Audette, J.F.; Jin, Y.S.; Newcomer, R.; Stein, L.; Duncan, G.; Frontera, W.R. Tai Chi versus brisk walking in elderly women. *Age Ageing* **2006**, 35, 388-393,

doi:10.1093/ageing/afl006.
30. Mortazavi, H.; Tabatabaeichehr, M.; Golestani, A.; Armat, M.R.; Yousefi, M.R. The Effect of Tai Chi Exercise on the Risk and Fear of Falling in Older Adults: a Randomized Clinical Trial. *Materia socio-medica* **2018**, 30, 38-42, doi:10.5455/msm.2018.30.38-42.
31. Chan, K.; Qin, L.; Lau, M.; Woo, J.; Au, S.; Choy, W.; Lee, K.; Lee, S. A randomized, prospective study of the effects of Tai Chi Chun exercise on bone mineral density in postmenopausal women. *Arch Phys Med Rehabil* **2004**, 85, 717-722, doi:10.1016/j.apmr.2003.08.091.
32. Holmes, M.L.; Manor, B.; Hsieh, W.; Hu, K.; Lipsitz, L.A.; Li, L. Tai Chi training reduced coupling between respiration and postural control. *Neurosci Lett* **2016**, 610, 60-65, doi:10.1016/j.neulet.2015.10.053.
33. Lu, X.; Hui-Chan, C.W.; Tsang, W.W. Effects of Tai Chi training on arterial compliance and muscle strength in female seniors: a randomized clinical trial. *Eur J Prev Cardiol* **2013**, 20, 238-245, doi:10.1177/2047487311434233.
34. Young, D.R.; Appel, L.J.; Jee, S.; Miller, E.R. The effects of aerobic exercise and T'ai Chi on blood pressure in older people: results of a randomized trial. *J Am Geriatr Soc* **1999**, 47, 277-284, doi:10.1111/j.1532-5415.1999.tb02989.x.
35. Yang, Y.; Verkuilen, J.V.; Rosengren, K.S.; Grubisich, S.A.; Reed, M.R.; Hsiao-Wecksler, E.T. Effect of combined Taiji and Qigong training on balance mechanisms: a randomized controlled trial of older adults. *Med Sci Monit* **2007**, 13, R339-R348.
36. Yang, Y.; Verkuilen, J.; Rosengren, K.S.; Mariani, R.A.; Reed, M.; Grubisich, S.A.; Woods, J.A.; Schlagal, B. Effects of a traditional Taiji/Qigong curriculum on older adults' immune response to influenza vaccine. *Med Sport Sci* **2008**, 52, 64-76, doi:10.1159/000134285.
37. Christou, E.A.; Yang, Y.; Rosengren, K.S. Taiji training improves knee extensor strength and force control in older adults. *J Gerontol A Biol Sci Med Sci* **2003**, 58, 763-766, doi:10.1093/gerona/58.8.m763.
38. Li, F.; Harmer, P.; Fitzgerald, K.; Eckstrom, E.; Akers, L.; Chou, L.S.; Pidgeon, D.; Voit, J.; Winters-Stone, K. Effectiveness of a Therapeutic Tai Ji Quan Intervention vs a Multimodal Exercise Intervention to Prevent Falls Among Older Adults at High Risk of Falling: A Randomized Clinical Trial. *Jama Intern Med* **2018**, 178, 1301-1310, doi:10.1001/jamainternmed.2018.3915.
39. Lelard, T.; Doutrellot, P.L.; David, P.; Ahmaidi, S. Effects of a 12-week Tai Chi Chuan program versus a balance training program on postural control and walking ability in older people. *Arch Phys Med Rehabil* **2010**, 91, 9-14, doi:10.1016/j.apmr.2009.09.014.
40. Kim, H. Effects of Tai Chi Exercise on the Center of Pressure Trace during Obstacle Crossing in Older Adults who are at a Risk of Falling. *JOURNAL OF PHYSICAL THERAPY SCIENCE* **2009**, 21, 49-54, doi:10.1589/jpts.21.49.
41. Wayne, P.M.; Manor, B.; Novak, V.; Costa, M.D.; Hausdorff, J.M.; Goldberger, A.L.; Ahn, A.C.; Yeh, G.Y.; Peng, C.K.; Lough, M., et al. A systems biology approach to studying Tai Chi, physiological complexity and healthy aging: Design and rationale of a pragmatic randomized controlled trial. *Contemp Clin Trials* **2013**, 34, 21-34, doi:10.1016/j.cct.2012.09.006.
42. Li, F.; Fisher, K.J.; Harmer, P.; McAuley, E. Delineating the impact of Tai Chi training on physical function among the elderly. *Am J Prev Med* **2002**, 23, 92-97, doi:10.1016/s0749-3797(02)00479-8.

43. Irwin, M.R.; Olmstead, R. Mitigating cellular inflammation in older adults: a randomized controlled trial of Tai Chi Chih. *Am J Geriatr Psychiatry* **2012**, 20, 764-772, doi:10.1097/JGP.0b013e3182330fd3.
44. Voukelatos, A.; Cumming, R.G.; Lord, S.R.; Rissel, C. A randomized, controlled trial of tai chi for the prevention of falls: the Central Sydney tai chi trial. *J Am Geriatr Soc* **2007**, 55, 1185-1191, doi:10.1111/j.1532-5415.2007.01244.x.
