# Supplementary material for: Clinical Evidence of Tai Chi Exercise Prescriptions: A Systematic Review
Source: Evid Based Complement Alternat Med. 2021 Mar 10;2021:5558805. doi: 10.1155/2021/5558805 (PMC7972853; doi:10.1155/2021/5558805)
Supplement: Supplementary Materials — Table S1: basic characteristics of the included studies. Table S2: musculoskeletal system or connective tissue diseases. Table S3: circulatory system diseases. Table S4: mental and behavioral disorders. Table S5: nervous system diseases. Table S6: respiratory system diseases. Table S7: endocrine, nutritional, or metabolic diseases. Table S8: neoplasms. Table S9: other disease conditions. Table S10: healthy populations. Figure S1: risk of bias summary. [file 5558805.f1.zip › 5558805.f1/Table S2 Musculoskeletal system or connective tissue diseases(revised version).pdf]

Table S2. Musculoskeletal system or connective tissue diseases (n=33).

Tai Chi styles	Tai Chi forms	Participants	Frequency (weekly)	Time (min)	Duration (week)	Intensity	Conclusion	References
Yang-style Tai Chi (21, 63.6%)	Simplified 24-form Tai Chi (7, 21.2%)	Older women with OA	3	60	24	NR	Positive result	[1]
		Older women with OA	3	60	24	NR	Positive result	[2]
		Knee OA patients	3	40	12	NR	Positive result	[3]
		Older women with OA	2-4	40	24	NR	Positive result	[4]
		Retired athlete with NLBP	5	45	24	NR	Positive result	[5]
		Men with osteoporosis	2	45	18	NR	Positive result	[6]
		Women with osteoporosis	3	60	24	NR	Positive result	[7]
	10-form Tai Chi (5, 15.1%)	Knee OA patients	2	60	12	NR	Positive result	[8]
		Knee OA patients	2	60	12	NR	Positive result	[9]
		Patients with FM	2	60	12	NR	Positive result	[10]
		Patients with FM	3	55	12	NR	Positive result	[11]
		Patients with partial ACL injuries	3	60	24	NR	Positive result	[12]
	Unspecified forms (3, 9.1%)	Knee OA patients	2	60	12	NR	Positive result	[13]
		Patients with FM	1-2	60	12/24	NR	Positive result	[14]
		Adults with rheumatoid arthritis	2	60	12	NR	Positive result	[15]

	8-form Tai Chi (2, 6.1%)	Patients with FM	2	90	12	NR	Positive result	[16]
		Older adults with chronic multisite pain	2	60	12	NR	Positive result	[17]
	13-form Tai Chi (2, 6.1%)	Patients chronic non-specific neck pain	1	60-90	12	NR	Positive result	[18]
		Patients chronic non-specific neck pain	1	75-90	12	NR	Positive result	[19]
	12-form Tai Chi (1, 3.1%)	Knee OA patients	2	60	10	NR	Positive result	[20]
	9-form Tai Chi (1, 3.1%)	Older adults with OA	2	60	12	NR	Positive result	[21]
Sun-style Tai Chi (8, 24.2%)	12-form Tai Chi (4, 12.1%)	Elders with knee OA	3	40	20	NR	Positive result	[22]
		Older women with OA	3 (week 1 to week 2)<br>1 (week 3 to week 12)	60	12	NR	Positive result	[23]
		Older women with OA	3 (week 1 to week 2)<br>1 (week 3 to week 12)	60	12	NR	Positive result	[24]
		Knee OA patients	2	60	8	NR	Positive result	[25]
	21-form Tai Chi (2, 6.1%)	Patients with NLBP	2 (week 1 to week 8)<br>1 (week 9 to week 10)	40	10	NR	Positive result	[26]
		Patients with ankylosing spondylitis	2	45	8	NR	Positive result	[27]
	24-form Tai Chi (1, 3.1%)	Elders with knee OA	2	60	12	NR	Positive result	[28]
	31-form Tai Chi (1, 3.1%)	Older women with OA	1	60	24	NR	Positive result	[29]

Chen-style Tai Chi (1, 3.1%)	Unspecified forms (1, 3.1%)	Elders with NLBP	3	60	12	NR	Positive result	[30]
Unspecified style (3, 9.1%)	7-form Tai Chi (1, 3.1%)	Young males with acute LBP	3	60	4	NR	Positive result	[31]
	14-form Tai Chi (1, 3.1%)	Patients with FM	2	60	16	NR	Positive result	[32]
	Unspecified forms (1, 3.1%)	Women with osteoporosis	2 (week 1 to week 4)<br>1 (week 5 to week 36)	60	36	NR	Positive result	[33]

Note: OA = osteoarthritis; NLBP = non-specific chronic lower back pain; LBP = lower back pain; FM = fibromyalgia; ACL = anterior cruciate ligament; NR = not reported.

References:

1. Lu, J.; Huang, L.; Wu, X.; Fu, W.; Liu, Y. Effect of Tai Ji Quan training on self-reported sleep quality in elderly Chinese women with knee osteoarthritis: a randomized controlled trail. *Sleep Med* **2017**, 33, 70-75, doi:10.1016/j.sleep.2016.12.024.
2. Zhu, Q.; Huang, L.; Wu, X.; Wang, L.; Zhang, Y.; Fang, M.; Liu, Y.; Li, J.X. Effects of Tai Ji Quan training on gait kinematics in older Chinese women with knee osteoarthritis: A randomized controlled trial. *J Sport Health Sci* **2016**, 5, 297-303, doi:10.1016/j.jshs.2016.02.003.
3. Brismee, J.M.; Paige, R.L.; Chyu, M.C.; Boatright, J.D.; Hagar, J.M.; McCaleb, J.A.; Quintela, M.M.; Feng, D.; Xu, K.T.; Shen, C.L. Group and home-based tai chi in elderly subjects with knee osteoarthritis: a randomized controlled trial. *Clin Rehabil* **2007**, 21, 99-111, doi:10.1177/0269215506070505.
4. Ni, G.X.; Song, L.; Yu, B.; Huang, C.H.; Lin, J.H. Tai chi improves physical function in older Chinese women with knee osteoarthritis. *J Clin Rheumatol* **2010**, 16, 64-67, doi:10.1097/RHU.0b013e3181cf344f.
5. Wu, W.; Muheremu, A.; Chen, C.; Liu, W.; Sun, L. Effectiveness of Tai Chi Practice for Non-Specific Chronic Low Back Pain on Retired Athletes: A Randomized Controlled Study. *J Musculoskelet Pain* **2013**, 21, 37-45, doi:10.3109/10582452.2013.763394.
6. Maciaszek, J.; Osinski, W.; Szeklicki, R.; Stemplewski, R. Effect of Tai Chi on body balance: randomized controlled trial in men with osteopenia or osteoporosis. *Am J Chin Med* **2007**, 35, 1-9, doi:10.1142/S0192415X07004564.
7. Chyu, M.C.; James, C.R.; Sawyer, S.F.; Brismee, J.M.; Xu, K.T.; Poklikuha, G.; Dunn, D.M.; Shen, C.L. Effects of tai chi exercise on posturography, gait, physical function and quality of life in postmenopausal women with osteopaenia: a randomized clinical study. *Clin Rehabil* **2010**, 24, 1080-1090, doi:10.1177/0269215510375902.
8. Schmid, A.; McAlindon, T.; Schmid, C.H.; Wang, C. The Influence of Tai Chi Exercise on Proprioception in Patients with Knee Osteoarthritis: Results from a Pilot Randomized Controlled Trial. *International journal of integrative medicine* **2013**, 1, doi:10.5772/57137.
9. Wang, C.; Schmid, C.H.; Hibberd, P.L.; Kalish, R.; Roubenoff, R.; Rones, R.; McAlindon, T. Tai Chi is effective in treating knee osteoarthritis: a randomized controlled trial. *Arthritis Rheum* **2009**, 61, 1545-1553, doi:10.1002/art.24832.
10. Wang, C.; Schmid, C.H.; Rones, R.; Kalish, R.; Yinh, J.; Goldenberg, D.L.; Lee, Y.; McAlindon, T. A randomized trial of tai chi for fibromyalgia. *N Engl J Med* **2010**, 363, 743-754, doi:10.1056/NEJMoa0912611.
11. Wong, A.; Figueroa, A.; Sanchez-Gonzalez, M.A.; Son, W.M.; Chernykh, O.; Park, S.Y. Effectiveness of Tai Chi on Cardiac Autonomic Function and Symptomatology in Women With Fibromyalgia: A Randomized Controlled Trial. *J Aging Phys Act* **2018**, 26, 214-221, doi:10.1123/japa.2017-0038.
12. Buyukturan, O.; Buyukturan, B.; Kurt, E.E.; Yetis, M. Effects of Tai Chi on partial anterior cruciate ligament injury: A single-blind, randomized-controlled trial. *Turk J Phys Med Rehabil* **2019**, 65, 160-168, doi:10.5606/tftrd.2019.2798.
13. Wang, C.; Schmid, C.H.; Iversen, M.D.; Harvey, W.F.; Fielding, R.A.; Driban, J.B.; Price, L.L.; Wong, J.B.; Reid, K.F.; Rones, R., et al. Comparative Effectiveness of Tai Chi Versus Physical Therapy for Knee Osteoarthritis: A Randomized Trial. *Ann Intern Med* **2016**, 165, 77-86, doi:10.7326/M15-2143.
14. Wang, C.; Schmid, C.H.; Fielding, R.A.; Harvey, W.F.; Reid, K.F.; Price, L.L.; Driban, J.B.; Kalish, R.; Rones, R.; McAlindon, T. Effect of tai chi versus aerobic exercise for fibromyalgia: comparative effectiveness randomized controlled trial. *BMJ* **2018**, 360, k851, doi:10.1136/bmj.k851.

15. Wang, C. Tai Chi improves pain and functional status in adults with rheumatoid arthritis: results of a pilot single-blinded randomized controlled trial. *Med Sport Sci* **2008**, 52, 218-229, doi:10.1159/000134302.
16. Jones, K.D.; Sherman, C.A.; Mist, S.D.; Carson, J.W.; Bennett, R.M.; Li, F. A randomized controlled trial of 8-form Tai chi improves symptoms and functional mobility in fibromyalgia patients. *Clin Rheumatol* **2012**, 31, 1205-1214, doi:10.1007/s10067-012-1996-2.
17. You, T.; Ogawa, E.F.; Thapa, S.; Cai, Y.; Zhang, H.; Nagae, S.; Yeh, G.Y.; Wayne, P.M.; Shi, L.; Leveille, S.G. Tai Chi for older adults with chronic multisite pain: a randomized controlled pilot study. *Aging Clin Exp Res* **2018**, 30, 1335-1343, doi:10.1007/s40520-018-0922-0.
18. Lauche, R.; Wayne, P.M.; Fehr, J.; Stumpe, C.; Dobos, G.; Cramer, H. Does Postural Awareness Contribute to Exercise-Induced Improvements in Neck Pain Intensity? A Secondary Analysis of a Randomized Controlled Trial Evaluating Tai Chi and Neck Exercises. *Spine (Phila Pa 1976)* **2017**, 42, 1195-1200, doi:10.1097/BRS.0000000000002078.
19. Lauche, R.; Stumpe, C.; Fehr, J.; Cramer, H.; Cheng, Y.W.; Wayne, P.M.; Rampp, T.; Langhorst, J.; Dobos, G. The Effects of Tai Chi and Neck Exercises in the Treatment of Chronic Nonspecific Neck Pain: A Randomized Controlled Trial. *J Pain* **2016**, 17, 1013-1027, doi:10.1016/j.jpain.2016.06.004.
20. Wortley, M.; Zhang, S.; Paquette, M.; Byrd, E.; Baumgartner, L.; Klipple, G.; Krusenklaus, J.; Brown, L. Effects of resistance and Tai Ji training on mobility and symptoms in knee osteoarthritis patients. *J Sport Health Sci* **2013**, 2, 209-214, doi:10.1016/j.jshs.2013.01.001.
21. Hartman, C.A.; Manos, T.M.; Winter, C.; Hartman, D.M.; Li, B.; Smith, J.C. Effects of T'ai Chi training on function and quality of life indicators in older adults with osteoarthritis. *J Am Geriatr Soc* **2000**, 48, 1553-1559, doi:10.1111/j.1532-5415.2000.tb03863.x.
22. Tsai, P.; Chang, J.Y.; Beck, C.; Kuo, Y.; Keefe, F.J. A Pilot Cluster-Randomized Trial of a 20-Week Tai Chi Program in Elders With Cognitive Impairment and Osteoarthritic Knee: Effects on Pain and Other Health Outcomes. *J Pain Symptom Manag* **2013**, 45, 660-669, doi:10.1016/j.jpainsymman.2012.04.009.
23. Song, R.; Lee, E.O.; Lam, P.; Bae, S.C. Effects of a Sun-style Tai Chi exercise on arthritic symptoms, motivation and the performance of health behaviors in women with osteoarthritis. *Taehan Kanho Hakhoe Chi* **2007**, 37, 249-256, doi:10.4040/jkan.2007.37.2.249.
24. Song, R.; Lee, E.O.; Lam, P.; Bae, S.C. Effects of tai chi exercise on pain, balance, muscle strength, and perceived difficulties in physical functioning in older women with osteoarthritis: a randomized clinical trial. *J Rheumatol* **2003**, 30, 2039-2044.
25. Callahan, L.F.; Cleveland, R.J.; Altpeter, M.; Hackney, B. Evaluation of Tai Chi Program Effectiveness for People with Arthritis in the Community: A Randomized Controlled Trial. *J Aging Phys Activ* **2016**, 24, 101-110, doi:10.1123/japa.2014-0211.
26. Hall, A.M.; Maher, C.G.; Lam, P.; Ferreira, M.; Latimer, J. Tai chi exercise for treatment of pain and disability in people with persistent low back pain: a randomized controlled trial. *Arthritis Care Res (Hoboken)* **2011**, 63, 1576-1583, doi:10.1002/acr.20594.
27. Taylor-Piliae, R.E.; Hoke, T.M.; Hepworth, J.T.; Latt, L.D.; Najafi, B.; Coull, B.M. Effect of Tai Chi on physical function, fall rates and quality of life among older stroke survivors. *Arch Phys Med Rehabil* **2014**, 95, 816-824, doi:10.1016/j.apmr.2014.01.001.
28. Fransen, M.; Nairn, L.; Winstanley, J.; Lam, P.; Edmonds, J. Physical activity for osteoarthritis management: A randomized controlled clinical trial evaluating hydrotherapy or Tai Chi classes. *ARTHRITIS & RHEUMATISM-ARTHRITIS CARE & RESEARCH* **2007**, 57, 407-414, doi:10.1002/art.22621.

29. Song, R.; Roberts, B.L.; Lee, E.O.; Lam, P.; Bae, S.C. A randomized study of the effects of t'ai chi on muscle strength, bone mineral density, and fear of falling in women with osteoarthritis. *J Altern Complement Med* **2010**, 16, 227-233, doi:10.1089/acm.2009.0165.
30. Zou, L.; Zhang, Y.; Liu, Y.; Tian, X.; Xiao, T.; Liu, X.; Yeung, A.S.; Liu, J.; Wang, X.; Yang, Q. The Effects of Tai Chi Chuan Versus Core Stability Training on Lower-Limb Neuromuscular Function in Aging Individuals with Non-Specific Chronic Lower Back Pain. *Medicina (Kaunas)* **2019**, 55, doi:10.3390/medicina55030060.
31. Cho, Y. Effects of tai chi on pain and muscle activity in young males with acute low back pain. *J Phys Ther Sci* **2014**, 26, 679-681, doi:10.1589/jpts.26.679.
32. Maddali, B.S.; Paoletti, G.; Cala, M.; Del, R.A.; El, A.K.; Mikhaylova, S. Efficacy of rehabilitation with Tai Ji Quan in an Italian cohort of patients with Fibromyalgia Syndrome. *Complement Ther Clin Pract* **2016**, 24, 109-115, doi:10.1016/j.ctcp.2016.05.010.
33. Wayne, P.M.; Kiel, D.P.; Buring, J.E.; Connors, E.M.; Bonato, P.; Yeh, G.Y.; Cohen, C.J.; Mancinelli, C.; Davis, R.B. Impact of Tai Chi exercise on multiple fracture-related risk factors in post-menopausal osteopenic women: a pilot pragmatic, randomized trial. *BMC Complement Altern Med* **2012**, 12, 7, doi:10.1186/1472-6882-12-7.
