# Supplementary material for: Clinical Evidence of Tai Chi Exercise Prescriptions: A Systematic Review
Source: Evid Based Complement Alternat Med. 2021 Mar 10;2021:5558805. doi: 10.1155/2021/5558805 (PMC7972853; doi:10.1155/2021/5558805)
Supplement: Supplementary Materials — Table S1: basic characteristics of the included studies. Table S2: musculoskeletal system or connective tissue diseases. Table S3: circulatory system diseases. Table S4: mental and behavioral disorders. Table S5: nervous system diseases. Table S6: respiratory system diseases. Table S7: endocrine, nutritional, or metabolic diseases. Table S8: neoplasms. Table S9: other disease conditions. Table S10: healthy populations. Figure S1: risk of bias summary. [file 5558805.f1.zip › 5558805.f1/Table S3 Circulatory system diseases(revised version).pdf]

Table S3. Circulatory system diseases (n=22).

Tai Chi styles	Tai Chi forms	Participants	Frequency (weekly)	Time (min)	Duration (week)	Intensity	Conclusion	References
Yang-style Tai Chi (17, 77.3%)	Simplified 24-form Tai Chi (5, 22.7%)	Older Stroke Survivors	3	60	12	NR	Positive result	[1]
		Hypertension in young and middle-aged in-service staff	7	40-90	12	50%–60% VO_{2max}; 70%–80% HR_{max}	Positive result	[2]
		Adults with hypertension	2	60	12	Moderate intensity	Positive result	[3]
		Patients with myocardial infarction	3	60	12	NR	Positive result	[4]
		Older adults with hypertension	3-5	60	24	NR	Positive result	[5]
	5-form Tai Chi (5, 22.7%)	Patients with HF	2	60	12	NR	Positive result	[6]
		Patients with HF	2	60	12	NR	Positive result	[7]
		Patients with HF	2	60	12	NR	Positive result	[8]
		Patients with HF	2	60	12	NR	Positive result	[9]
		Patients with HF	2	60	12	NR	Positive result	[10]
	108-form Tai Chi (2, 9.1%)	Patients with hypertension	3	50	12	64% HR_{max}	Positive result	[11]
		Patients With CHD	3	115	24	NR	Positive result	[12]
	Unspecified forms (2, 9.1%)	Stroke patients	1	50	12	NR	Positive result	[13]
		Patients with HF	2	60	12	NR	Positive result	[14]
	12-form Tai Chi (1,4.5%)	Stroke Survivors	2	60	12	NR	Positive result	[15]
	10-form Tai Chi (1, 4.5%)	Stroke patients	2	60	6	NR	Positive result	[16]
	8-form Tai Chi (1, 4.5%)	Patients With CHD	1	60	48	NR	Positive result	[17]

Sun-style Tai Chi (1, 4.5%)	12-form Tai Chi (1, 4.5%)	Stroke patients	1	60	12	NR	Positive result	[18]
Wu-style Tai Chi (1, 4.5%)	Unspecified forms (1, 4.5%)	Patients with HF	2	55	16	NR	Positive result	[19]
Unspecified style (3, 13.6%)	Unspecified forms (1, 4.5%)	Patients With CHD	2	60	12	NR	Positive result	[20]
	12-form Tai Chi (1, 4.5%)	Patients With CHD	2	60	12	NR	Positive result	[21]
	Tai Chi (Yunshou movement) (1, 4.5%)	Stroke patients	5	60	12	NR	Positive result	[22]

Note: HR_{max} = maximum heart rate; VO_{2max} = maximum oxygen uptake; HF = heart failure; CHD = coronary heart disease; NR = not reported.

References:

1. Taylor-Piliae, R.E.; Hoke, T.M.; Hepworth, J.T.; Latt, L.D.; Najafi, B.; Coull, B.M. Effect of Tai Chi on physical function, fall rates and quality of life among older stroke survivors. *Arch Phys Med Rehabil* **2014**, 95, 816-824, doi:10.1016/j.apmr.2014.01.001.
2. Shou, X.L.; Wang, L.; Jin, X.Q.; Zhu, L.Y.; Ren, A.H.; Wang, Q.N. Effect of T'ai Chi Exercise on Hypertension in Young and Middle-Aged In-Service Staff. *J Altern Complement Med* **2019**, 25, 73-78, doi:10.1089/acm.2018.0011.
3. Chan, A.; Chair, S.Y.; Lee, D.; Leung, D.; Sit, J.; Cheng, H.Y.; Taylor-Piliae, R.E. Tai Chi exercise is more effective than brisk walking in reducing cardiovascular disease risk factors among adults with hypertension: A randomised controlled trial. *Int J Nurs Stud* **2018**, 88, 44-52, doi:10.1016/j.ijnurstu.2018.08.009.
4. Nery, R.M.; Zanini, M.; de Lima, J.B.; Buhler, R.P.; Da, S.A.; Stein, R. Tai Chi Chuan improves functional capacity after myocardial infarction: A randomized clinical trial. *Am Heart J* **2015**, 169, 854-860, doi:10.1016/j.ahj.2015.01.017.
5. Ma, C.; Zhou, W.; Tang, Q.; Huang, S. The impact of group-based Tai chi on health-status outcomes among community-dwelling older adults with hypertension. *Heart Lung* **2018**, 47, 337-344, doi:10.1016/j.hrtlng.2018.04.007.
6. Yeh, G.Y.; Wood, M.J.; Lorell, B.H.; Stevenson, L.W.; Eisenberg, D.M.; Wayne, P.M.; Goldberger, A.L.; Davis, R.B.; Phillips, R.S. Effects of tai chi mind-body movement therapy on functional status and exercise capacity in patients with chronic heart failure: a randomized controlled trial. *Am J Med* **2004**, 117, 541-548, doi:10.1016/j.amjmed.2004.04.016.
7. Yeh, G.Y.; Mietus, J.E.; Peng, C.K.; Phillips, R.S.; Davis, R.B.; Wayne, P.M.; Goldberger, A.L.; Thomas, R.J. Enhancement of sleep stability with Tai Chi exercise in chronic heart failure: preliminary findings using an ECG-based spectrogram method. *Sleep Med* **2008**, 9, 527-536, doi:10.1016/j.sleep.2007.06.003.
8. Yeh, G.Y.; Wayne, P.M.; Phillips, R.S. T'ai Chi exercise in patients with chronic heart failure. *Med Sport Sci* **2008**, 52, 195-208, doi:10.1159/000134300.
9. Yeh, G.Y.; McCarthy, E.P.; Wayne, P.M.; Stevenson, L.W.; Wood, M.J.; Forman, D.; Davis, R.B.; Phillips, R.S. Tai chi exercise in patients with chronic heart failure: a randomized clinical trial. *Arch Intern Med* **2011**, 171, 750-757, doi:10.1001/archinternmed.2011.150.
10. Yeh, G.Y.; Wood, M.J.; Wayne, P.M.; Quilty, M.T.; Stevenson, L.W.; Davis, R.B.; Phillips, R.S.; Forman, D.E. Tai chi in patients with heart failure with preserved ejection fraction. *Congest Heart Fail* **2013**, 19, 77-84, doi:10.1111/chf.12005.
11. Tsai, J.C.; Wang, W.H.; Chan, P.; Lin, L.J.; Wang, C.H.; Tomlinson, B.; Hsieh, M.H.; Yang, H.Y.; Liu, J.C. The beneficial effects of Tai Chi Chuan on blood pressure and lipid profile and anxiety status in a randomized controlled trial. *J Altern Complement Med* **2003**, 9, 747-754, doi:10.1089/107555303322524599.
12. Chang, R.Y.; Koo, M.; Kan, C.B.; Yu, Z.R.; Chu, I.T.; Hsu, C.T.; Chen, C.Y. Effects of tai chi rehabilitation on heart rate responses in patients with coronary artery disease. *Am J Chinese Med* **2010**, 38, 461-472, doi:10.1142/S0192415X10007981.
13. Wang, W.; Sawada, M.; Noriyama, Y.; Arita, K.; Ota, T.; Sadamatsu, M.; Kiyotou, R.; Hirai, M.; Kishimoto, T. Tai Chi exercise versus rehabilitation for the elderly with cerebral vascular disorder: a single-blinded randomized controlled trial. *Psychogeriatrics* **2010**, 10, 160-166, doi:10.1111/j.1479-8301.2010.00334.x.
14. Redwine, L.S.; Tsuang, M.; Rusiewicz, A.; Pandzic, I.; Cammarata, S.; Rutledge, T.; Hong, S.; Linke, S.; Mills, P.J. A pilot study exploring the effects of a 12-week t'ai chi intervention on somatic symptoms of depression in patients with heart failure. *J Altern Complement Med* **2012**, 18, 744-748, doi:10.1089/acm.2011.0314.

15. Chan, W.N.; Tsang, W.W. Effect of Tai Chi Training on Dual-Tasking Performance That Involves Stepping Down among Stroke Survivors: A Pilot Study. *Evid Based Complement Alternat Med* **2017**, 2017, 9134173, doi:10.1155/2017/9134173.
16. Kim, H.; Kim, Y.L.; Lee, S.M. Effects of therapeutic Tai Chi on balance, gait, and quality of life in chronic stroke patients. *Int J Rehabil Res* **2015**, 38, 156-161, doi:10.1097/MRR.0000000000000103.
17. Sato, S.; Makita, S.; Uchida, R.; Ishihara, S.; Masuda, M. Effect of Tai Chi Training on Baroreflex Sensitivity and Heart Rate Variability in Patients with Coronary Heart Disease. *Int Heart J* **2010**, 51, 238-241, doi:10.1536/ihj.51.238.
18. Au-Yeung, S.S.; Hui-Chan, C.W.; Tang, J.C. Short-form Tai Chi improves standing balance of people with chronic stroke. *Neurorehabil Neural Repair* **2009**, 23, 515-522, doi:10.1177/1545968308326425.
19. Barrow, D.E.; Bedford, A.; Ives, G.; O'Toole, L.; Channer, K.S. An evaluation of the effects of Tai Chi Chuan and Chi Kung training in patients with symptomatic heart failure: a randomised controlled pilot study. *Postgrad Med J* **2007**, 83, 717-721, doi:10.1136/pgmj.2007.061267.
20. Salmoirago-Blotcher, E.; Wayne, P.M.; Dunsiger, S.; Krol, J.; Breault, C.; Bock, B.C.; Wu, W.; Yeh, G.Y. Tai Chi Is a Promising Exercise Option for Patients with Coronary Heart Disease Declining Cardiac Rehabilitation. *J Am Heart Assoc* **2017**, 6, doi:10.1161/JAHA.117.006603.
21. Liu, J.; Li, B.; Shnider, R. EFFECTS OF TAI CHI TRAINING ON IMPROVING PHYSICAL FUNCTION IN PATIENTS WITH CORONARY HEART DISEASES. *J Exerc Sci Fit* **2010**, 8, 78-84, doi:10.1016/S1728-869X(10)60012-3.
22. Xie, G.; Rao, T.; Lin, L.; Lin, Z.; Xiao, T.; Yang, M.; Xu, Y.; Fan, J.; Lin, S.; Wu, J., et al. Effects of Tai Chi Yunshou exercise on community-based stroke patients: a cluster randomized controlled trial. *Eur Rev Aging Phys a* **2018**, 15, doi:10.1186/s11556-018-0206-x.
