# Supplementary material for: Clinical Evidence of Tai Chi Exercise Prescriptions: A Systematic Review
Source: Evid Based Complement Alternat Med. 2021 Mar 10;2021:5558805. doi: 10.1155/2021/5558805 (PMC7972853; doi:10.1155/2021/5558805)
Supplement: Supplementary Materials — Table S1: basic characteristics of the included studies. Table S2: musculoskeletal system or connective tissue diseases. Table S3: circulatory system diseases. Table S4: mental and behavioral disorders. Table S5: nervous system diseases. Table S6: respiratory system diseases. Table S7: endocrine, nutritional, or metabolic diseases. Table S8: neoplasms. Table S9: other disease conditions. Table S10: healthy populations. Figure S1: risk of bias summary. [file 5558805.f1.zip › 5558805.f1/Table S4 Mental and behavioral disorders(revised version).pdf]

Table S4. Mental and behavioral disorders (n=12).

Tai Chi styles	Tai Chi forms	Participants	Frequency (weekly)	Time (min)	Duration (week)	Intensity	Conclusion	References
Yang-style Tai Chi (7, 58.3%)	Simplified 24-form Tai Chi (4, 33.3%)	Subthreshold depression adolescents	2	90	8	NR	Positive result	[1]
		Older persons with mild to moderate depression	3	50	12	Moderate intensity	Positive result	[2]
		Older individuals with depression	3	60	24	NR	Positive result	[3]
		Elders with mild cognitive impairment	3	30	48	NR	Positive result	[4]
	108-form Tai Chi (1, 8.3%)	Chinese Americans with major depression	2	60	12	NR	Positive result	[5]
	8-form Tai Chi (1, 8.3%)	Children and adolescents with intellectual disability	2	60	12	NR	Positive result	[6]
	Unspecified forms (1, 8.3%)	Elders with mild cognitive impairment	2	60	24	NR	Positive result	[7]
Sun-style Tai Chi (1, 8.3%)	12-form Tai Chi (1, 8.3%)	Elders with mild cognitive impairment	3	40	20	NR	Positive result	[8]
Wu-style Tai Chi (1, 8.3%)	22-form Tai Chi (1, 8.3%)	Patients with chronic schizophrenia	3	60	12	50%–60% VO_{2max}	Positive result	[9]
Unspecified style (3, 25%)	Unspecified forms (2, 16.7%)	Older adults with major depression	1	120	10	NR	Positive result	[10]
		Obese adults with depression	3	60-90	12	NR	Positive result	[11]
	10-form Tai Chi (1, 8.3%)	Elders with mild cognitive impairment	3	50	12	NR	Positive result	[12]

Note: VO_{2max} = maximum oxygen uptake; NR = not reported.

References:

1. Zhang, J.; Qin, S.; Zhou, Y.; Meng, L.; Su, H.; Zhao, S. A randomized controlled trial of mindfulness-based Tai Chi Chuan for subthreshold depression adolescents. *Neuropsychiatr Dis Treat* **2018**, 14, 2313-2321, doi:10.2147/NDT.S173255.
2. Liao, S.J.; Chong, M.C.; Tan, M.P.; Chua, Y.P. Tai Chi with music improves quality of life among community-dwelling older persons with mild to moderate depressive symptoms: A cluster randomized controlled trial. *Geriatr Nurs* **2019**, 40, 154-159, doi:10.1016/j.gerinurse.2018.08.001.
3. Liu, J.; Xie, H.; Liu, M.; Wang, Z.; Zou, L.; Yeung, A.S.; Hui, S.S.; Yang, Q. The Effects of Tai Chi on Heart Rate Variability in Older Chinese Individuals with Depression. *Int J Environ Res Public Health* **2018**, 15, doi:10.3390/ijerph15122771.
4. Lam, L.C.; Chau, R.C.; Wong, B.M.; Fung, A.W.; Tam, C.W.; Leung, G.T.; Kwok, T.C.; Leung, T.Y.; Ng, S.P.; Chan, W.M. A 1-year randomized controlled trial comparing mind body exercise (Tai Chi) with stretching and toning exercise on cognitive function in older Chinese adults at risk of cognitive decline. *J Am Med Dir Assoc* **2012**, 13, 515-568, doi:10.1016/j.jamda.2012.03.008.
5. Yeung, A.; Lepoutre, V.; Wayne, P.; Yeh, G.; Slipp, L.E.; Fava, M.; Denninger, J.W.; Benson, H.; Fricchione, G.L. Tai chi treatment for depression in Chinese Americans: a pilot study. *Am J Phys Med Rehabil* **2012**, 91, 863-870, doi:10.1097/PHM.0b013e31825f1a67.
6. Kong, Z.; Sze, T.M.; Yu, J.J.; Loprinzi, P.D.; Xiao, T.; Yeung, A.S.; Li, C.; Zhang, H.; Zou, L. Tai Chi as an Alternative Exercise to Improve Physical Fitness for Children and Adolescents with Intellectual Disability. *Int J Environ Res Public Health* **2019**, 16, doi:10.3390/ijerph16071152.
7. Kasai, J.Y.T.; Busse, A.L.; Magaldi, R.M.; Soci, M.A.; Rosa, P.D.M.; Curiati, J.A.E.; Jacob Filho, W. Effects of Tai Chi Chuan on cognition of elderly women with mild cognitive impairment. *Einstein (Sao Paulo, Brazil)* **2010**, 8, 40-45, doi:10.1590/S1679-45082010AO1470.
8. Tsai, P.F.; Chang, J.Y.; Beck, C.; Kuo, Y.F.; Keefe, F.J.; Rosengren, K. A supplemental report to a randomized cluster trial of a 20-week Sun-style Tai Chi for osteoarthritic knee pain in elders with cognitive impairment. *Complement Ther Med* **2015**, 23, 570-576, doi:10.1016/j.ctim.2015.06.001.
9. Ho, R.T.; Fong, T.C.; Wan, A.H.; Au-Yeung, F.S.; Wong, C.P.; Ng, W.Y.; Cheung, I.K.; Lo, P.H.; Ng, S.M.; Chan, C.L., et al. A randomized controlled trial on the psychophysiological effects of physical exercise and Tai-chi in patients with chronic schizophrenia. *Schizophr Res* **2016**, 171, 42-49, doi:10.1016/j.schres.2016.01.038.
10. Lavretsky, H.; Alstein, L.L.; Olmstead, R.E.; Ercoli, L.M.; Riparetti-Brown, M.; Cyr, N.S.; Irwin, M.R. Complementary use of tai chi chih augments escitalopram treatment of geriatric depression: a randomized controlled trial. *Am J Geriatr Psychiatry* **2011**, 19, 839-850, doi:10.1097/JGP.0b013e31820ee9ef.
11. Liu, X.; Vitetta, L.; Kostner, K.; Crompton, D.; Williams, G.; Brown, W.J.; Lopez, A.; Xue, C.C.; Oei, T.P.; Byrne, G., et al. The effects of tai chi in centrally obese adults with depression symptoms. *Evid Based Complement Alternat Med* **2015**, 2015, 879712, doi:10.1155/2015/879712.
12. Sungkarat, S.; Boripuntakul, S.; Chattipakorn, N.; Watcharasaksilp, K.; Lord, S.R. Effects of Tai Chi on Cognition and Fall Risk in Older Adults with Mild Cognitive Impairment: A Randomized Controlled Trial. *J Am Geriatr Soc* **2017**, 65, 721-727, doi:10.1111/jgs.14594.
