# Supplementary material for: Clinical Evidence of Tai Chi Exercise Prescriptions: A Systematic Review
Source: Evid Based Complement Alternat Med. 2021 Mar 10;2021:5558805. doi: 10.1155/2021/5558805 (PMC7972853; doi:10.1155/2021/5558805)
Supplement: Supplementary Materials — Table S1: basic characteristics of the included studies. Table S2: musculoskeletal system or connective tissue diseases. Table S3: circulatory system diseases. Table S4: mental and behavioral disorders. Table S5: nervous system diseases. Table S6: respiratory system diseases. Table S7: endocrine, nutritional, or metabolic diseases. Table S8: neoplasms. Table S9: other disease conditions. Table S10: healthy populations. Figure S1: risk of bias summary. [file 5558805.f1.zip › 5558805.f1/Table S5 Nervous system diseases(revised version).pdf]

Table S5. Nervous system diseases (n=11).

Tai Chi styles	Tai Chi forms	Participants	Frequency (weekly)	Time (min)	Duration (week)	Intensity	Conclusion	References
Yang-style Tai Chi (7, 63.6%)	Simplified 24-form Tai Chi (2, 18.2%)	Patients tension-type headaches	2	60	15	NR	Positive result	[1]
		Elderly men with dizziness	2	45	18	NR	Positive result	[2]
	6-form Tai Chi (2, 18.2%)	Patients with PD	2	60	24	NR	Positive result	[3]
		Patients with PD	2	60	24	NR	Positive result	[4]
	8-form Tai Chi (1, 9.1%)	Older adults with sleep disorder	3	60	24	NR	Positive result	[5]
	10-form Tai Chi (1, 9.1%)	Patients with dementia	2	60	16	NR	Positive result	[6]
	Unspecified forms (1, 9.1%)	Patients with PD	2	60	13	NR	Positive result	[7]
Sun-style Tai Chi (1, 9.1%)	12-form Tai Chi (1, 9.1%)	Patients with PD	3	60	8	NR	Positive result	[8]
Chen-style Tai Chi (1, 9.1%)	5-form Tai Chi (1, 9.1%)	Patients with dementia	2	45	20	NR	Positive result	[9]
Unspecified style (2, 18.2%)	10-form Tai Chi (2, 18.2%)	Patients with PD	3	60	12	RPE (11-15)	Positive result	[10]
		Patients with PD	3	60	12	RPE (11-15)	Positive result	[11]

Note: PD = Parkinson disease; RPE = rating of perceived exertion; NR = not reported.

References:

1. Abbott, R.B.; Hui, K.K.; Hays, R.D.; Li, M.D.; Pan, T. A randomized controlled trial of tai chi for tension headaches. *Evid Based Complement Alternat Med* **2007**, 4, 107-113, doi:10.1093/ecam/nel050.
2. Maciaszek, J.; Osinski, W. Effect of Tai Chi on body balance: randomized controlled trial in elderly men with dizziness. *Am J Chin Med* **2012**, 40, 245-253, doi:10.1142/S0192415X1250019X.
3. Li, F.; Harmer, P.; Liu, Y.; Eckstrom, E.; Fitzgerald, K.; Stock, R.; Chou, L. A randomized controlled trial of patient-reported outcomes with tai chi exercise in Parkinson's disease. *Movement Disord* **2014**, 29, 539-545, doi:10.1002/mds.25787.
4. Li, F.; Harmer, P.; Fitzgerald, K.; Eckstrom, E.; Stock, R.; Galver, J.; Maddalozzo, G.; Batya, S.S. Tai chi and postural stability in patients with Parkinson's disease. *N Engl J Med* **2012**, 366, 511-519, doi:10.1056/NEJMoa1107911.
5. Li, F.; Fisher, K.J.; Harmer, P.; Irbe, D.; Tearse, R.G.; Weimer, C. Tai chi and self-rated quality of sleep and daytime sleepiness in older adults: a randomized controlled trial. *J Am Geriatr Soc* **2004**, 52, 892-900, doi:10.1111/j.1532-5415.2004.52255.x.
6. Liu, J.; Kwan, R.; Lai, C.K.; Hill, K.D. A simplified 10-step Tai-chi programme to enable people with dementia to improve their motor performance: a feasibility study. *Clin Rehabil* **2018**, 32, 1609-1623, doi:10.1177/0269215518786530.
7. Hackney, M.E.; Earhart, G.M. Tai Chi improves balance and mobility in people with Parkinson disease. *Gait Posture* **2008**, 28, 456-460, doi:10.1016/j.gaitpost.2008.02.005.
8. Cheon, S.; Chae, B.; Sung, H.; Lee, G.C.; Kim, J.W. The Efficacy of Exercise Programs for Parkinson's Disease: Tai Chi versus Combined Exercise. *J Clin Neurol* **2013**, 9, 237-243, doi:10.3988/jcn.2013.9.4.237.
9. Nyman, S.R.; Ingram, W.; Sanders, J.; Thomas, P.W.; Thomas, S.; Vassallo, M.; Raftery, J.; Bibi, I.; Barrado-Martin, Y. Randomised Controlled Trial of The Effect of Tai Chi on Postural Balance of People with Dementia. *Clin Interv Aging* **2019**, 14, 2017-2029, doi:10.2147/CIA.S228931.
10. Choi, H.J. Effects of therapeutic Tai chi on functional fitness and activities of daily living in patients with Parkinson disease. *J Exerc Rehabil* **2016**, 12, 499-503, doi:10.12965/jer.1632654.327.
11. Choi, H.J.; Garber, C.E.; Jun, T.W.; Jin, Y.S.; Chung, S.J.; Kang, H.J. Therapeutic effects of tai chi in patients with Parkinson's disease. *ISRN Neurol* **2013**, 2013, 548240, doi:10.1155/2013/548240.
