# Supplementary material for: Clinical Evidence of Tai Chi Exercise Prescriptions: A Systematic Review
Source: Evid Based Complement Alternat Med. 2021 Mar 10;2021:5558805. doi: 10.1155/2021/5558805 (PMC7972853; doi:10.1155/2021/5558805)
Supplement: Supplementary Materials — Table S1: basic characteristics of the included studies. Table S2: musculoskeletal system or connective tissue diseases. Table S3: circulatory system diseases. Table S4: mental and behavioral disorders. Table S5: nervous system diseases. Table S6: respiratory system diseases. Table S7: endocrine, nutritional, or metabolic diseases. Table S8: neoplasms. Table S9: other disease conditions. Table S10: healthy populations. Figure S1: risk of bias summary. [file 5558805.f1.zip › 5558805.f1/Table S6 Respiratory system diseases(revised version).pdf]

Table S6. Respiratory system diseases (n=6).

Tai Chi styles	Tai Chi forms	Participants	Frequency (weekly)	Time (min)	Duration (week)	Intensity	Conclusion	References
Yang-style Tai Chi (3, 50%)	Simplified 24-form Tai Chi (2, 33.3%)	People with COPD	3	40-50	12	NR	Positive result	[1]
		People with COPD	5	60	12	NR	Positive result	[2]
	5-form Tai Chi (1, 16.7%)	People with COPD	2	60	12	NR	Positive result	[3]
Sun-style Tai Chi (2, 33.3%)	5-form Tai Chi (1, 16.7%)	People with COPD	2	80	6	NR	Positive result	[4]
	21-form Tai Chi (1, 16.7%)	People with COPD	2	60	12	RPE (3)	Positive result	[5]
Unspecified style (1, 16.7%)	Unspecified forms (1, 16.7%)	People with COPD	4	50	20	NR	Positive result	[6]

Note: COPD = chronic obstructive pulmonary disease; RPE = rating of perceived exertion; NR = not reported.

References:

1. Zhu, S.; Shi, K.; Yan, J.; He, Z.; Wang, Y.; Yi, Q.; Huang, H. A modified 6-form Tai Chi for patients with COPD. *Complement Ther Med* **2018**, 39, 36-42, doi:10.1016/j.ctim.2018.05.007.
2. Polkey, M.I.; Qiu, Z.H.; Zhou, L.; Zhu, M.D.; Wu, Y.X.; Chen, Y.Y.; Ye, S.P.; He, Y.S.; Jiang, M.; He, B.T., et al. Tai Chi and Pulmonary Rehabilitation Compared for Treatment-Naive Patients With COPD: A Randomized Controlled Trial. *Chest* **2018**, 153, 1116-1124, doi:10.1016/j.chest.2018.01.053.
3. Yeh, G.Y.; Roberts, D.H.; Wayne, P.M.; Davis, R.B.; Quilty, M.T.; Phillips, R.S. Tai chi exercise for patients with chronic obstructive pulmonary disease: a pilot study. *Respir Care* **2010**, 55, 1475-1482.
4. Ng, L.; Chiang, L.K.; Tang, R.; Siu, C.; Fung, L.; Lee, A.; Tam, W. Effectiveness of incorporating Tai Chi in a pulmonary rehabilitation program for Chronic Obstructive Pulmonary Disease (COPD) in primary care-A pilot randomized controlled trial. *Eur J Integr Med* **2014**, 6, 248-258, doi:10.1016/j.eujim.2014.01.007.
5. Leung, R.W.; McKeough, Z.J.; Peters, M.J.; Alison, J.A. Short-form Sun-style t'ai chi as an exercise training modality in people with COPD. *Eur Respir J* **2013**, 41, 1051-1057, doi:10.1183/09031936.00036912.
6. Niu, R.; He, R.; Luo, B.L.; Hu, C. The effect of tai chi on chronic obstructive pulmonary disease: a pilot randomised study of lung function, exercise capacity and diaphragm strength. *Heart Lung Circ* **2014**, 23, 347-352, doi:10.1016/j.hlc.2013.10.057.
