# Supplementary material for: Clinical Evidence of Tai Chi Exercise Prescriptions: A Systematic Review
Source: Evid Based Complement Alternat Med. 2021 Mar 10;2021:5558805. doi: 10.1155/2021/5558805 (PMC7972853; doi:10.1155/2021/5558805)
Supplement: Supplementary Materials — Table S1: basic characteristics of the included studies. Table S2: musculoskeletal system or connective tissue diseases. Table S3: circulatory system diseases. Table S4: mental and behavioral disorders. Table S5: nervous system diseases. Table S6: respiratory system diseases. Table S7: endocrine, nutritional, or metabolic diseases. Table S8: neoplasms. Table S9: other disease conditions. Table S10: healthy populations. Figure S1: risk of bias summary. [file 5558805.f1.zip › 5558805.f1/Table S7 Endocrine, nutritional or metabolic diseases(revised version).pdf]

Table S7. Endocrine, nutritional or metabolic diseases (n=5).

Tai Chi styles	Tai Chi forms	Participants	Frequency (weekly)	Time (min)	Duration (week)	Intensity	Conclusion	References
Multiple styles (Yang style; Sun style) (3, 60%)	12-form Tai Chi (1, 20%)	Patients with type 2 diabetes	2	60	16	HR (83.3 ± 13.7); RPE (9-13)	Negative result	[1]
	12-form Tai Chi (1, 20%)	Patients with type 2 diabetes	2	60	16	NR	Negative result	[2]
	Unspecified forms (1, 20%)	Middle-aged male office workers with metabolic syndrome	2	50	12	NR	Positive result	[3]
Yang-style Tai Chi (1, 20%)	Simplified 24-form Tai Chi (1, 20%)	Patients with type 2 diabetes	5	60	14	67.1% ± 5.13% HR_{max}	Positive result	[4]
KaiMai-style Tai Chi (1, 20%)	Unspecified forms (1, 20%)	Patients with type 2 diabetes	3	90	12	NR	Positive result	[5]

Note: HR = heart rate; RPE = rating of perceived exertion; NR = not reported.

References:

1. Tsang, T.; Orr, R.; Lam, P.; Comino, E.; Singh, M.F. Effects of Tai Chi on glucose homeostasis and insulin sensitivity in older adults with type 2 diabetes: a randomised double-blind sham-exercise-controlled trial. *Age Ageing* **2008**, 37, 64-71, doi:10.1093/ageing/afm127.
2. Tsang, T.; Orr, R.; Lam, P.; Comino, E.J.; Singh, M.F. Health benefits of Tai Chi for older patients with type 2 diabetes: the "Move It For Diabetes study"--a randomized controlled trial. *Clin Interv Aging* **2007**, 2, 429-439.
3. Choi, Y.S.; Song, R.; Ku, B.J. Effects of a T'ai Chi-Based Health Promotion Program on Metabolic Syndrome Markers, Health Behaviors, and Quality of Life in Middle-Aged Male Office Workers: A Randomized Trial. *J Altern Complement Med* **2017**, 23, 949-956, doi:10.1089/acm.2017.0057.
4. Zhang, Y.; Fu, F.H. Effects of 14-week Tai Ji Quan exercise on metabolic control in women with type 2 diabetes. *Am J Chin Med* **2008**, 36, 647-654, doi:10.1142/S0192415X08006119.
5. Liu, X.; Miller, Y.D.; Burton, N.W.; Chang, J.H.; Brown, W.J. The effect of Tai Chi on health-related quality of life in people with elevated blood glucose or diabetes: a randomized controlled trial. *Qual Life Res* **2013**, 22, 1783-1786, doi:10.1007/s11136-012-0311-7.
