# Supplementary material for: Clinical Evidence of Tai Chi Exercise Prescriptions: A Systematic Review
Source: Evid Based Complement Alternat Med. 2021 Mar 10;2021:5558805. doi: 10.1155/2021/5558805 (PMC7972853; doi:10.1155/2021/5558805)
Supplement: Supplementary Materials — Table S1: basic characteristics of the included studies. Table S2: musculoskeletal system or connective tissue diseases. Table S3: circulatory system diseases. Table S4: mental and behavioral disorders. Table S5: nervous system diseases. Table S6: respiratory system diseases. Table S7: endocrine, nutritional, or metabolic diseases. Table S8: neoplasms. Table S9: other disease conditions. Table S10: healthy populations. Figure S1: risk of bias summary. [file 5558805.f1.zip › 5558805.f1/Table S8 Neoplasms(revised version).pdf]

Table S8. Neoplasms (n=3).

Tai Chi styles	Tai Chi forms	Participants	Frequency (weekly)	Time (min)	Duration (week)	Intensity	Conclusion	References
Yang-style Tai Chi (2, 66.7%)	Simplified 24-form Tai Chi (1, 33.3%)	Lung cancer survivors	3	60	16	40%-59% HR_{max}	Positive result	[1]
	8-form Tai Chi (1, 33.3%)	Patients with lung cancer	4	60	12	NR	Positive result	[2]
Unspecified style (1, 33.3%)	19-form Tai Chi (1, 33.3%)	Senior female cancer survivors	3	60	12	NR	Positive result	[3]

Note: HR_{max} = maximum heart rate; NR = not reported.

References:

1. Zhang, Y.; Wang, R.; Chen, P.; Yu, D. Effects of Tai Chi Chuan training on cellular immunity in post-surgical non-small cell lung cancer survivors: A randomized pilot trial. *J Sport Health Sci* **2013**, 2, 104-108, doi:10.1016/j.jshs.2013.02.001.
2. Zhang, L.L.; Wang, S.Z.; Chen, H.L.; Yuan, A.Z. Tai Chi Exercise for Cancer-Related Fatigue in Patients With Lung Cancer Undergoing Chemotherapy: A Randomized Controlled Trial. *J Pain Symptom Manage* **2016**, 51, 504-511, doi:10.1016/j.jpainsymman.2015.11.020.
3. Campo, R.A.; O'Connor, K.; Light, K.C.; Nakamura, Y.; Lipschitz, D.L.; LaStayo, P.C.; Pappas, L.; Boucher, K.; Irwin, M.R.; Agarwal, N., et al. Feasibility and acceptability of a Tai Chi Chih randomized controlled trial in senior female cancer survivors. *Integr Cancer Ther* **2013**, 12, 464-474, doi:10.1177/1534735413485418.
