# Supplementary material for: Clinical Evidence of Tai Chi Exercise Prescriptions: A Systematic Review
Source: Evid Based Complement Alternat Med. 2021 Mar 10;2021:5558805. doi: 10.1155/2021/5558805 (PMC7972853; doi:10.1155/2021/5558805)
Supplement: Supplementary Materials — Table S1: basic characteristics of the included studies. Table S2: musculoskeletal system or connective tissue diseases. Table S3: circulatory system diseases. Table S4: mental and behavioral disorders. Table S5: nervous system diseases. Table S6: respiratory system diseases. Table S7: endocrine, nutritional, or metabolic diseases. Table S8: neoplasms. Table S9: other disease conditions. Table S10: healthy populations. Figure S1: risk of bias summary. [file 5558805.f1.zip › 5558805.f1/Table S9 Other disease conditions(revised version).pdf]

Table S9. Other disease conditions (n=3)

Tai Chi styles	Tai Chi forms	Participants	Frequency (weekly)	Time (min)	Duration (week)	Intensity	Conclusion	References
Yang-style Tai Chi (1, 33.3%)	8-form Tai Chi (1, 33.3%)	Elderly persons with visual impairment	3	90	16	NR	Positive result	[1]
Chen-style Tai Chi (1, 33.3%)	Unspecified forms (1, 33.3%)	Individuals with traumatic brain injury	3	45	6	NR	Positive result	[2]
Unspecified style (1, 33.3%)	20-form Tai Chi (1, 33.3%)	Patients with benign prostate hypertrophy	3	60	12	NR	Positive result	[3]

Note: NR = not reported.

References:

1. Chen, E.W.; Fu, A.S.; Chan, K.M.; Tsang, W.W. The effects of Tai Chi on the balance control of elderly persons with visual impairment: a randomised clinical trial. *Age Ageing* **2012**, 41, 254-259, doi:10.1093/ageing/afr146.
2. Gemmell, C.; Leathem, J.M. A study investigating the effects of Tai Chi Chuan: individuals with traumatic brain injury compared to controls. *Brain Inj* **2006**, 20, 151-156, doi:10.1080/02699050500442998.
3. Jung, S.; Lee, E.N.; Lee, S.R.; Kim, M.S.; Lee, M.S. Tai chi for lower urinary tract symptoms and quality of life in elderly patients with benign prostate hypertrophy: a randomized controlled trial. *Evid Based Complement Alternat Med* **2012**, 2012, 624692, doi:10.1155/2012/624692.
