# Supplementary figures and images for: Clinical Evidence of Tai Chi Exercise Prescriptions: A Systematic Review
Source: Evid Based Complement Alternat Med. 2021 Mar 10;2021:5558805. doi: 10.1155/2021/5558805 (PMC7972853; doi:10.1155/2021/5558805)

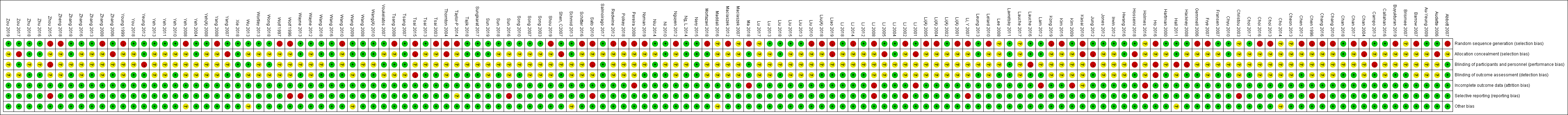

Supplement: Supplementary Materials — Table S1: basic characteristics of the included studies. Table S2: musculoskeletal system or connective tissue diseases. Table S3: circulatory system diseases. Table S4: mental and behavioral disorders. Table S5: nervous system diseases. Table S6: respiratory system diseases. Table S7: endocrine, nutritional, or metabolic diseases. Table S8: neoplasms. Table S9: other disease conditions. Table S10: healthy populations. Figure S1: risk of bias summary. [file 5558805.f1.zip › 5558805.f1/Figure S1Risk of bias summary.png]
